# Western diet contributes to the pathogenesis of non-alcoholic steatohepatitis in male mice via remodeling gut microbiota and increasing production of 2-oleoylglycerol

**DOI:** 10.1038/s41467-023-35861-1

**Published:** 2023-01-16

**Authors:** Ming Yang, Xiaoqiang Qi, Nan Li, Jussuf T. Kaifi, Shiyou Chen, Andrew A. Wheeler, Eric T. Kimchi, Aaron C. Ericsson, R. Scott Rector, Kevin F. Staveley-O’Carroll, Guangfu Li

**Affiliations:** 1 Department of Surgery, University of Missouri, Columbia, MO 65212 USA; 2 Department of Radiation Oncology, The First Affiliated Hospital of China Medical University, Shenyang, Liaoning Province 110001 China; 3 Ellis Fischel Cancer Center, University of Missouri, Columbia, MO 65212 USA; 4 Harry S. Truman Memorial VA Hospital, Columbia, MO 65201 USA; 5 Department of Veterinary Pathobiology, College of Veterinary Medicine, University of Missouri, Columbia, MO 65212 USA; 6 Department of Nutrition and Exercise Physiology, University of Missouri, Columbia, MO 65212 USA; 7 Department of Medicine-Gastroenterology and Hepatology, University of Missouri, Columbia, MO 65212 USA; 8 Department of Molecular Microbiology and Immunology, University of Missouri, Columbia, MO 65212 USA

**Keywords:** Liver fibrosis, Non-alcoholic steatohepatitis

## Abstract

The interplay between western diet and gut microbiota drives the development of non-alcoholic fatty liver disease and its progression to non-alcoholic steatohepatitis. However, the specific microbial and metabolic mediators contributing to non-alcoholic steatohepatitis remain to be identified. Here, a choline-low high-fat and high-sugar diet, representing a typical western diet, named CL-HFS, successfully induces male mouse non-alcoholic steatohepatitis with some features of the human disease, such as hepatic inflammation, steatosis, and fibrosis. Metataxonomic and metabolomic studies identify *Blautia producta* and 2-oleoylglycerol as clinically relevant bacterial and metabolic mediators contributing to CL-HFS-induced non-alcoholic steatohepatitis. In vivo studies validate that both *Blautia producta* and 2-oleoylglycerol promote liver inflammation and hepatic fibrosis in normal diet- or CL-HFS-fed mice. Cellular and molecular studies reveal that the GPR119/TAK1/NF-κB/TGF-β1 signaling pathway mediates 2-oleoylglycerol-induced macrophage priming and subsequent hepatic stellate cell activation. These findings advance our understanding of non-alcoholic steatohepatitis pathogenesis and provide targets for developing microbiome/metabolite-based therapeutic strategies against non-alcoholic steatohepatitis.

## Abstract (web summary)

Interplay of western diet and gut microbiota has been reported to be involved in the development of nonalcoholic steatohepatitis (NASH). Here the authors report that *Blautia producta* and 2-oleoylglycerol are bacterial and metabolic mediators that promote liver inflammation and hepatic fibrosis in male mice.

## Introduction

Non-alcoholic fatty liver disease (NAFLD) is an emerging global health threat and is rapidly becoming the leading cause of chronic liver disease, affecting 25% of the population worldwide^1^. The development of NAFLD ranges from simple steatosis to its advanced form, non-alcoholic steatohepatitis (NASH), which is characterized by hepatocyte injury and ballooning, liver inflammation, and liver fibrosis^2^. NASH progression can lead to subsequent cirrhotic liver disease and end-stage hepatocellular carcinoma (HCC)^3^. In recent years, increasing evidence suggests that Western-type diet (WD) and gut microbiota interact to produce metabolites that contribute to the development of NAFLD and further disease progression^4^. However, the specific bacteria and metabolites promoting NAFLD, as well as the underlying mechanisms, are not well understood. Addressing these knowledge gaps would identify therapeutic targets for this evolving healthcare crisis.

The gut and liver have close anatomical and functional communication through their connection via the portal vein. This gut-liver axis enables the transport of dietary and microbial components as well as the outcome products from the gut to the liver^5^, while the liver secretes factors such as primary bile acids that impact gut homeostasis^6^. An unhealthy lifestyle and the corresponding change in gut microbiota result in the production of many pathogenic factors which significantly impact liver immunology and homeostasis, contributing to NAFLD pathogenesis^7^. The typical WD, which is rich in fructose, sucrose, and saturated fats, is emerging as the main factor contributing to NAFLD incidence^8^. Mice fed with a high-fat diet (HFD) to model WD, reproducibly develop obesity, metabolic syndrome, and hepatic steatosis, but not NASH with liver fibrosis^9^. Numerous human and animal studies have demonstrated the important role of gut microbiota in NAFLD. A change in gut microbial composition can significantly affect hepatic carbohydrate and lipid metabolism as well as the balance between pro-inflammatory and anti-inflammatory effectors, impacting NAFLD development^10^. In addition, diet-gut microbiota interactions shape metabolism, producing a large array of bioactive metabolites with major impacts on the pathogenesis of NASH^11^. One study indicated that overgrowth of intestinal Gram-negative bacteria promotes insulin resistance and endogenous ethanol production, and induces choline deficiency, all factors implicated in NASH^12^. In addition, microbe-derived metabolites such as free fatty acids, trimethylamine, secondary bile acids, and ethanol have been reported to induce hepatocellular stress, injury, and death. This leads to fibrogenesis and genomic instability that predisposes the liver to cirrhosis and HCC^13^. Despite these advances in knowledge, further research is needed to identify unrecognized bacteria and metabolites contributing to WD-induced NAFLD and dissect the underlying mechanisms to gain a complete understanding of NAFLD pathogenesis.

In this study, we successfully established a clinically relevant murine model of NASH by feeding wild-type (WT) mice with CL-HFS, which more closely recapitulates the typical WD than a choline-deficient HFD (CD-HFD). Using this model, we identified a specific microbe, *Blautia producta* (*B. producta*), and its outcome metabolite, 2-oleoylglycerol (2-OG), which cause hepatic inflammation and fibrosis. Of particular relevance to human NASH, we detected increased accumulation of 2-OG in the livers of human NASH patients. Cellular mechanistic studies demonstrated that 2-OG is a microbial metabolite that activates hepatic stellate cells (HSCs) to produce extracellular matrix (ECM) proteins in a macrophage (MΦ)-dependent manner. Molecular studies demonstrated that 2-OG is a G protein-coupled receptor GPR119 agonist that stimulates MΦs via the TAK1/NF-κB/TGF-β1 signaling pathway. These findings significantly enhance our understanding of NASH pathogenesis and provide potential therapeutic targets.

## Results

### Development of a clinically relevant murine NASH model with CL-HFS, a typical WD

**Fig. 1:**
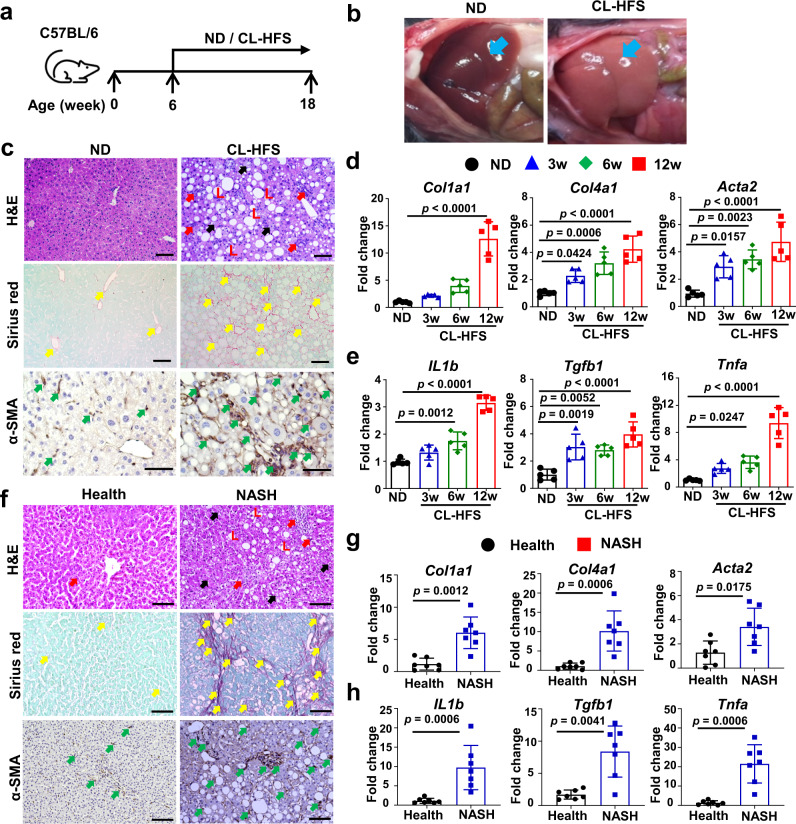
Establishment and characterization of a mouse NASH model. **a** An outline depicting induction of NASH with a CL-HFS. Six-week-old WT C57BL/6 J mice were fed a CL-HFS or normal diet (ND) for 12 weeks. At week 18, each mouse was euthanized for the following studies. **b** Representative macroscopic images of livers in CL-HFS- and ND-fed mice. **c** Representative histological images of liver tissues. Typical hepatic features of NASH were detected in CL-HFS-fed mice in comparison to ND-fed mice: H&E staining showed lipid deposition (L), hepatocytes ballooning (black arrow), and inflammatory cell liver infiltration (red arrow); Sirius red staining showed increased production of collagen (yellow arrows); IHC showed increased production of α-SMA (green arrows). Bar: 50 μm. **d** qPCR measurement of mRNA expression of extracellular matrix (ECM) genes *Col1a1*, *Col4a1*, and *Acta2*; **e** qPCR measurement of mRNA expression of proinflammatory cytokine genes *IL1b*, *Tgfb1*, and *Tnfa* in the livers of ND-fed and CL-HFS-fed mice at the indicated time points. For **d** and **e**, *n* = 5, data are presented as mean ± SD. **f** Representative histological images of liver tissues in healthy individuals and patients with NASH. Compared to healthy individuals, NASH caused lipid deposition (L), hepatocyte ballooning (black arrow), and inflammatory cell liver infiltration (red arrow) assessed by H&E staining; increased production of collagen measured by Sirius red staining (yellow arrows), and enhanced production of α-SMA (green arrows) measured by IHC. Bar: 100 μm. **g** mRNA expression of ECM genes in human livers. qPCR detected increased gene expression of *Col1a1*, *Col4a1*, and *Acta2* in the livers of patients with NASH compared to those of healthy individuals. **h** mRNA expression of proinflammatory cytokines in human livers. qPCR detected the increased gene expression of *IL1b*, *Tgfb1*, and *Tnfa* in the livers of patients with NASH compared to healthy individuals. For **g** and **h**, *n* = 7, data are presented as mean ± SD. Statistical analysis of data was performed by one-way ANOVA with Tukey’s multiple comparison test (≥3 groups) or Mann–Whitney test (two-tailed) using GraphPad Prism 8 software. Source data are provided as a Source Data file.

We have successfully developed a clinically relevant murine model of NASH using CL-HFS to approximate a typical WD. Methionine-/choline-deficient diet (MCD)^14^ and CD-HFD composed of 60 kcal% fat and 0.1% methionine without choline^15^ have been widely applied to induce NAFLD, but this diet also commonly induces liver atrophy and/or severe weight loss which do not exist in the human disease^15^. Data from the National Health and Nutrition Examination Survey (NHANES) 2009–2014 show that greater than 90% of adults, independent of age and gender, do not consume an adequate amount of choline as recommended by the US National Academies of Medicine Food and Nutrition Board^16^. Therefore, we fed WT C57BL/6 J mice with a CL-HFS to better represent the typical WD than CD-HFD (no choline). We found that CL-HFS consumption (Fig. 1a) caused an increase in liver size and visibly lighter liver color (Fig. 1b) at week 12 and a significant increase in body weight at week 36 (Supplementary Fig. 1a). Blood biochemistry assays showed a significant increase in serum levels of alanine aminotransferase (ALT), aspartate aminotransferase (AST), and cholesterol (Supplementary Fig. 1b). H&E staining revealed a noticeable increase in the infiltration of inflammatory cells, accumulation of lipid droplets, and hepatocyte ballooning, resulting in an increased NAFLD Activity Score (NAS) (Fig. 1c and Supplementary Fig. 1c, d). Sirius red staining detected significantly increased production of collagen, forming pericellular and bridging hepatic fibrosis (Fig. 1c and Supplementary Fig. 1d). IHC detected significantly increased expression of α-SMA, a marker of activated HSCs (Fig. 1c and Supplementary Fig. 1d). qPCR detected a significant increase in mRNA expression of extracellular matrix (ECM) genes including *Col1a1*, *Col4a1*, and *Acta2* (Fig. 1d) and inflammatory cytokine genes including *IL1b*, *Tgfb1*, and *Tnfa* (Fig. 1e). These alterations were consistent with human NASH (Fig. 1f–h and Supplementary Fig. 1e)^13^. Furthermore, qPCR detected significantly increased expression of *IL6*, *Ccl2*, *Myd88*, and *Nfkb* (Supplementary Fig. 1f). In addition, CL-HFS induced a significant increase in visceral fat, but not subcutaneous fat (Supplementary Fig. 2b-d). We did not detect statistically significant expression of inflammatory cytokines and adipokines in visceral fat between CL-HFS-fed mice and ND-fed mice (Supplementary Fig. 2e). These results suggest that adipose tissue induced by CL-HFS may not be a major contributor to the development and progression of NASH in these mice. These histological, immunohistochemical, biochemical, and molecular analyses suggest that CL-HFS consumption induces fatty liver, liver injury, an innate hepatic inflammatory response, ECM gene upregulation, and liver fibrosis, as well as obesity, reflecting typical NASH features in human disease. This model is therefore clinically relevant, providing a platform to study NASH pathogenesis and its underlying mechanisms.

### ABX treatment preventively and therapeutically suppresses the development of CL-HFS-induced NASH

**Fig. 2:**
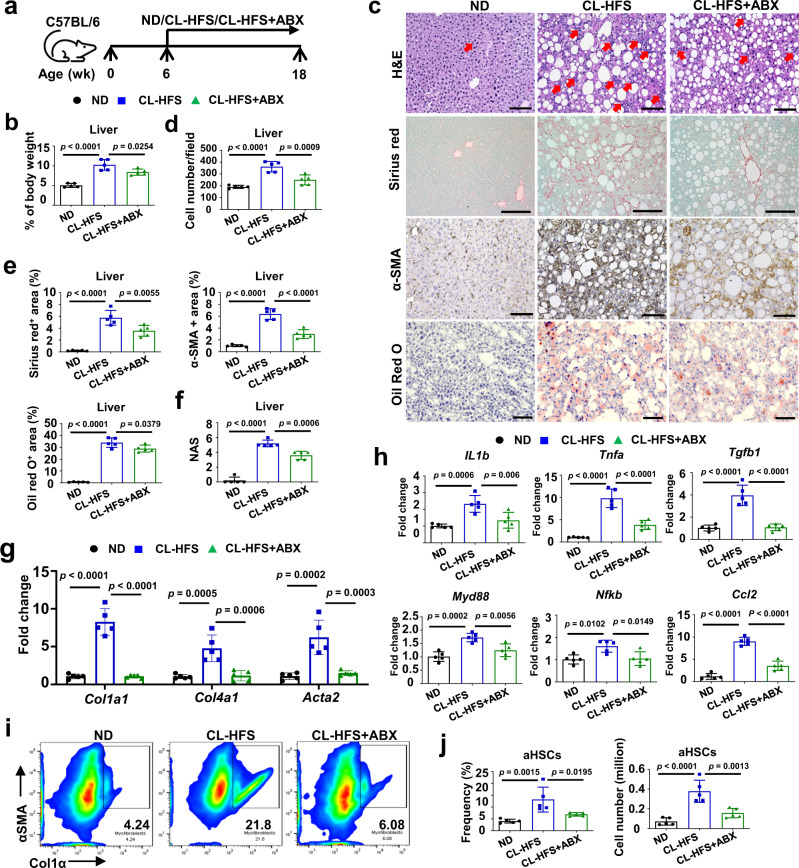
ABX treatment slows the development of CL-HFS-induced NASH. **a** An outline depicting ABX treatment design. Six-week-old WT C57BL/6 J mice were fed with a CL-HFS for 12 weeks with or without simultaneous ABX treatment, then euthanized for the following studies. ND-fed mice were used for controls. **b** Effect of ABX on CL-HFS-induced increase in the ratios of liver-to-bodyweight. Compared to ND, CL-HFS caused an increase in the liver-to-bodyweight ratio which was suppressed by ABX treatment. **c** Effect of ABX on CL-HFS-induced NASH. Compared to untreated mice, ABX treatment led to an obvious reduction in the liver infiltration of inflammatory cells (red arrow) (H&E staining), production of collagen (Sirius red staining), α-SMA (IHC detection), and lipid accumulation (Oil red O staining) in CL-HFS-fed mice. Bar: 100 μm. **d** Semi-quantification of inflammatory cells infiltratiing into the liver; **e** Semi-quantification of collagen production, α-SMA protein expression, and lipid accumulation in the livers of three groups of mice shown in **c**. **f** NAFLD activity score (NAS). ABX treatment led to reduced NAS in CL-HFS-fed mice. **g** Hepatic mRNA expression of ECM genes in CL-HFS-fed mice with or without ABX treatment. qPCR measured reduced mRNA expression of *Col1a1*, *Col4a1*, and *Acta2* in the livers of ABX-treated mice compared to untreated mice. **h** Hepatic mRNA expression of proinflammatory cytokines, profibrotic cytokines, and chemokine. qPCR measured reduced mRNA expression of *IL1b*, *Tgfb1*, *Tnfa*, *Myd88*, *Nfkb*, and *Ccl2* in the livers of ABX-treated mice compared to untreated mice. ABX suppressed activation of HSCs. Representative (**i**) and cumulative results (**j**) of flow cytometric assays indicated that ABX treatment reduced the frequency of activated HSCs expressing Col1a and α-SMA in CL-HFS-fed mice. *n* = 5, data are presented as mean ± SD. Statistical analysis of data was performed by one-way analysis of variance (ANOVA) with Tukey’s multiple comparison test using GraphPad Prism 8 software. Source data are provided as a Source Data file.

Abnormal gut microbiota is an environmental risk factor that significantly contributes to the progression and development of NAFLD^10,17^. Previous studies have shown that a cocktail of antibiotics (ABX) causes changes in a relative abundance of murine gut microbiota without significant hepatotoxicity^18,19^. To test whether these changes could prevent the development of NASH, we treated CL-HFS-fed mice with ABX via drinking water (Fig. 2a). Consistent with other literature reports^18,19^, ABX treatment did not cause significant hepatotoxicity and did not promote liver inflammation and fibrosis (Supplementary Fig. 3). In addition, ABX had no impact on mouse body weight and food consumption (Supplementary Fig. 3). By measuring body weight and liver weight, performing H&E staining, counting infiltrating inflammatory cells, and conducting Sirius red staining, Oil red O staining, IHC, and qPCR, we demonstrated that ABX treatment suppressed CL-HFS-induced increase in the ratio of liver-to-bodyweight (Fig. 2b), reduced the frequency of liver resident inflammatory cells (Fig. 2c, d), and decreased the hepatic production of collagen, α-SMA, and lipid accumulation (Fig. 2c, e), resulting in a reduction of NAS (Fig. 2f). These alterations were accompanied by an ABX-induced suppression of hepatic expression of ECM genes including *Col1a1*, *Col4a1*, and *Acta2* (Fig. 2g) and reduction of the inflammatory response, evidenced by significantly reduced expression of inflammatory cytokines, chemokines, and signaling genes including *IL1b*, *Tnfa*, *Tgfb1*, *Ccl2*, *Myd88*, and *Nfkb* (Fig. 2h). Because HSCs contribute to approximately 82%-96% of liver myofibroblasts in toxic and fatty liver disease models^20^, we next examined whether ABX treatment suppresses activation of HSCs. Activated HSCs express α-SMA and Col1α^21^, two hallmark features of myofibroblasts. CL-HFS consumption resulted in a more than three-fold increase in the frequency and cell number of HSCs expressing α-SMA and Col1α compared to ND; this increase was significantly suppressed by ABX treatment (Fig. 2i, j). However, ABX did not show a direct effect on the suppression of HSC activation (Supplementary Fig. 3, 4). These results suggest that ABX-mediated changes in the gut microbiota play a pivotal role in CL-HFS-induced NASH by suppressing inflammatory responses, liver fibrosis, and HSC activation.

Next, we examined the therapeutic potential of ABX to suppress CL-HFS-induced NASH. WT mice were fed CL-HFS for 12 weeks to induce NASH followed by 6 weeks of ABX treatment in drinking water to induce selective suppression of gut microbiota (Supplementary Fig. 5a). While therapeutic ABX treatment did not cause a significant decrease in the hepatic lipid accumulation (*p* = 0.31), it did significantly reduce the ratio of liver-to-bodyweight, hepatic production of collagen and α-SMA, NAS, mRNA expression of ECM genes *Col4a1* and *Acta2* and proinflammatory cytokines *Tgfb1*, *Ccl2*, and *IL1b*, as well as the average frequency and cell number of activated HSCs expressing Col1α and α-SMA (Supplementary Fig. 5b–i). These results suggest significant potential for ABX to suppress NASH.

### ABX treatment modulates CL-HFS-induced changes in gut microbiota and hepatic metabolites

**Fig. 3:**
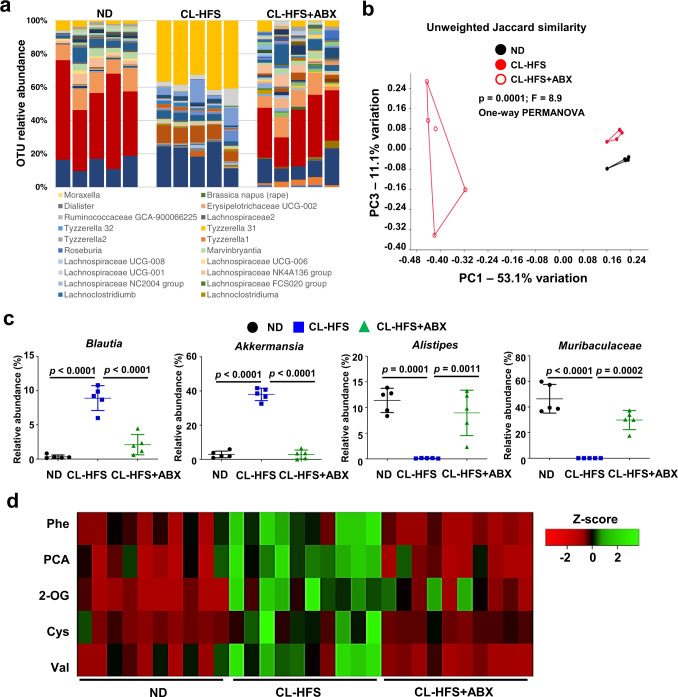
ABX treatment alters the profiles of gut microbiota and hepatic metabolites. Fecal and liver samples were collected from Fig. 2 mice, which were fed with ND or CL-HFS in the absence or presence of ABX for 12 weeks. **a** Effect of ABX on the relative abundance of operational taxonomic unit (OUT) in CL-HFS-fed mice. 16 S rRNA gene sequencing of fecal samples identified the profiles of gut microbiota in three groups of mice. **b** ABX treatment changed gut microbiota similarity. PERMANOVA significance test was performed with Principal-coordinate analysis (PCA) to define the Jaccard similarity index. **c** ABX induced a significant alteration in representative bacterial species. ABX treatment significantly reduced the relative abundance of *Blautia* and *Akkermansia* and increased the relative abundance of *Alistipes* and *Muribaculaceae* in the fecal samples of CL-HFS-fed mice. *n* = 5, data are presented as mean ± SD. **d** ABX changed the hepatic metabolite profile. Hepatic metabolites in three groups of mice were analyzed by non-targeted Gas chromatography-mass spectrometry (GC-MS). Heatmap showed Z-scores of 5 metabolites in 30 liver tissues from 10 ND-fed mice, 10 CL-HFS-fed mice, and 10 CL-HFS-fed mice with ABX treatment. ABX treatment markedly reduced the following metabolite production in CL-HFS-fed mice, L-Phenylalanine (Phe), Pyroglutamic acid (PCA), 2-Oleoylglycerol (2-OG), Cysteine (Cys), and L-Valine (Val). *n* = 10, data are presented as mean ± SD. Statistical analysis of data was performed by one-way ANOVA with Tukey’s multiple comparison test using GraphPad Prism 8 software. Source data are provided as a Source Data file.

To investigate the impact of ABX on gut microbiota in the mice that experienced prevention and suppression of CL-HFS-induced NASH, we collected fecal samples from mice in the experiments above (Fig. 2). 16 S rRNA gene sequencing and a relative abundance plot based on the operational taxonomic units (OTU) showed that CL-HFS-caused the noticeable change in gut microbiota composition was suppressed by ABX treatment, resulting in the relative abundance of bacterial families seen in ND-fed mice (Fig. 3a). This change was supported by the beta-diversity analysis (Fig. 3b). The relative abundance of four genera of the gut microbiota with the most obvious alterations among the three groups of mice were shown in Fig. 3c. The results indicated that consumption of CL-HFS led to a relatively increased abundance of *Blautia* (phylum, Firmicutes) and *Akkermansia* (phylum, Verrucomicrobia) and a decrease of *Alistipes* (phylum, Bacteroidetes) and *Muribaculaceae* (phylum, Bacteroidetes). ABX treatment suppressed this CL-HFS-caused alteration of the gut microbiota with increased frequency of *Alistipes* and *Muribaculaceae* and reduced frequency of *Blautia* and *Akkermansia*. (Fig. 3c). These results suggest that ABX suppresses CL-HFS-caused NASH pathogenesis, in association with the manipulation of gut microbiota.

The gut microbiota is strongly linked to systemic metabolism. Therefore, ABX-induced remodeling of gut microbiota may alter hepatic metabolism in CL-HFS-fed mice. We harvested livers from ND- and CL-HFS-fed mice treated with ABX or control (Fig. 2) and used them for analysis of hepatic metabolism by non-targeted gas chromatography-mass spectrometry (GC-MS). Results were analyzed with one-way ANOVA test followed by correction with Tukey’s multiple comparison test, and there was a significant change in 50 out of 309 detected metabolites in the three groups (Supplementary Fig. 6). Among the metabolites with a significant difference between ND-fed mice and CL-HFS-fed mice which were shown with red dots, five metabolites, L-phenylalanine (Phe), pyroglutamic acid (PCA), 2-OG, cysteine (Cys), and L-valine (Val), had obvious increases in response to CL-HFS consumption, which was abrogated by ABX treatment (Fig. 3d). Other altered metabolites were either unknown or did not show a significant response to ABX treatment (Supplementary Fig. 6 and Supplementary Table 1, 2). Together, these results suggest that both CL-HFS and ABX re-shape gut microbiota, significantly impacting the hepatic metabolome.

### Increased fecal *Blautia* and hepatic 2-OG are also detected in human patients with NASH

To further establish the clinical relevance of our findings, we investigated the similarities and discrepancies in gut microbiota composition between mice and humans in response to HFS consumption. Using a publicly available human microbiome database for patients with NAFLD from BioProject PRJNA540738 (https://www.ncbi.nlm.nih.gov/bioproject/540738), we performed a comparative analysis of gut microbiota between healthy individuals and human NAFLD patients. Focusing on Firmicutes and Bacteriodetes, which are the top two bacterial phyla existing in both mice and humans^22^, we found a significant reduction of Bacteriodetes (Supplementary Fig. 7a) and a slight, non-significant increase of Firmicutes (Supplementary Fig. 7b) in NAFLD patients. However, *Lachnospiraceae*, one family of the phylum Firmicutes, was significantly increased (Supplementary Fig. 7c). *Blautia*, one genus of the family *Lachnospiraceae*, was highly enriched in human patients with NAFLD relative to healthy subjects^23^. In addition, a recent study identified the family *Lachnospiraceae* as a signature taxon for primary HCC that significantly increased in the tumor regions compared to non-tumor regions in the same HCC patients^24^. This finding is consistent with the abundance of *Blautia* in our CL-HFS-fed mouse model, suggesting a common role of *Blautia* in humans and mice in contribution to NASH pathogenesis and HCC.

**Fig. 4:**
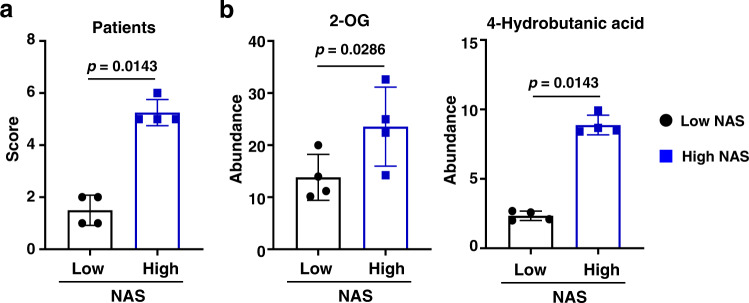
Discrepancies of liver metabolites in patients with obesity and with or without NASH. **a** NAS in patients with obesity and with or without NASH. According to NAS, eight patients with obesity were divided into two groups: patients with obesity and NAS > 4 (high) and individuals with obesity and NAS < 4 (low). **b** Significantly increased production of hepatic 2-OG and 4-Hydroxybutanoic acid in patients with obesity and high NAS compared to that in patients with obesity and low NAS. *n* = 4, data are presented as mean ± SD. Statistical analysis of data was performed by Mann–Whitney test (one-tailed) using GraphPad Prism 8 software. Source data are provided as a Source Data file.

Next, we investigated hepatic metabolite alteration in human NASH patients. Specifically, we focused on the five metabolites identified in NASH mice with the most obvious alteration in response to both CL-HFS and ABX treatment. We collected liver biospecimens from eight patients with obesity and with or without histologically confirmed NASH for metabolic assays (Fig. 4a). Non-targeted GC-MS detected significantly increased 2-OG in patients with obesity and NASH relative to those without NASH (Fig. 4b), while no such correlation was seen with other four metabolites (Phe, PCA, Cys, and Val). In addition, we detected a significantly increased 4-hydroxybutanoic acid (Fig. 4b). In conclusion, our pre-clinical and clinical studies consistently demonstrate that both *Blautia* and 2-OG are increased in both mice and human patients with NASH, likely functioning as NASH-promoting bacterium and metabolite.

It is well-known that metabolites, as metabolic mediators, impact the pathophysiology of NAFLD/NASH^25^. Studies have demonstrated that bacterial lipase can digest triacylglycerol to produce 2-OG^26^. *B. producta*, one species of the genus *Blautia*, was increased in CL-HFS-fed mice, but not in low-fat diet-fed mice (Supplementary Fig. 8). By culturing *B. producta* in anaerobic conditions, we detected a significantly increased production of 2-OG in the culture medium by GC-MS analysis of 2-OG concentration (Supplementary Fig. 9a). Circulating 2-OG in serum also increased in CL-HFS-fed mice compared to that in ND-treated mice or ABX-treated CL-HFS-fed mice (Supplementary Fig. 9b). In addition, in vivo studies demonstrated that the repopulation of ABX5-sterilized mice with *B. producta* led to an increased hepatic accumulation of 2-OG (Supplementary Fig. 9c). Together, these results indicate that 2-OG is a metabolite generated from the interaction of CL-HFS and lipase-producing bacteria associated with NASH development.

### *B. producta* promotes liver resident MΦ and HSC activation in CL-HFS-fed mice

**Fig. 5:**
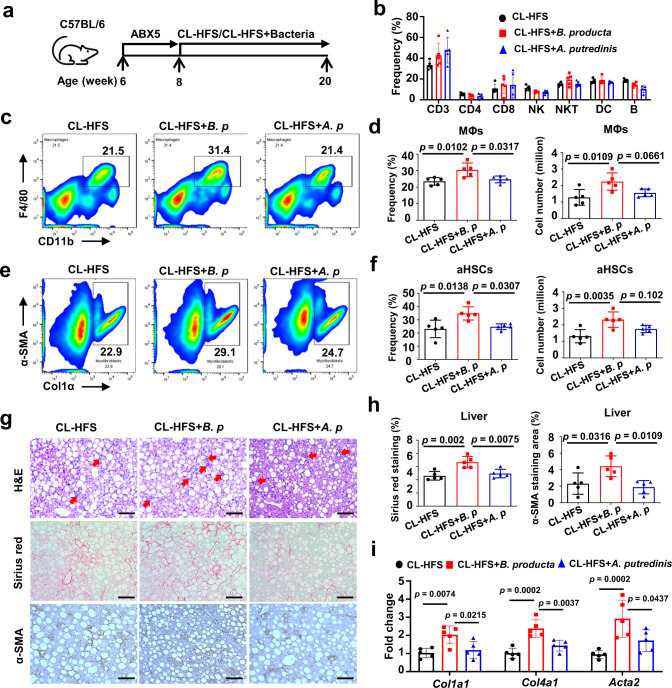
*B. producta* repopulation promotes CL-HFS-induced liver fibrosis in association with modulation of liver-resident MΦs. **a** An outline depicting gut microbiota sterilization and bacterial repopulation. Six-week-old WT C57BL/6 J mice received ABX5 orally in drinking water for two weeks to deplete intestinal bacteria, then received CL-HFS and simultaneous *Blautia producta* (ATCC 27340) or *Alistipes putredinis* (ATCC 29800) repopulation by oral gavage twice a week at a dose of 3 × 10^8^ CFU/mouse in 200 µl of PBS. Twelve weeks later, all mice were euthanized to harvest livers and isolate hepatic NPCs for the following studies. **b** The frequencies of distinct hepatic immune cells. Liver NPCs underwent flow cytometry to define the frequencies of liver-resident CD3 (CD3^+^), CD4 (CD3^+^CD4^+^), CD8 (CD3^+^CD8^+^), NK (CD3^-^CD49b^+^), NKT (CD3^+^NK1.1^+^), DCs (CD11b^+^CD11c^+^), and B cells (CD3^-^B220^+^). **c** Representative frequencies of liver-resident MΦs in NPCs. **d** Mean frequency (left) and absolute number (right) of hepatic MΦs in **c**. Data showed that repopulation with *B. producta*, but not *A. putredinis*, increased the frequency of hepatic MΦs in CL-HFS-fed mice. **e** Representative frequencies of activated HSCs. **f** Mean frequency (left) and absolute number (right) of activated HSCs in **e**. Data indicated that repopulation with *B. producta*, but not *A. putredinis*, increased the frequency of activated HSCs expressing Col1α and α-SMA in CL-HFS-fed mice. **g** Liver infiltration of inflammatory cells (H&E staining), collagen production (Sirius red staining), and α-SMA production (IHC staining). Red arrows point to inflammatory cells. Sirius red staining and IHC staining detected increased production of collagen and α-SMA in the mice with *B. producta* repopulation versus control mice with or without *A. putredinis* repopulation. Bar: 100 μm. **h** Semi-quantification (Mean percentage) of areas positive for Sirius red and α-SMA staining. **i** Expression of ECM genes. qPCR detected an increased mRNA expression of *Col1a1*, *Col4a1*, and *Acta2* in the mice with *B. producta* repopulation versus control mice with or without *A. putredinis* repopulation. *n* = 5, data are presented as mean ± SD. Statistical analysis of data was performed by one-way ANOVA with Tukey’s multiple comparison test using GraphPad Prism 8 software. Source data are provided as a Source Data file.

Given the positive correlation of *Blautia* and 2-OG with mouse and human NASH and our findings that the lipase-producing species *B. producta* can produce 2-OG, we further investigated the contribution of this species to NASH pathogenesis. ABX5 has been used to broadly deplete gut microbiota in mice with minimal effects on morbidity and mortality^27^. Having demonstrated that treating WT mice with ABX5 did not induce a significant effect on liver inflammation and fibrogenesis (Supplementary Fig. 3), we treated mice with ABX5 for two weeks, then gave mice oral gavage of *B. producta* while feeding CL-HFS (Fig. 5a). *Alistipes putredinis (A. putredinis)* was selected as a control, as *Alistipes* changes inversely to *Blautia* in response to CL-HFS consumption and ABX treatment. Twelve weeks later, hepatic non-parenchymal cells (NPCs) from each mouse were isolated for flow cytometric assay. The results indicated that repopulation with *B. producta*, but not *A. putredinis*, significantly increased the frequency and cell number of liver resident MΦs expressing CD11b and F4/80 in CL-HFS-fed mice (Fig. 5c, d). This alteration was not observed in other types of immune cells including CD3^+^T cells, CD4^+^CD3^+^T cells, CD8^+^CD3^+^T cells, NK1.1^+^CD3^+^ natural killer T (NKT) cells, CD3^-^B220^+^ B cells, or CD49b^+^CD3^-^ natural killer (NK) cells (Fig. 5b). In addition, *B. producta* repopulation led to a significant increase in the frequency and cell number of activated HSCs expressing α-SMA and Col1α (Fig. 5e, f). Histological analysis, Sirius red staining, and IHC staining showed that *B. producta* repopulation led to an increase in hepatic infiltration of inflammatory cells and production of collagen and α-SMA (Fig. 5g, h). Additionally, qPCR detected increased expression of ECM genes *Col1α1* , *Col4a1*, and *Acta2* (Fig. 5i). These results suggest that *B. producta* can activate MΦs and HSCs and enhance hepatic fibrosis during CL-HFS-induced NASH. By sterilizing the guts of mice with established CL-HFS-induced NASH followed by *B. producta* repopulation (Supplementary Fig. 10a), we demonstrated that *B. producta* further potentiates CL-HFS-induced activation of MΦ and HSCs (Supplementary Fig. 10b-e). Moreover, *B. producta* supplementation promoted liver fibrosis and increased liver resident MΦs in ND-fed mice (Supplementary Fig. 11). Together, these results confirm that *B. producta* functions as a NASH-promoting bacterium, activating MΦs and HSCs during the initiation and progression of NASH in both ND and CL-HFS-fed mice.

### 2-OG, as a single metabolite, activates HSCs upon MΦ priming

**Fig. 6:**
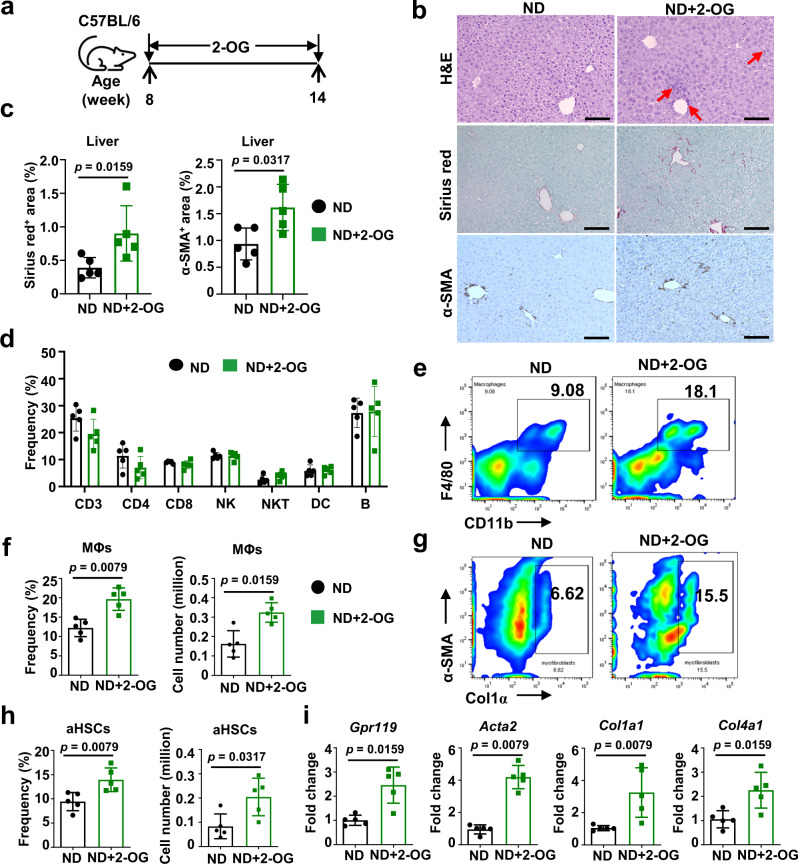
2-OG causes liver fibrosis in ND-fed mice in association with modulation of liver resident MΦs. **a** An outline depicting the experimental design of 2-OG treatment. Eight-week-old WT C57BL/6 J mice fed with ND received i.v. injection of 2-OG three times a week for 6 weeks at a dose of 20 μg/mouse in 0.2 mL PBS. PBS injection was used for control. After that, all mice underwent liver perfusion to isolate hepatic NPCs for the following studies. **b** Liver infiltration of inflammatory cells (H&E staining), collagen production (Sirius red staining), and α-SMA production (IHC staining). Red arrows point to inflammatory cells. Bar: 100 μm. **c** Semi-quantification of areas positive for Sirius red staining and α-SMA IHC staining. Semi-quantification showed the increased Sirius red staining area and α-SMA IHC staining area in 2-OG-treated mice versus control mice. **d** Frequencies of different types of immune cells in the liver. Liver NPCs underwent flow cytometry to define mean frequencies of different types of immune cells including CD3 (CD3^+^), CD4 (CD3^+^CD4^+^), CD8 (CD3^+^CD8^+^), NK (CD3^-^CD49b^+^), NKT (CD3^+^NK1.1^+^), DCs (CD11b^+^CD11c^+^), and B cells (CD3^-^B220^+^). **e** Representative frequencies of liver-resident MΦs in NPCs. Representative flow cytometry analysis showed that 2-OG injection caused an increased frequency of liver-resident MΦs. **f** Mean frequency (left) and absolute number (right) of MΦs in NPCs in control and 2-OG-treated mice as shown in **e**. **g** Representative frequencies of activated HSCs expressing Col1α and α-SMA in liver NPCs. Representative flow cytometry analysis showed that 2-OG injection caused an increased frequency of activated HSCs expressing Col1α and α-SMA in liver NPCs. **h** Mean frequency (left) and absolute number (right) of activated HSCs expressing Col1α and α-SMA in NPCs in control and 2-OG-treated mice as shown in **g**. **i** mRNA expressions of liver *Gpr119* and *Acta2*. qPCR showed that 2-OG injection led to the increased mRNA expression of *Gpr119*, *Acta2*, *Col1a1*, and *Col4a1* in livers. *n* = 5, data are presented as mean ± SD. Statistical analysis of data was performed by Mann–Whitney test (two-tailed) using GraphPad Prism 8 software. Source data are provided as a Source Data file.

Next, we investigated whether 2-OG functions as a metabolic mediator inducing hepatic inflammation and HSC activation. To test this hypothesis, ND-fed mice received i.v. administration of 2-OG three times a week for 6 weeks at a dose of 20 μg/mouse in 0.2 mL PBS (Fig. 6a). This dose of 2-OG was selected because it did not show cytotoxicity and resulted in a hepatic 2-OG concentration equivalent to that in patients with obesity and NASH (Supplementary Fig. 12). The results indicate that 2-OG treatment increased hepatic infiltration of inflammatory cells and production of collagen and α-SMA (Fig. 6b, c). In addition, hepatic NPCs were isolated from each mouse and analyzed by flow cytometric assay. While 2-OG administration did not induce detectable alteration in the frequency of other lymphocytes (Fig. 6d), the frequencies of CD11b^+^F4/80^+^MΦs (Fig. 6e, f) and α-SMA^+^Col1α^+^HSCs (Fig. 6 g, h) increased by approximately 2-fold in 2-OG-treated mice compared to control mice. qPCR detected more than 2-fold increased expression of *Acta2*, *Col1a1*, and *Col4a1* as well as *Gpr119* (Fig. 6i). Increased hepatic expression of *Gpr119* and *Cd68*, but not *Cnr1* (Supplementary Fig. 13a, b) was also detected in mice with CL-HFS-induced NASH and human patients with obesity and NASH. 2-OG has been recognized as an agonist of GPR119 that mediates lipid effects^28,29^. Furthermore, we have shown that GPR119 is mainly expressed by liver resident MΦs as opposed to hepatocytes in livers with NASH (Supplementary Fig. 14). These results suggest that 2-OG may act on MΦs through GPR119 signaling.

To investigate whether MΦ and HSC, as a cellular basis, mediate 2-OG-caused NASH pathogenesis, we stimulated single or co-cultured mouse immortalized HSCs and RAW264.7 cells with 2-OG for 24 h. Expression of *Acta2*, *Col1a1*, and *Col4a1*, as measured by qPCR, did not change in single HSCs and RAW264.7 cells (Supplementary Fig. 15a, b), but increased in co-cultured cells (Supplementary Fig. 15c); this change was accompanied by a significant increase of *Gpr119*, *IL1b*, and *Tgfb1* (Supplementary Fig. 15d). Flow cytometric assay revealed that α-SMA and Col1α were mainly produced by the HSCs, and GPR119 was mainly produced by RAW264.7 cells (Supplementary Fig. 15e-h). 2-OG also stimulated a significant increase in the frequency of MΦs in hepatic NPCs and splenocytes from WT mice (Supplementary Fig. 16a-d). However, these effects were not found for the other four altered metabolites (Cys, PCA, Phe, Val), or the control metabolite serine relevant to CL-HFS (Supplementary Fig. 16e-f). In addition, we demonstrated that 2-OG did not potentiate TGF-β1-induced HSC activation to stimulate further increases of *Col1a1*, *Col4a1*, and *Acta2* over TGF-β1 alone (Supplementary Fig. 17). These results suggest that 2-OG as a metabolic mediator activates HSCs in an MΦ-dependent way.

### 2-OG, as a GPR119 agonist, primes MΦs via TAK1/NF-κB/TGF-β1 signaling pathway

**Fig. 7:**
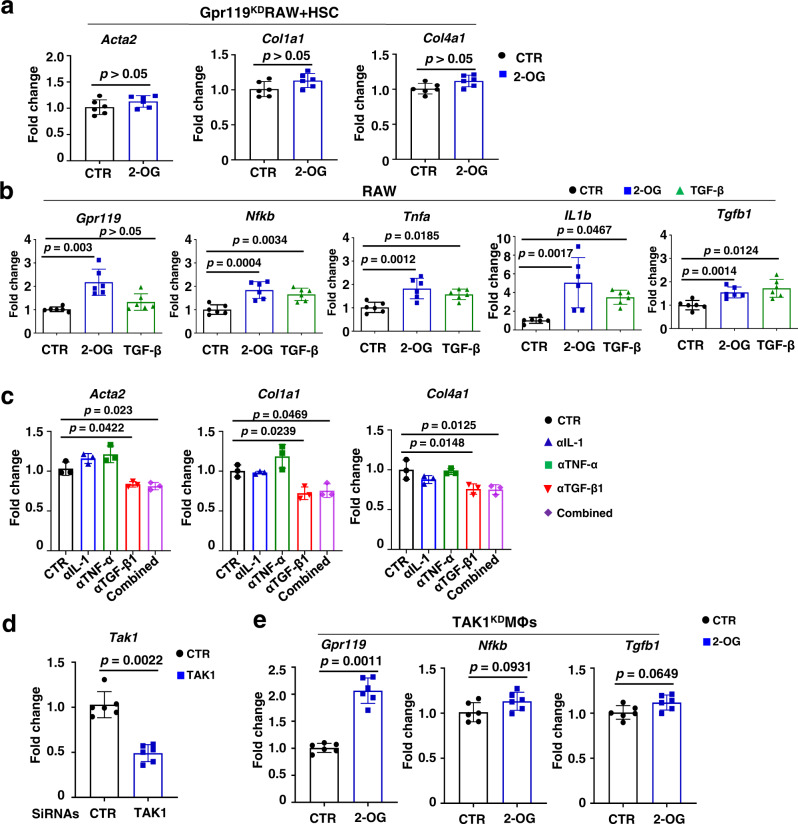
2-OG is unable to activate GPR119-knockdown MΦs and their co-cultured HSCs. **a** *Gpr119*-knockdown in MΦs blocks 2-OG-induced activation of the co-cultured HSCs. qPCR did not detect the 2-OG-induced upregulation of genes *Acta2*, *Col1a1*, and *Col4a1* in HSCs co-cultured with *Gpr119*-knockdown RAW264.7 cells induced by siRNAs. **b** 2-OG stimulation induces increased production of GPR119 and proinflammatory cytokines in RAW264.7 cells. qPCR detected increased mRNA expression of *Gpr119*, *Nfkb*, *Tnfa*, *IL1b*, and *Tgfb1* in 2-OG-stimulated RAW264.7 cells for 24 h. TGF-β1 (10 ng/mL) was used as a positive control. **c** Anti-TGF-β1 inhibits 2-OG-induced HSC activation co-cultured with RAW264.7 cells. qPCR analysis showed that 2-OG stimulation upregulated *Acta2*, *Col1a1*, and *Col4a1* in HSCs co-cultured with RAW264.7 cells, which was inhibited by antibodies for TGF-β1 (1 µg/mL) but not for IL-1 (1 µg/mL) or TNF-α (1 µg/mL). **d** Validation of siRNA for *Tak1* knockdown. qPCR detected the significantly decreased *Tak1* expression in RAW264.7 cells treated with *Tak1* siRNA versus scramble siRNA. **e** 2-OG stimulation is unable to promote *Nfkb* and *Tgfb1* expression in MΦs with *Tak1* knockdown. qPCR detection showed that 2-OG stimulation significantly increased expression of *Gpr119*, but not *Nfkb* and *Tgfb1*, in *Tak1*-knockdown RAW264.7 cells. For **a**, **d** and **e**, *n* = 6; for **b** and **c**, *n* = 3. Data are presented as mean ± SD. The assay was repeated twice. Statistical analysis of data was performed by one-way ANOVA with Tukey’s multiple comparison test (≥3 groups) or by Mann–Whitney test (two-tailed) using GraphPad Prism 8 software. Source data are provided as a Source Data file.

**Fig. 8:**
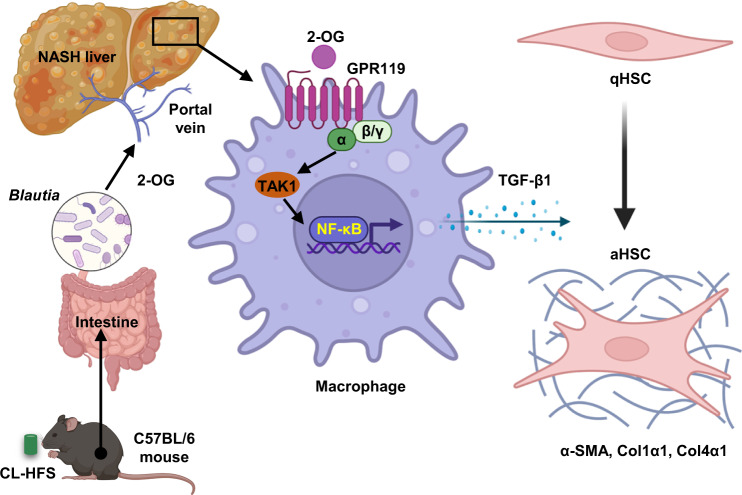
Schematic diagram of 2-OG-mediated HSC activation in an MΦ-dependent manner. CL-HFS and *Blautia* interplay produces 2-OG, which stimulates MΦ expression of TGF-β1 through GPR119-TAK1-NF-κB signaling. The resultant TGF-β1 activates quiescent HSCs (qHSCs) to activated HSCs (aHSCs) to increase the expression of ECM genes, including *Acta2*, *Col1a1*, and *Col4a1*. This figure was created using Biorender (https://biorender.com).

These provocative results drove us to investigate whether GPR119, as a molecular basis, mediates the 2-OG-induced priming of MΦs. We evaluated the ability of three different siRNAs for mouse GPR119 to suppress its expression (Supplementary Fig. 18a, b). Using the functionally validated siRNAs, we demonstrated that siRNA-mediated GPR119 knockdown in RAW264.7 cells abrogated the 2-OG-induced upregulation of ECM genes including *Acta2*, *Col1a1*, and *Col4a1* in co-cultured HSCs (Fig. 7a), suggesting GPR119 is necessary for 2-OG-induced MΦ activation. qPCR assay demonstrated that 2-OG stimulation significantly enhanced the expression of *IL1b*, *Tnfa*, *Tgfb1*, *Nfkb*, and *Gpr119* in RAW264.7 cells (Fig. 7b) and peritoneal MΦs (Supplementary Fig. 18c), and siRNA-mediated *Gpr119* knockdown abrogated 2-OG-mediated activation of RAW264.7 cells (Supplementary Fig. 18d). In vivo studies indicated that depletion of liver resident MΦs (Supplementary Fig. 19) or knockdown of *Gpr119* in MΦs (Supplementary Fig. 20) compromised 2-OG-mediated activation of HSCs. Furthermore, antibody-mediated blockade of TGF-β1, but not IL-1 and TNF-α, abrogated 2-OG-enhanced production of ECM genes in the co-cultured HSCs and RAW264.7 cells (Fig. 7c). Molecular analysis indicated that CL-HFS consumption induced significant upregulation of hepatic MΦ gene expression of *Gpr119*, *Tak1*, *Nfkb*, and *Tgfb1*, but not other downstream targets including *Erk1*, *Ampk*, *Jnk*, and *Mapk14* (Supplementary Fig. 21). TAK1 upregulation was also associated with 2-OG mediated activation of RAW264.7 cells and co-cultured HSCs (Supplementary Fig. 22). In vitro studies demonstrated that knockdown of *Tak1* in RAW264.7 compromised the 2-OG-induced upregulation of *Tgfb1* and *Nfkb*, but not *Grp119* (Fig. 7d, e), suggesting NF-κB and TGF-β1 are downstream targets of TAK1. Together, these results suggest that GPR119/TAK1/NF-κB/TGF-β1 signaling pathway mediates 2-OG-induced MΦ activation in HFS-induced NASH liver, and the resultant TGF-β1 acts as a widely recognized master regulator to activate HSC (Fig. 8).

## Discussion

There has recently been a dramatic rise in interest in NAFLD and NASH due to their prevalence and impact on public health^13^. However, our understanding of the interdependence among diet, gut microbiome, and host metabolism is still limited, which significantly impedes the identification of microbial and metabolic targets for NASH therapy^30,31^. By feeding a western-type CL-HFS, we established a murine NASH model which recapitulates the typical features of human disease. Using this model and human patient biospecimens, we have identified *B. producta* and its metabolite 2-OG as a pathogenic bacterium and a metabolic mediator promoting the onset and development of NAFLD/NASH. Mechanistic studies suggest that 2-OG activates HSCs to induce hepatic fibrosis by modulating MΦs through the GPR119/TAK1/NF-κB/TGF-β1 signaling pathway.

Clinical and preclinical studies underscore microbiome-associated mechanisms in NAFLD/NASH^32–34^. However, no microbe-targeted therapeutic approach has been developed to treat NAFLD and NASH^35^. More effort is needed to identify beneficial and pathogenic bacteria and dissect the cellular and molecular mechanisms underlying this increasingly prevalent disease. Our studies identify *B. producta* as a clinically relevant NASH-promoting bacterium. *Blautia*, a genus of *Lachnospiraceae* family^36^, has been reported to increase in human patients with NAFLD^23^ and decrease after NAFLD treatment in rodents^37^. In contrast, other studies showed that there was a negative correlation between *Blautia* and visceral fat^38^ and inflammatory disease^39^, and depletion of *Blautia luti* was related to the worsening of insulin resistance in children with obesity^40^. Here, we showed the abundance of *Blautia* in mice (Fig. 3a, c) and the increase of its family *Lachnospiraceae* in humans (Supplementary Fig. 7) during NASH development. We demonstrated that *Blautia* reduction is associated with antibiotic-mediated suppression of NASH pathogenesis (Figs. 3c and 2). In particular, repopulation with *Blautia* significantly promotes hepatic inflammation and fibrosis in WT mice and further progression of NASH in CL-HFS-fed mice (Fig. 5 and Supplementary Fig. 10). Similarly, supplementation of *B. producta* promotes liver fibrosis in ND-fed mice (Supplementary Fig. 11). However, the impact of *Lachnospiraceae* containing the genus *Blautia* on the host physiology is often inconsistent across different studies^41^. These results suggest a need to profile microbial communities in human NASH patients and clarify their beneficial and pathogenic effects at the species level.

Microbial metabolites play a vital role in host metabolism and human health^42^. Short-chain fatty acids, trimethylamine-N-oxide, bile acids, endogenous ethanol, indole, etc are reported as critical regulators that significantly impact NAFLD/NASH development^43^. Of relevance to our findings, some studies demonstrate that *B. producta* can digest fructose and sucrose to produce acetate, ethanol, lactate, and succinate in association with NASH development^36,44^. In contrast, *Blautia faecis-*producing butyrate was decreased in children with obesity^40,45^. Here, we detected an average increase of ethanol and lactate, but not acetate, butyrate, and succinate, in CL-HFS-fed mice in parallel with *Blautia* abundance, and these increases were not statistically significant (Supplementary Fig. 6). Interestingly, we identified 2-OG as a previsouly unrecognized metabolic mediator causing CL-HFS-induced, but not CCl_4_-induced (Supplementary Fig. 23) mouse liver fibrosis. Of particular clinical relevance, increased 2-OG is detected in both murine and human NASH (Fig. 3d and Fig. 4b). Furthermore, 2-OG reduction is associated with ABX-mediated suppression of gut microbiota that preventively slows NASH onset (Fig. 2). Our in vitro and in vivo studies demonstrate that *B. producta* is a species of lipase-producing bacteria that can produce 2-OG, and *B. producta* repopulation leads to 2-OG accumulation in the liver (Supplementary Fig. 9).

Consistent with our findings, some bioactive lipids, such as arachidonic acids (AA), have been shown to induce inflammatory responses^46,47^. Prostaglandins (PGs) generated from AAs can cause chronic inflammation in several diseases with different cellular and molecular mechanisms, such as activation of immune cells (e.g., MΦs and T helper cells) and amplification of inflammatory responses mediated by pathogen- and damage-associated molecular patterns (PAMPs and DAMPs)^48^. In contrast, some bioactive lipids such as N-acylethanolamines (NAEs), including N-oleoyelthanolamine (OEA) and N-palmitoylethanolamine (PEA), N-linoleylethanolamine (LEA), etc, have been reported to have anti-inflammatory effects^49,50^. Leafort et al. reported that genetic knockout of N-acylphosphatidylethanolamine-selective phospholipase D (NAPE-PLD) in hepatocytes resulted in reduced production of NAEs and 2-OG as well as increased production of AAs, which is associated with the induction of hepatic steatosis^49^. Chen et al. reported that mice receiving NAPE-overexpressing *Escherichia coli* by oral gavage reduced liver triglyceride accumulation and liver inflammation^50^. Studies reported that 2-OG stimulates glucagon-like peptide-1 (GLP-1) and incretin release as an agonist of GPR119 in cells in the human intestine such as intestinal L-cells, resulting in an increase in insulin secretion and improving glucose tolerance^29,51,52^. Together, these studies suggest that the impact of different bioactive lipids on the inflammatory response is associated with specific cell types and environments.

One important finding is that MΦ/HSC crosstalk is a cellular mechanism mediating CL-HFS-induced NASH. In this study, we further demonstrated that 2-OG is a metabolic regulator that can activate HSCs (Supplementary Fig. 15 and 19) and induce liver fibrosis in mice (Fig. 6) in an MΦ-dependent manner. In MΦs, 2-OG regulates the production of TGF-β1 (Fig. 7b, c), a master regulator mediating HSC activation and production of ECM proteins. Activating HSC from a quiescent state into proliferative and fibrogenic myofibroblasts is an essential cellular event of liver fibrosis in both animal models and human patients^53,54^. MΦs contribute to hepatic inflammation and fibrogenesis^55,56^ with 2-OG functioning as an important regulator. These results establish a mechanistic link between hepatic MΦs and HSCs in NASH.

At the molecular level, we demonstrated that 2-OG functions as a GPR119 agonist^29^ to activate MΦs. Through in vivo and in vitro studies, we demonstrated that 2-OG stimulation increased GPR119 production in murine livers (Fig. 6i) and GPR119 expression in MΦs (Fig. 7b). In vivo knockdown of GPR119 in MΦs abrogated 2-OG-induced activation of HSCs (Supplementary Fig. 20). In addition, by comprehensively exploring the canonical signaling pathways responsible for the production of inflammatory cytokine genes including *Nfkb*, *Erk1*, *Ampk*, *Jun*, *Mapk14*, and *Tak1*, we demonstrate that CL-HFS consumption selectively increases expression of *Tak1* in liver MΦs (Supplementary Fig. 21), suggesting TAK1 is involved in CL-HFS-induced hepatic inflammation. Furthermore, *Tak1* knockdown did not influence 2-OG-induced GPR119 expression in MΦs but did block 2-OG-stimulated TGF-β1 and NF-κB expression (Fig. 7). Together, these results suggest that 2-OG activates MΦs through a GRP119/TAK1/NF-κB/TGF-β1 signaling pathway. Studies from others have shown that TAK1 is an essential and positive regulator that impacts innate immune signaling and apoptosis in mouse embryonic fibroblasts, T cells, and other cells, but is a negative regulator that impacts cell development and activation of proinflammatory signaling pathways in neutrophils^57–59^. Further effort is required to investigate whether TAK1 is necessary to mediate 2-OG or CL-HFS-induced NASH in vivo and the role of TAK1 in different subsets of liver cells.

Given NAFLD prevalence is higher in men than in women at all ages^60^, we first selected male mice, but not female mice, in the present studies; therefore, the generalizability of the findings to female mice is unknown.

In conclusion, we have developed a clinically relevant murine NASH model with a typical Western-type diet to enable the study of NASH pathogenesis. *B. producta* and 2-OG are identified as NASH-associated bacteria and metabolic regulators, modulating MΦs through GPR119 signaling. These cellular and molecular mechanistic findings significantly advance our understanding of the diet/gut/liver/immune axis in NASH pathogenesis, which in turn will advance the development of dietary and microbial interventions for this global health threat.

## Methods

### Study approval

For hepatic metabolomics assay, liver specimens with deidentified information were collected from eight human patients with obesity at the University of Missouri Hospital and frozen at −80 °C. Four out of the patients had histologically confirmed NASH and four without NASH. Details are provided in Supplementary Table 3. The study has been approved by the Institutional Review Board (IRB) of the University of Missouri (IRB# 2008258). Written informed consent was obtained from all the study participants. For characterizing human NASH, fourteen slides of formalin-fixed paraffin-embedded (FFPE) human liver biopsies with relevant RNA samples were provided by the University of Kansas Liver Center Biorepository with ethical approval by the University of Kansas Medical Center Institutional Review Board (IRB# 11378) and written informed consent from patients. Seven out of the specimens are from healthy donors and another seven specimens are from patients with histologically confirmed NASH.

Animal protocol (Number 24640) was approved by the University of Missouri-Columbia Animal Care and Use Committee. Animal studies complied with all relevant ethical regulations for vertebrate animal research.

### Animals, diets, and murine NASH model

Wild-type male C57BL/6 J mice (Strain #000664) were purchased from Jackson Laboratory (Bar Harbor, ME). All mice were maintained within a specific-pathogen-free facility on a 12 h light-dark cycle, at temperatures of 65–75 °F (~18–23 °C) with 40–60% humidity. Mice are housed in up to 5 per cage with appropriate bedding and free access to food and water. Mice were monitored for at least 1 week after arrival at the facility before experiments.

Six-week-old C57BL/6 J mice were randomly grouped and fed with ND, CL-HFS, or LFD at the indicated times and experiments, five mice per group. CL-HFS with 40 kcal% fat, 20 kcal% fructose, 2% cholesterol, and 0.05% choline purchased from Research Diets, Inc. (D19061310i, New Brunswick, NJ) was used to induce the mouse NASH model. Normal diet (ND) with 13 kcal% fat, 62.14 kcal% of carbohydrates, 0.014% cholesterol, and 0.2% choline purchased from Lab Supply, Inc. (LabDiet 5053, Fort Worth, TX) was a control diet. A low-fat diet (LFD) with 5 kcal% fat was purchased from Research Diets, Inc. (D07042710i, New Brunswick, NJ) was fed for four weeks to modulate the gut microbiota profile.

### Cell lines, bacterial strains, and medium

Mouse hepatic stellate cell line mHSC^61^ is a generous gift from Dr. Scott L. Friedman of the Mount Sinai School of Medicine (NY, USA), which was cultured in Prigrow III medium (Applied Biological Materials Inc., Richmond, BC). Mouse MΦ cell line RAW264.7 (Catalog #TIB-71, ATCC, Manassas, VA, USA) was cultured in Dulbecco’s Modified Eagle’s Medium (DMEM). Both lines of cells were cultured in a medium supplemented with 10% fetal bovine serum (FBS), 100 U/mL penicillin, and 100 μg/mL streptomycin at 37 °C in a humidified atmosphere with 5% of CO_2_. DMEM, FBS, and penicillin/streptomycin antibiotic solutions were purchased from Thermo Fisher Scientific (Waltham, MA).

Bacterial strains, *Blautia producta* (*B. producta*) (ATCC 27340) and *Alistipes putredinis* (*A. putredinis*) (ATCC 29800), were purchased from ATCC (Manassas, VA). Both bacteria were cultured on Columbia blood agar plates (Thermo Fisher Scientific, Waltham, MA) at 37 °C under anaerobic conditions.

### 16 S ribosomal RNA (rRNA) gene library preparation and sequencing

Stool samples from the distal rectum were aseptically collected from mice and immediately frozen at −80 °C. DNA was isolated with PowerSoil DNA Isolation Kit (MO BIO Labs, Carlsbad, CA) following the manufacturer’s instructions. 16 S rRNA gene library preparation, sequencing, and informatics analysis were conducted at the DNA core facility and Metagenomics Center of the University of Missouri.

Briefly, bacterial 16 S rRNA gene amplicons were constructed via amplification of V4 region of 16 S rRNA gene with universal primers (U515F/806 R) available at proBase. The primers are flanked by Illumina standard adapter sequences. Dual-indexed forward and reverse primers were used in all reactions. PCR was performed in 50 µL reactions containing 100 ng metagenomic DNA, primers (0.2 µM each), dNTPs (200 µM each), and Phusion high-fidelity DNA polymerase (1U, Thermo Fisher). Amplification parameters were 98 °C (3 min) + [98 °C (15 sec) +50 °C (30 sec) +72 °C (30 sec)] ×25 cycles+72 °C (7 min). Amplicon pools (5 µL/reaction) were combined, thoroughly mixed, and then purified by addition of Axygen Axyprep MagPCR clean-up beads to an equal volume of 50 µL of amplicons and incubated for 15 min at room temperature. Products were then washed multiple times with 80% ethanol and the dried pellet was resuspended in 32.5 µL EB buffer (Qiagen), incubated for two minutes at room temperature, and then placed on a magnetic stand for five minutes. The final amplicon pool was evaluated using the Advanced Analytical Fragment Analyzer automated electrophoresis system, quantified using Quant-iT HS dsDNA reagent kits, and diluted according to Illumina’s standard protocol for sequencing on the MiSeq instrument.

DNA sequences were assembled and annotated at the MU Informatics Research Core Facility. Primers were designed to match 5’ ends of forward and reverse reads. Cutadapt (version 2.6; https://github.com/marcelm/cutadapt) was used to remove the primer from the 5’ end of the forward and reverse read. Read pairs were rejected if either read did not match the 5’ primer, with an allowable error rate of 0.1. Two passes were made over each read to ensure the removal of the second primer. A minimal overlap of three bp with the 3’ end of the primer sequence was required for removal.

The QIIME2 DADA2 plugin (version 1.10.0) was used to denoise, de-replicate, and count ASVs (amplicon sequence variants), incorporating the following parameters: 1) forward and reverse reads were truncated to 150 bases, 2) forward and reverse reads with the number of expected errors higher than 2.0 were discarded, and 3) Chimeras were detected using the “consensus” method and removed. R version 3.5.1 and Biom version 2.1.7 were used in QIIME2. Taxonomies were assigned to final sequences using the Silva.v132 database, using a classify-sklearn procedure.

### Antibiotic cocktail treatment of mice

Antibiotic cocktail ABX consists of vancomycin (0.5 g/L), neomycin (0.5 g/L), and imipenem (0.5 g/L), and has no recognized hepatotoxicity^18,19^. Mice received ABX treatment to induce relatively selective suppression of gut microbiota. Antibiotic cocktail ABX5, consisting of ampicillin (100 mg/kg), vancomycin (50 mg/kg), metronidazole (100 mg/kg), neomycin (100 mg/kg), and antifungal amphotericin B (1 mg/kg)^27^, has minimal effect on morbidity and mortality of mice. ABX5 is used to induce broad depletion of microbiota to sterilize the mouse gut for subsequent repopulation with the indicated individual bacterial species to study their effect on NASH.

For ABX and ABX5 treatment, the mice were given ABX or ABX5 in drinking water at the indicated concentrations. Antibiotic water was replaced every other day. With the exception of antifungal amphotericin B from Fisher Scientific (Pittsburgh, PA), antibiotics were from Gold Biotechnology (St. Louis, MO).

### Blood biochemistry test

Blood biochemical profile analysis was used to measure the concentrations of aspartate transaminase (AST), alanine transaminase (ALT), cholesterol, and triglyceride with Olympus 400AUe Chemistry Analyzer (Olympus Corporation, PA) in the Veterinary Medical Diagnostic Laboratory at the University of Missouri. The results were used to assess liver function and lipid metabolism.

### Gut microbiota sterilization and bacterial repopulation

Mice received ABX5 treatment as described above to sterilize the gut, followed by repopulation with B. producta or A. putredinis by oral gavage twice a week for 6 weeks at a dose of 3 × 10^8^ CFU/mouse in 200 µL of PBS.

### Total RNA extraction and real-time PCR (qPCR)

Total RNAs were extracted with RNeasy^@^ Micro Kit (Qiagen, Germantown, MD) according to the manufacturer’s instructions. Reverse transcription of total RNA to cDNA was conducted with a High-Capacity cDNA Reverse Transcription Kit (Applied Biosystems, Foster, CA). qPCR was performed with QuantStudio 3 Detection System (Thermo Fisher Scientific, Waltham, MA) in a 20 μL reaction mixture containing SYBR Green PCR Master Mix (Thermo Fisher Scientific, Waltham, MA). Reactions were run in triplicate. The expression of different genes was normalized to the geometric mean of housekeeping gene 18 S RNA to control for the variability of expression levels. The data were analyzed using the 2^*−ΔΔCT*^ method. All primers were synthesized by Integrated DNA Technologies, Inc. (Skokie, Illinois) and their sequences are given in Supplementary Table 4.

### Isolation of liver non-parenchymal cells (NPCs)

The isolation of liver non-parenchymal cells (NPCs) or leukocytes has been described in our previous publication^62^. Briefly, the livers of anesthetized mice were perfused via the portal vein with 0.05% collagenase (Gibco, Gaithersburg, MD) in Ca^2+^-free PBS at a pump speed of 4 mL/min. Liver tissues were then harvested, cut into small pieces, and incubated in 0.04% collagenase in GBSS (Sigma, St. Louis, MO) at room temperature with continuous shaking at 240 rpm for 20 min. The resulting suspended samples were filtered through 250 µm mesh, then centrifuged at 350 *g* for 10 min at room temperature to pellet cells. The cell pellets were suspended and washed with GBSS (Sigma, St. Louis, MO). After centrifugation, the harvested cells were suspended in 15 mL of GBSS and mixed with 18.45 mL of 30% Nycodenz solution (Accurate Chemical & Scientific Inc., Westbury, NY). The cell suspension underwent gradient centrifugation at 1400 *g* for 20 min at room temperature without a brake. The enriched liver NPCs in the top layer were harvested and washed with PBS, then suspended in culture medium or flow cytometry buffer for the following experiments.

### Flow cytometry

Ex vivo staining of lymphocytes with fluorochrome-labeled antibodies was performed on single-cell suspensions as described^62^. For intracellular staining, the cells were fixed and permeabilized with buffer (Thermo Fisher Scientific, Waltham, MA) and stained with fluorochrome-conjugated antibodies. Stained cells were analyzed with a FACScan Flow Cytometer (BD Biosciences, San Jose, CA). Data were analyzed using FlowJo software version 10.7.1 (Tree Star, Ashland, OR). The sources of all antibodies used in this experiment are listed in Supplementary Table 5.

### H&E staining

Four-μm tissue sections were cut from FFPE liver blocks. Sections were stained with hematoxylin and eosin (H&E) by the Veterinary Medical Diagnostic Laboratory at the University of Missouri. Images were taken with a light microscope (Keyence BZ-X810, Itasca, IL). The inflammatory cells, such as lymphocytes, monocytes, and MΦs, in each tissue section, were counted by pathologists.

### Liver collagen staining and semi-quantification

Collagen staining with Sirius red kit (Catalog #9046, Chondrex, Redmond, WA) was performed according to the manufacturer’s instruction. The red stained areas indicate collagen deposits. Semi-quantification of collagen production was measured by calculating the percentage of the red-stained area of each slide using ImageJ software (National Institutes of Health/NIH, Bethesda, MD). Briefly, the image was scaled and converted into a grayscale during image analysis, then the percentage of the red-stained area was detected. The detailed procedure is described on the NIH website (https://imagej.nih.gov/ij/docs/examples/stained-sections/index.html). Five fields (200×) were randomly selected for each tissue section to calculate the average percentage of the red-stained area for each sample. Five liver tissue sections from different mice in each group were applied for semi-quantification of collagen production.

### Lipid staining and semi-quantification

Frozen liver blocks were used to make 10 μm tissue sections. Lipids staining with Oil Red O Stain Kit (Catalog #ab150678, Abcam, Waltham, MA) was performed according to the manufacturer’s instructions. The tissue section was then stained with hematoxylin for 2 min to show nuclei, rinsed with distilled water, and mounted with an aqueous mounting medium. Images were taken with the microscope (Keyence BZ-X810, Itasca, IL) and evaluated using the ImageJ software.

### Immunohistochemical staining (IHC) and semi-quantification

Four μm tissue sections prepared as described for collagen staining were used for IHC. Briefly, tissue sections were de-paraffinized with xylene, rehydrated with various grades of ethanol (100%, 95%, 80%, and 70%), antigen unmasked with solution (Vector Laboratories Inc., Burlingame, CA), permeabilized with 0.2% Triton X-100, blocked with serum, then incubated with BLOXALL reagent (Vector Laboratories Inc., Burlingame, CA) to quench endogenous peroxidases. Subsequently, the sections were incubated in succession with primary antibodies (Supplementary Table 5) at optimized concentration, secondary antibodies, and DAB substrate to develop color. Semi-quantification was conducted with a method like that of Sirius red staining, and the percentage of α-SMA stained area was measured by ImageJ software.

### Transmission electron microscopy (TEM)

Fresh liver tissues were immediately fixed in Karnovsky’s reagent (2% paraformaldehyde, 2.5% glutaraldehyde in 0.1 M sodium cacodylate buffer, pH 7.4)^63^ and processed at the University of Missouri EM core facility. Observations were made under a JEOL JEM-1400 transmission electron microscope (Japan).

### NAFLD activity score (NAS) calculation

NAS^64^, a widely used measure of grading for steatosis, lobular inflammation, and ballooning, ranging from 0-8, was calculated for each sample. Briefly, NAS is the unweighted sum scores of steatosis (0: <5%, 1: 5–33%, 2: 34–66%, 3: >66% fat accumulation); lobular inflammation (0: no foci inflammation in ×200 field, 1: <2 foci, 2: 2-4 foci, 3: >4 foci); hepatocellular ballooning (0: none, 1: few ballooning cells, 2: prominent ballooning). NAS > 4 was diagnosed with NASH and NAS < 3 was diagnosed as non-NASH.

### siRNA transfection

siRNAs and negative control (NC) were purchased from IDT (catalog #SR411245, OriGene Technologies, Inc., Rockville, MD). To conduct siRNA-mediated knockdown of GPR119 and TAK1 in the mouse RAW264.7 cells, cells were grown to 50% confluence in a 6-well plate, then received 20 nM siRNA transfection with siTran 2.0 siRNA transfection reagent (OriGene Technologies, Inc., Rockville, MD). Eight hours later, the medium was replaced with a complete DMEM medium containing 10% FBS. Cells were cultured for another 48 h for subsequent assays.

### Knockdown of GPR119 in MΦs with shRNA

Mouse Gpr119 shRNA lentiviral particles and scrambled shRNA lentiviral particles were purchased from OriGene Technologies, Inc., (catalog #TL507165V, Rockville, MD) The lentivirus particles were transfected into RAW264.7 cells or bone marrow-derived MΦs grown to 80% confluence. Twelve hours post-infection, the cell medium was changed to the normal culture medium. On day 3, *GPR119* mRNA levels in the transfected cells, designated *GPR119*-knockdown MΦs, were measured to confirm *GPR119* knockdown. Bone marrow-derived MΦs were induced with M-CSF (100 ng/mL, macrophage colony-stimulating factor) as we have done previously^65^.

### 2-Oleoylglycerol treatment

2-OG was purchased from Cayman Chemical Company (catalog #16537, Ann Arbor, MI). To induce liver fibrosis, mice received I.V. administration of 2-OG at a dose of 20 µg/mouse three times a week for 6 weeks. For cell stimulation, mouse macrophage (MΦ) cell line RAW264.7 cells, HSCs, or their coculture received 2-OG at a dose of 50 μg/mL for 24 h.

### Intraperitoneal administration of carbon tetrachloride (CCl_4_)

CCl_4_ solution (10% (v/v)) in corn oil (Thermo Scientific, Waltham, MA) was I.P. injected into WT C57BL/6 J mice twice a week for 6 weeks at 8 mL/kg of body weight to induce liver fibrosis.

### GC-MS sample preparation and non-targeted metabolite analysis

Liver biopsies from each mouse were collected and immediately frozen in liquid nitrogen. We prepared liver samples for metabolomics assays as a previously published protocol^66^ with minor revision. In detail, 50 mg of each liver sample was weighed and added into 0.25 mL prechilled methanol containing 0.1% formic acid (Fisher Scientific, Pittsburgh, PA). Ribitol was chosen as an internal standard in previous studies from others^67,68^ and our current study because it is not an endogenous metabolite in animals, and therefore does not interfere with the analysis. In this analysis, ribitol (Sigma-Aldrich, St. Louis, MO) was spiked into each sample at a concentration of 32.79 µg/mL, followed by a vortex for 20 s and subsequent homogenization with a bead beater at room temperature. The homogenized slurry sample was centrifuged at room temperature at 13,000 *g* for 15 min. After centrifugation, 200 µL of supernatant from each sample was dried under a gentle stream of nitrogen (N2, Airgas, Inc., Columbia, MO) gas at room temperature and stored at −80 °C for derivatization. Metabolite derivatization was performed immediately prior to GC-MS analysis. Each dried extract was methoximated with 50 μL of freshly prepared methoxyamine hydrochloride in pyridine (Sigma-Aldrich, St. Louis) at a concentration of 15 mg/mL at 50 °C for 1 h. Finally, the samples were derivatized with 50 μL MSTFA (N-methyl-N-(trimethyl-silyl) trifluoroacetamide) +1% TMCS (chlorotrimethylsilane) (Thermo Fisher Scientific, Waltham, MA) at 50 °C for 1 h.

GC/MS analysis was performed on an Agilent 6890 gas chromatographer coupled with an Agilent 5973 mass selective detector (MSD) at the Metabolic Center in the University of Missouri. The GC column was an Agilent J&W DB-5MS (Length: 60 m, Inner Diameter: 0.25 mm, Film thickness: 0.25 µm). Helium was used as the carrier gas at a constant flow rate of 1.0 mL/min. Each sample (1 µL) was injected in splitless mode. The oven temperature was initially held at 80 °C for 2 min, then ramped to 315 °C at 5 °C/min and held at 315 °C for 12 min. The transfer line temperature was 280 °C. MS spectra were acquired in scan mode at a frequency of 2.42 spectra per second. The mass range was set as 50-650 m/z.

The raw GC-MS data were deconvoluted and processed using automated mass spectral deconvolution and identification system software (AMDIS, version 2.73). Metabolite identification was performed by spectral matching using an in-house spectral library, GOLM Metabolome Database library, and the National Institute of Standards and Technology (NIST17) mass spectral library. The threshold of spectrum matching was set to >75. Metabolomics Ion-based Data Extraction Algorithm (MET-IDEA, version 2.06) was used for relative quantification of each metabolite by extracting representative ion intensity values from the AMDIS output files (*.ELU) and then normalizing their abundances to that of the internal standard^69^. A representative ion is a “model” ion listed in the *.ELU files. Multivariate analyses were performed using the online software MetaboAnalyst 5.0 (http://www.metaboanalyst.ca). The raw data were deposited in the NIH Common Fund’s National Metabolomics Data Repository (NMDR)^70^.

For quantification of 2-OG concentrations in *B. producta* culture media and post-2-OG treatment in mice, a calibration curve was constructed using authentic standard 2-OG. A linear calibration curve was constructed in the 0.01–1.25 μg/mL range with an R^2^ = 0.9995.

### Statistical analysis

Statistical significance between groups was determined using one-way ANOVA ( ≥ 3 groups) followed by recommended correction with Tukey’s multiple comparison test or one-tailed or two-sided Mann–Whitney test (two groups) with GraphPad Prism software (version 8.3.0, GraphPad Software, La Jolla, CA). Data are represented as mean ± standard deviation (SD). A *p-*value of less than 0.05 was considered a significant difference.

### Reporting summary

Further information on research design is available in the Nature Portfolio Reporting Summary linked to this article.

## Supplementary information

Supplementary information

Reporting Summary

## Data Availability

All the raw data for generating the figures are provided in the Source Data file. The raw 16 S rRNA gene sequencing data of mice fecal samples generated in this study have been deposited into NCBI Sequence Read Archive (SRA) with NCBI BioProject ID: PRJNA719798 and free access link. The raw data for mouse liver metabolites have been deposited in the NIH Common Fund’s National Metabolomics Data Repository (NMDR) with study ID: ST002156. The data can be accessed directly via the project digital object identifier (DOI): 10.21228/M8RQ67 and can be downloaded directly without an access code. Source data are provided with this paper.

## Source data

Source Data
