# Supplementary material for: Western diet contributes to the pathogenesis of non-alcoholic steatohepatitis in male mice via remodeling gut microbiota and increasing production of 2-oleoylglycerol
Source: Nat Commun. 2023 Jan 16;14:228. doi: 10.1038/s41467-023-35861-1 (PMC9842745; doi:10.1038/s41467-023-35861-1)
Supplement: Supplementary file 1 — Supplementary information [file 41467_2023_35861_MOESM1_ESM.pdf]

Supplementary Information for

Western diet contributes to the pathogenesis of non-alcoholic steatohepatitis in male mice via remodeling gut microbiota and increasing production of 2-oleoylglycerol

Yang et al.

This PDF file includes:

Supplementary Fig. 1 to Fig. 23

Supplementary Table 1 to Table 5

Supplementary Figures and Figure Legends

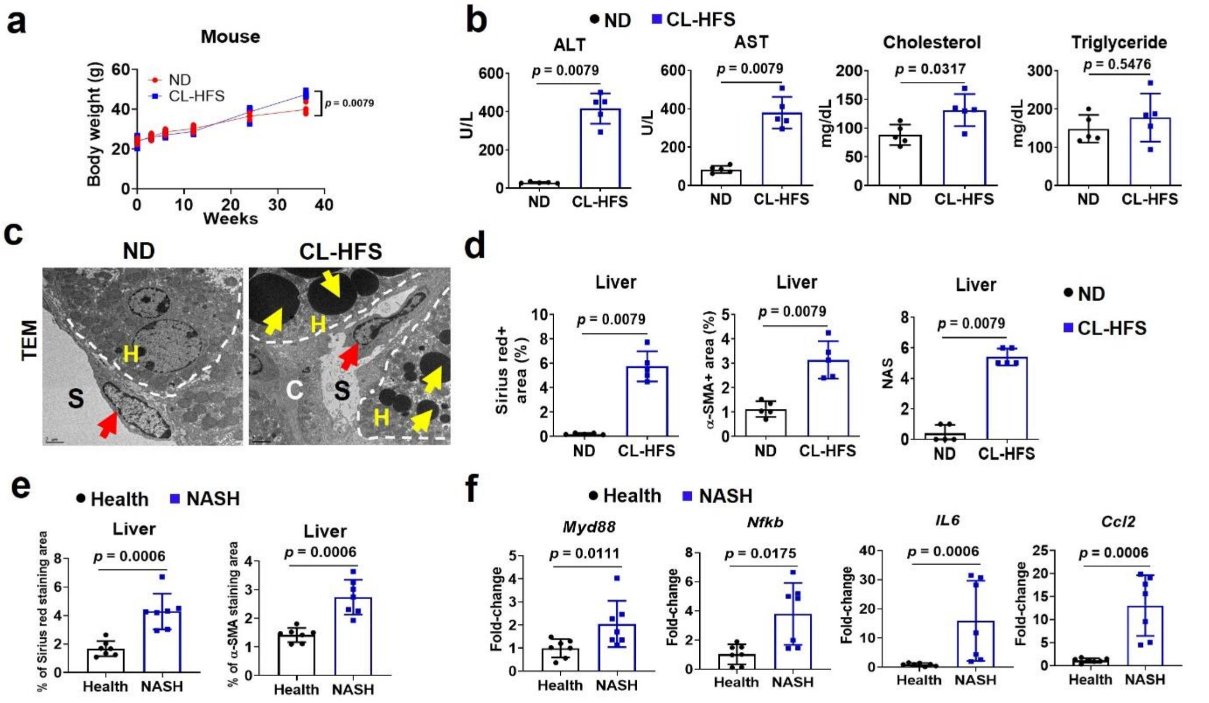

Supplementary Fig. 1. Characterization of a new mouse NASH model induced with a CL-HFS. Six-week-old WT mice were divided into two groups which received either ND or CL-HFS up to 36 weeks. **a** Mean bodyweight. Individual mice were weighed at the indicated time points and the mean body weights of mice in each experimental group were calculated. At week 36, a significantly increased body weight was detected in CL-HFS-fed mice compared to ND-fed mice. **b** Liver injury in ND- and CL-HFS-fed mice. Levels of ALT, AST, cholesterol, and triglyceride in the plasma of ND- and CL-HFS-fed mice were measured after receiving the diet for 12 weeks (Fig. 1a). Significantly increased serum levels of ALT and AST were detected in CL-HFS-fed mice compared to ND-fed mice. For **a**, **b**, and **d**, n=5, error bars represent mean ± SD. **c** TEM imaging. Transmission electron microscope (TEM) imaging showed that CL-HFS induced substantial lipid accumulation in hepatocytes. S: Liver sinusoid; H: Hepatocytes; C: Collagen bundles; Red arrow: liver sinusoid endothelial cells; Yellow arrow: lipid drop; **d** Collagen production, α-SMA production, and NAS in mouse livers. Semi-quantification of hepatic collagens and α-SMA production was generated by measuring the area of positive staining for Sirius Red or IHC, respectively performed as described in Figure 1C. NAS, one grading system for NAFLD, was calculated as described in Methods. n=5, error bars represent mean ± SD. **e** Semi-quantification of collagen production in the livers of human patients with NASH and healthy controls. With the method used in **d**, the areas of positive staining for Sirius Red and IHC in Fig. 1f were quantified. **f** Production of

inflammatory cytokine, chemokine, and signaling molecules. qPCR detected the increased mRNA expression of genes *IL6*, *Ccl2*, *Myd88*, and *Nfkb* in the livers of human patients with NASH compared to healthy individuals. For **e** and **f**, n=7, data are presented as mean ± SD. Statistical analysis of data was performed by Mann–Whitney test (two-tailed). Source data are provided as a Source Data file.

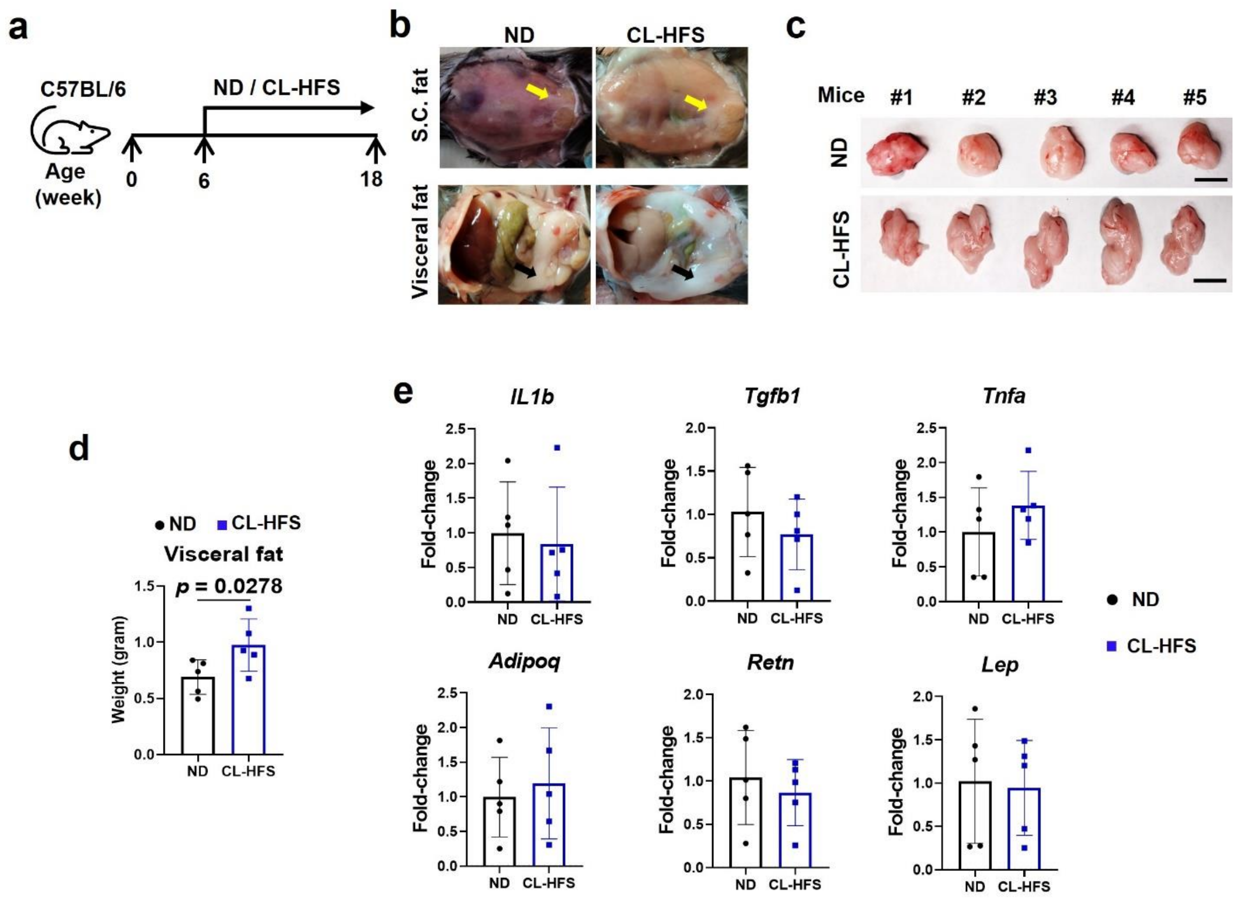

Supplementary Fig. 2. Accumulation of visceral fat in mice with CL-HFS-induced NASH and production of inflammatory cytokines and adipokines in fat. a An outline depicting induction of NASH with a CL-HFS. Six-week-old WT mice were divided into two groups which received either ND or CL-HFS for 12 weeks. **b** Representative macroscopic view of subcutaneous (yellow arrows) and visceral fat accumulation (black arrows). **c** Visceral fat images from ND and CL-HFS treated mice. Bar: 1 cm. **d** Weight of visceral fat from ND and CL-HFS treated mice. Visceral fat was significantly increased in CL-HFS-fed mice compared to ND-fed mice. **e** mRNA expression of proinflammatory cytokines and adipokines. qPCR did not detect any significant differences in the mRNA levels of proinflammatory genes including *Il1b*, *Tgfb1*, and *Tnfa*, and adipokine genes including *Adipoq* (adiponectin), *Retn* (resistin), and *Lep* (leptin) in visceral adipose tissues between the two groups of mice. n=5, data are presented as mean ± SD. Statistical analysis of data was performed by Mann–Whitney test (One-tailed). Source data are provided as a Source Data file.

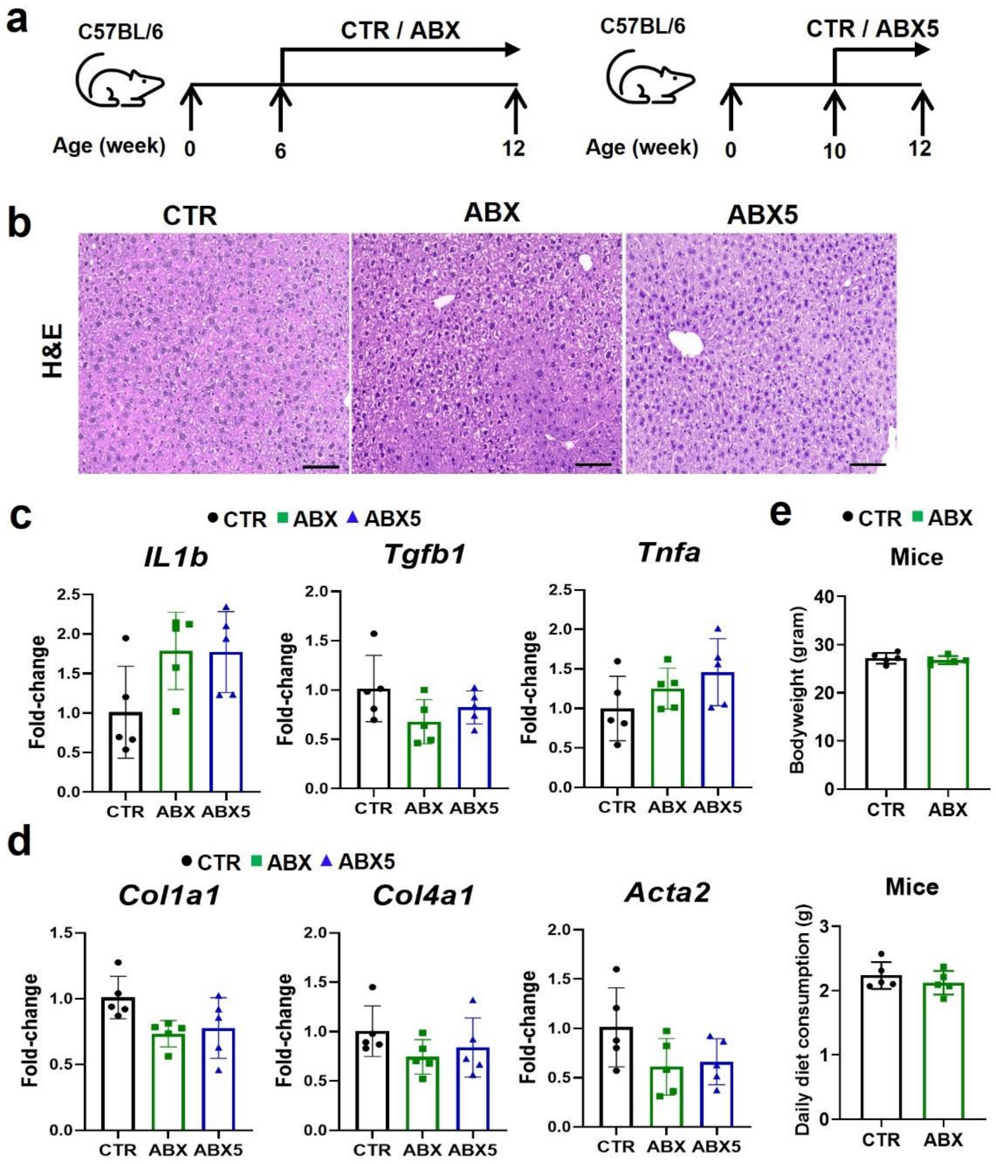

Supplementary Fig. 3. ***In vivo*** **influence of ABX and ABX5 on liver inflammation and fibrosis in WT mice. a** An outline depicting the treatment of mice with ABX or ABX5. Six-week-old WT mice were divided into three groups which received either sterile water, ABX in drinking water for 6 weeks, or ABX5 in drinking water for 2 weeks. **b** Representative microscopic view of H&E staining. Bar: 100 µm. **c** qPCR for detection of mRNA expression of proinflammatory cytokines in liver tissues of mice from each experimental group. **d** qPCR for detection of mRNA expression of extracellular matrix (ECM) genes in liver tissues of mice from each experimental group. qPCR did not detect any significant differences in hepatic mRNA expression of proinflammatory cytokine genes *Il1b*, *Tgfb1*, and *Tnfa*, and ECM genes *Col1a1*, *Col4a1*, and *Acta2* among the three groups of mice. **e** The influence of ABX treatment on mouse body weight and daily food consumption.

n=5, data are presented as mean ± SD. Statistical analysis of data was performed by one-way ANOVA with Tukey's multiple comparison test. Source data are provided as a Source Data file.

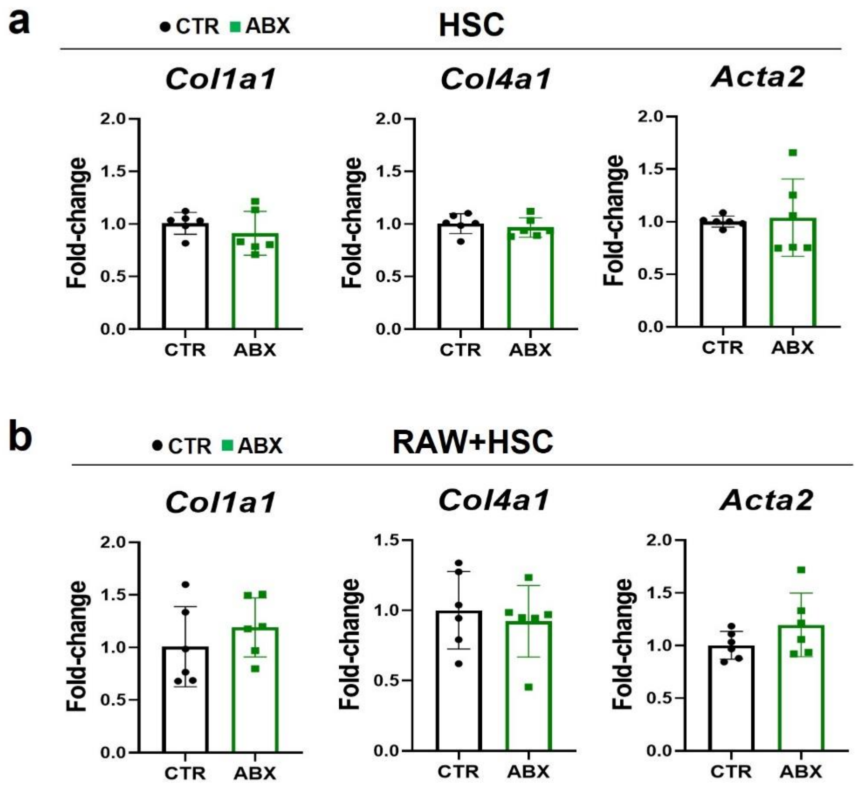

Supplementary Fig. 4. ABX does not suppress ECM gene expression directly. a Influence of ABX on the expression of ECM genes in HSCs. **b** Impact of ABX on the expression of ECM genes in co-cultured HSCs and RAW264.7 cells. qPCR did not detect any significant differences in mRNA expression of ECM genes *Col1a1*, *Col4a1*, and *Acta2* in HSCs or HSCs cocultured cells with RAW264.7 cells with or without ABX treatment for 24 hours. n=6, data are presented as mean ± SD. Statistical analysis of data was performed by Mann–Whitney test (two-tailed). Source data are provided as a Source Data file.

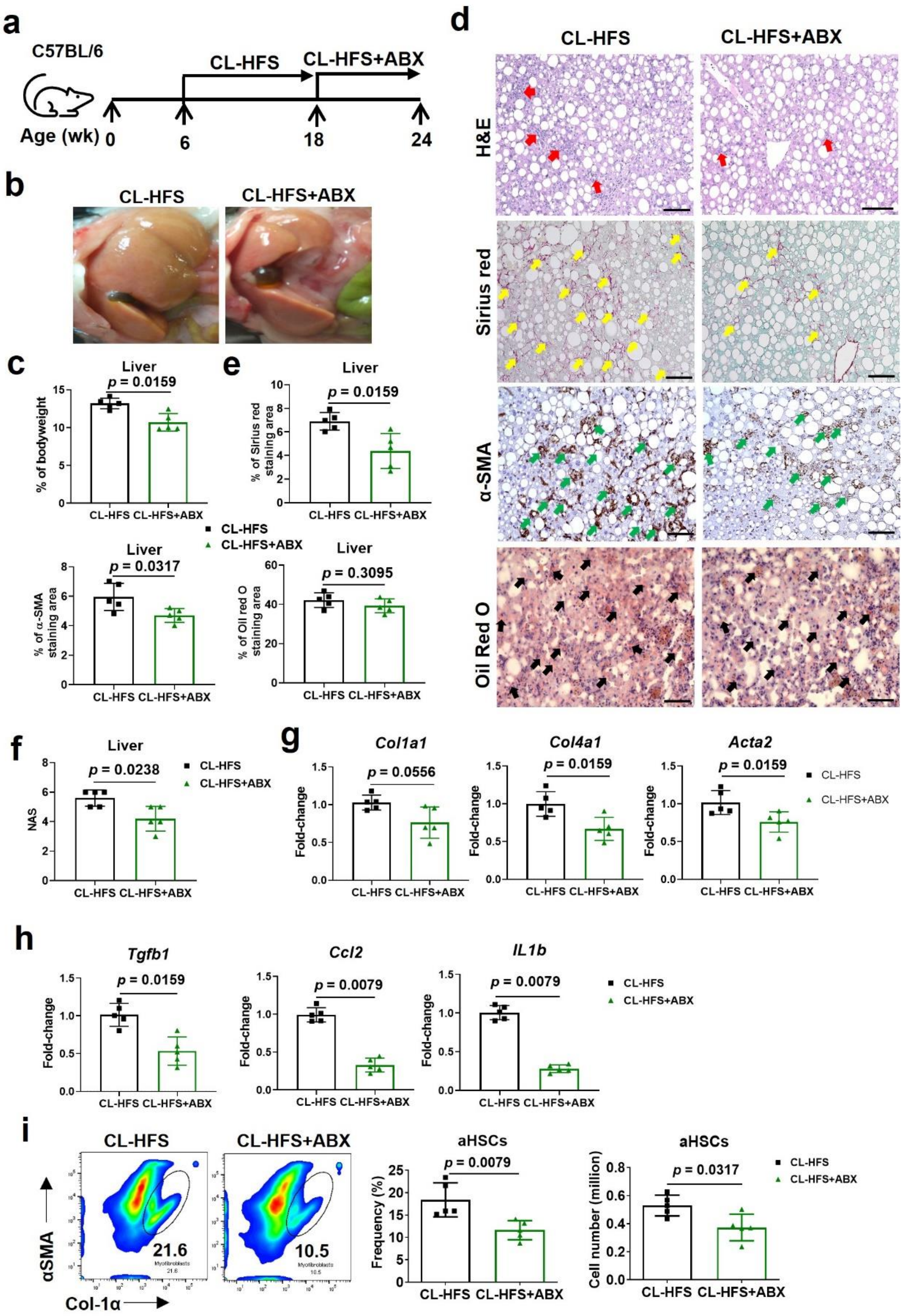

Supplementary Fig. 5. ABX therapeutically suppresses CL-HFS-induced liver fibrosis and inflammation. a An outline depicting ABX treatment of CL-HFS-fed mice. Six-week-old WT mice

received CL-HFS for 12 weeks to induce NASH. After that, the mice were divided into two groups that did or did not receive ABX treatment. Six weeks later, the mice were euthanized for the following studies. **b** Representative macroscopic images of livers in the control and ABX-treated mice. **c** Liver-to-bodyweight ratios. There was a significant reduction in liver-to-bodyweight ratios in ABX-treated mice versus control mice. **d** Inflammatory cell liver infiltration, hepatic collagen production, α-SMA production, and lipid accumulation. ABX treatment significantly reduced liver infiltration of inflammatory cells shown by H&E staining (Red arrows point to inflammatory cells), liver collagen production shown by Sirius red staining (Yellow arrows), and liver α-SMA production detected by IHC staining (Green arrows), but not lipid deposit shown by Oil red O staining (Black arrows). Bar: 100 µm. **e** Semi-quantification of hepatic collagen production, α-SMA production, and lipid accumulation. The cumulative results showed that ABX treatment significantly reduced hepatic collagen and α-SMA production, but only slightly decreased lipid accumulation. **f** NAS of mice. ABX treatment significantly reduced NAS. **g** Effect of ABX on hepatic mRNA expression of ECM genes. qPCR detection showed that ABX treatment significantly reduced hepatic mRNA expression of genes *Col4a1* and *Acta2*, but not *Col1a1* in the mice. **h** Effect of ABX on hepatic mRNA expression of cytokines and chemokines. qPCR detection showed that ABX treatment significantly reduced hepatic mRNA expression of genes *Tgfb1*, *Ccl2*, and *Il1b* in the mice. **i** Effect of ABX on HSC activation. Representative flow cytometric analysis showed that ABX treatment led to an obvious decrease in the frequency of activated HSCs expressing Col1α and α-SMA (Left panel) in the mice. The cumulative results for the mean frequency and cell number of activated HSCs expressing α-SMA and Col1α are shown in the right panel. n=5, data are presented as mean ± SD. Statistical analysis of data was performed by Mann–Whitney test (two-tailed). Source data are provided as a Source Data file.

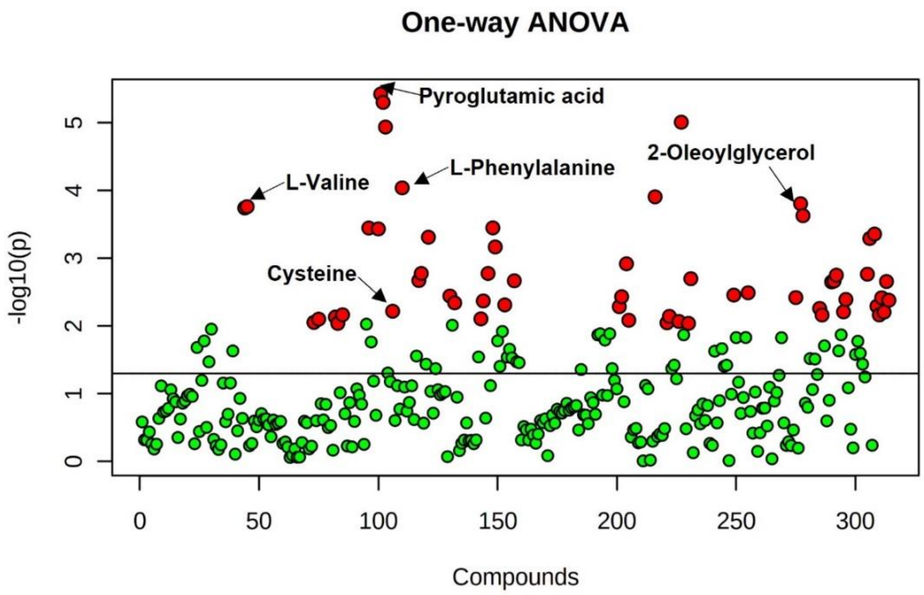

Supplementary Fig. 6. ABX treatment changes the profile of hepatic metabolites. Hepatic metabolites were measured and analyzed in ND-fed mice and CL-HFS-fed mice with or without simultaneous ABX treatment as described in Fig. 2. Non-targeted Gas chromatography-mass spectrometry (GC-MS) was used for metabolic analysis as described in Methods. Each dot represents one metabolite. A total of 309 metabolites were detected. All red dots represent metabolites with a significant difference between ND-fed mice versus CL-HFS-fed mice. Among them, five labeled metabolites in CL-HFS-fed mice were significantly decreased after receiving ABX treatment; other unlabeled red dots represent metabolites that are unknown or did not change significantly with ABX treatment. Statistical analysis of data was performed by one-way ANOVA with Tukey's multiple comparison test with MetaboAnalyst 5.0 (http://www.metaboanalyst.ca). Source data are provided as a Source Data file.

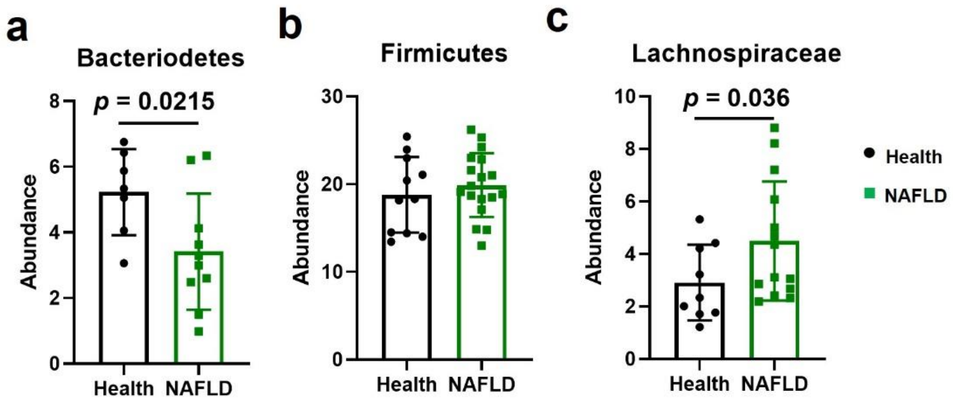

Supplementary Fig. 7. Gut microbiota dysbiosis in human patients with NAFLD. Data on gut microbiota from NAFLD patients and healthy controls were collected from BioProject PRJNA540738 (https://www.ncbi.nlm.nih.gov/bioproject/540738). **a** There was a decreased abundance of Phylum *Bacteriodetes* in human patients with NAFLD compared to healthy individuals. **b** There was no significant difference in Phylum *Firmicutes* between healthy individuals and human patients with NAFLD. **c** There was a significantly increased abundance of family *Lachnospiraceae* in human patients with NAFLD compared to healthy individuals. Genus *Blautia* belongs to this bacterial family. n=7 (healthy controls) and n=18 (human patients with NAFLD), data are presented as mean ± SD. Statistical analysis of data was performed by Mann–Whitney test (One-tailed). Source data are provided as a Source Data file.

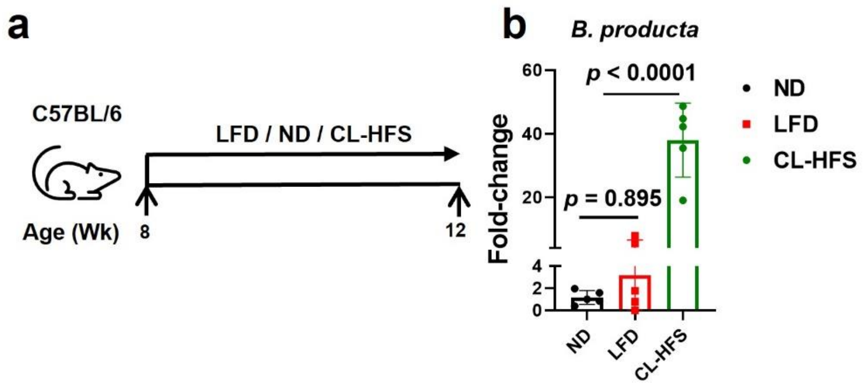

Supplementary Fig. 8. The abundance of *B. producta* in low-fat diet (LFD)-fed mice. a An outline depicting the treatments of mice with ND, LFD, or CL-HFS. Eight-week-old WT mice were fed with either ND, LFD, or CL-HFS for 4 weeks. Feces were collected for the determination of the relative abundance of *B. producta* with qPCR. **b** *B. producta* abundance in mice feces. qPCR detected a significantly lower abundance of *B. producta* DNAs in the feces of LFD-fed mice and ND-fed mice compared to CL-HFS-fed mice. n=5, data are presented as mean ± SD. Statistical analysis of data was performed by one-way ANOVA with Tukey's multiple comparison test. Source data are provided as a Source Data file.

a

Bacteria	2-OG production
Blautia producta	Yes (0.30 μg/mL)
Alistipes putredinis	Non-detectable

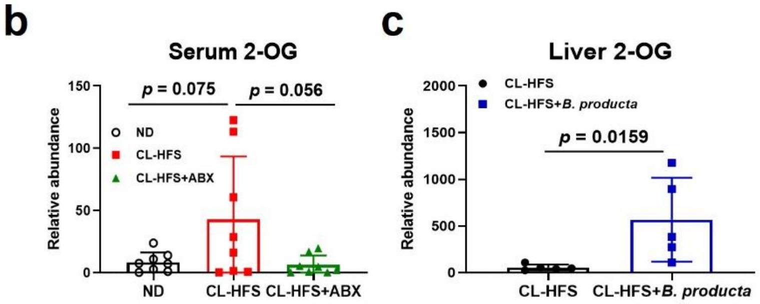

Supplementary Fig. 9. *B. producta* is lipase-producing bacteria producing 2-OG *in vitro* and *in vivo*. a Evaluating the capacity of *B. producta* to produce 2-OG. Two species of bacteria including lipase-producing *Blautia producta* and control *Alistipes putredinis,* which does not have the lipase gene, were grown in a medium containing ND in anaerobic conditions. 72 hours later, 2-OG production in the culture supernatant was measured by GC-MS. n=3, data are presented as mean concentration. **b** Circulating blood 2-OG production in CL-HFS-fed mice with or without ABX treatment. Serum was collected from the mice as in Fig. 2. Analysis of serum 2-OG was performed by GC-MS. n=8, data are presented as mean ± SD. **c** Effect of *B. producta* repopulation on 2-OG production in CL-HFS-fed mice. After receiving CL-HFS for 12 weeks, the mice were sterilized with ABX5 followed by *B. producta* repopulation; 6 weeks later, significantly increased hepatic 2-OG production was detected in the mice compared to control mice without *B. producta* repopulation. n=5, data are presented as mean ± SD. Statistical analysis of data was performed by one-way ANOVA with Tukey’s multiple comparison test for three groups or Mann–Whitney test (two-tailed) for two groups. Source data are provided as a Source Data file.

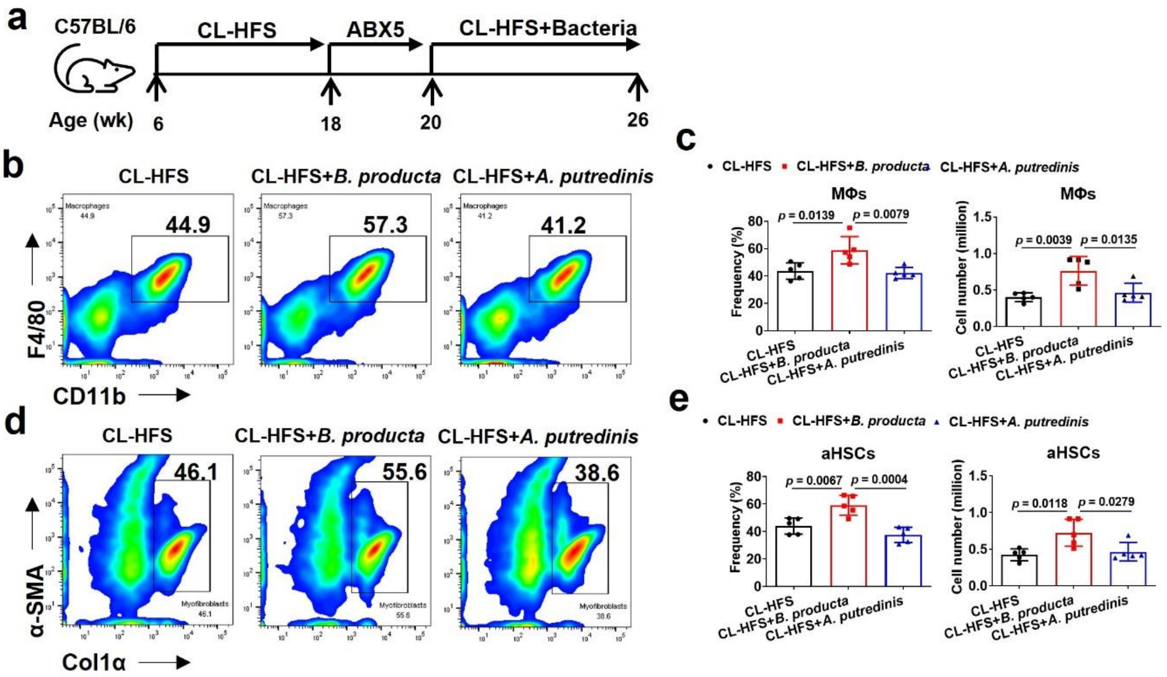

Supplementary Fig. 10. *B. producta* repopulation activates MΦs and HSCs in NASH mice. **a** An outline depicting the experimental design of *B. producta* repopulation. Six-week-old WT mice successively received CL-HFS for 12 weeks to induce NASH, ABX5 treatment for two weeks to sterilize the gut, and oral gavage of *B. producta* (ATCC 27340) or *A. putredinis* (ATCC 29800) for repopulation twice a week at a dose of 3×10^8 CFU/mouse in 200 µL of PBS. Six weeks after the repopulation, the mice in each group were euthanized for the following studies. **b** Representative frequencies of liver resident MΦs in the indicated mice. Representative flow cytometric analysis showed the frequencies of liver resident MΦs positive for CD11b and F4/80 in each experimental group of mice. Repopulation with *B. producta*, but not *A. putredinis*, increased the frequency of liver resident MΦs positive for CD11b and F4/80. **c** Mean frequency and absolute cell number of MΦs in three groups of mice. The accumulated results suggested that *B. producta* repopulation significantly increased the frequency and the absolute number of liver-resident $CD11b^+F4/80^+$MΦs in CL-HFS-fed mice. **d** Representative frequencies of HSCs in each experimental group of mice. Representative flow cytometric analysis showed that repopulation with *B. producta*, but not *A. putredinis*, led to an increase in the frequency of HSCs expressing Col1α and α-SMA. **e** Mean frequency and absolute cell number of HSCs in each experimental group of mice. The cumulative results suggested that *B. producta* repopulation significantly increased the frequency and absolute number of HSCs in CL-HFS-fed mice. n=5, data are presented as mean ± SD. Statistical analysis of data was performed by one-way ANOVA with Tukey's multiple comparison test. Source data are provided as a Source Data file.

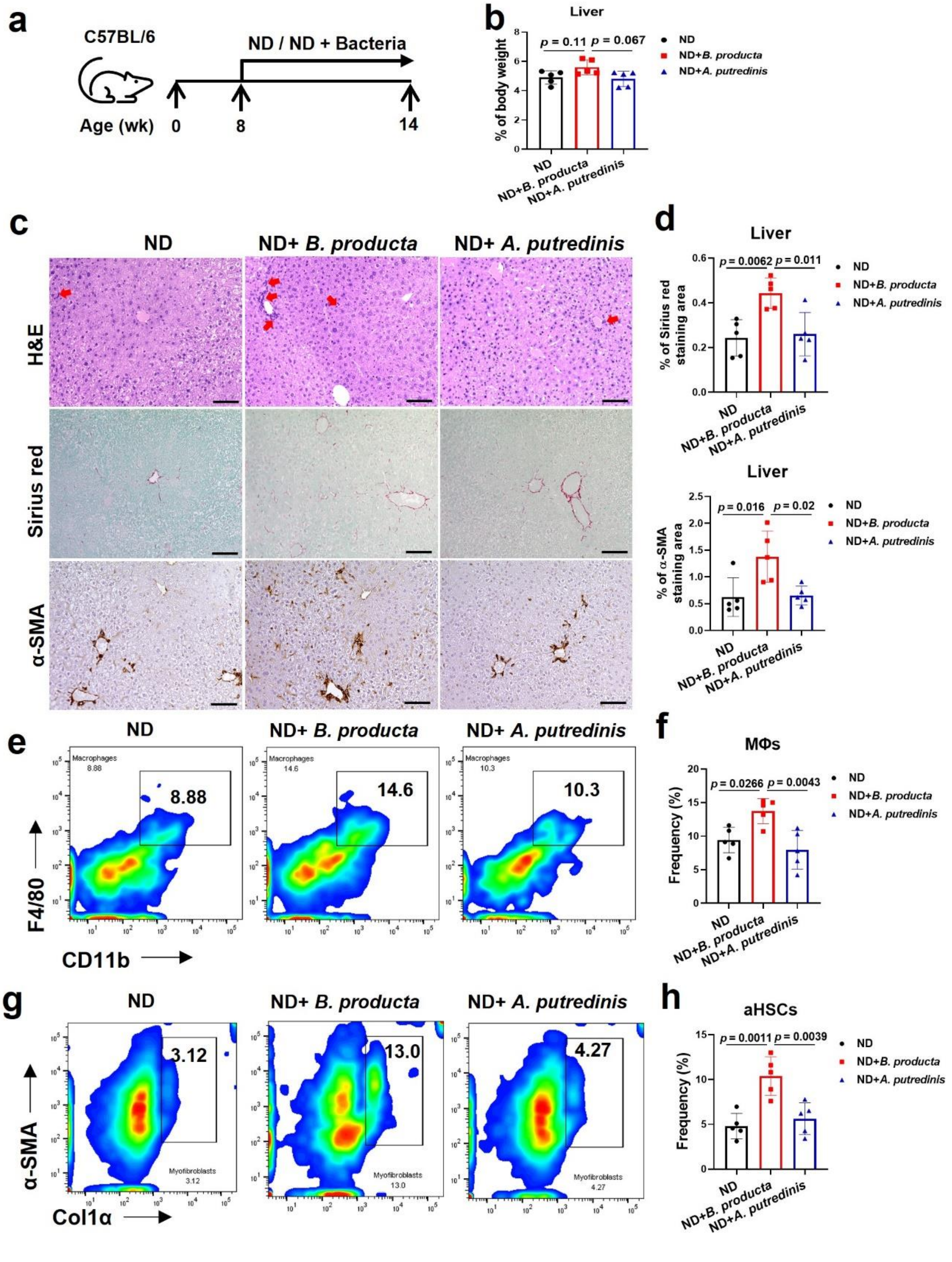

Supplementary Fig. 11. ***B. producta*** **supplementation activates MΦs and HSCs in ND-fed WT mice.** **a** An outline depicting the experimental design of *B. producta* supplementation. Eight-week-old WT mice successively received oral gavage of *B. producta* or *A. putredinis* twice a week for six weeks at a dose of 3×10^{8} CFU/mouse in 200 µL of volume. After that, the mice in each

group were euthanized for the following studies. **b** Liver-to-bodyweight ratio. There is a significant change in liver-to-bodyweight ratios among different treatments. **c** Inflammatory cell liver infiltration and hepatic collagen and α-SMA production. H&E staining showed that *B. producta* supplementation increased the inflammatory cell liver infiltration (Top panel: red arrows point to inflammatory cells). Sirius red staining showed that ABX treatment decreased liver collagen production (Middle panel). IHC staining displayed ABX treatment reduced liver α-SMA production (Low panel). Bar: 100 µm. **d** Semi-quantification of hepatic collagen production and α-SMA production. The cumulative results showed that *B. producta* supplementation significantly increased hepatic collagen and α-SMA production. **e** Representative flow cytometric analysis showed the frequency of liver resident MΦs positive for CD11b and F4/80 in each experimental group of mice. **f** Mean frequency of MΦs in three groups of mice. The cumulative results suggest that *B. producta* supplementation significantly increased the frequency of liver resident $CD11b^+F4/80^+$MΦs in ND-fed mice. **g** Representative frequencies of HSCs in each experimental group of mice. **h** Mean frequency of HSCs in each experimental group of mice. The cumulative results suggest that *B. producta* supplementation significantly increased the frequency of HSCs expressing Col1α and α-SMA in WT mice. n=5, data are presented as mean ± SD. Statistical analysis of data was performed by one-way ANOVA with Tukey's multiple comparison test. Source data are provided as a Source Data file.

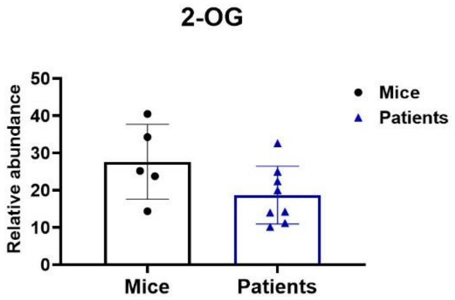

Supplementary Fig. 12. Comparison of hepatic 2-OG levels in 2-OG treated mice and human patients with obesity. Mice received 2-OG administration at a dose of 20 µg/mouse three times a week for 6 weeks as described in Fig. 6a. After that, we harvested livers for GC-MS quantification of 2-OG levels and compared the mouse levels to hepatic 2-OG in the human patients with obesity presented in Fig. 4b. n=5 (mice) and n=8 (patients with obesity), data are presented as mean ± SD ($p > 0.05$). Mann–Whitney test (two-tailed) didn't show a statistically significant difference. Source data are provided as a Source Data file.

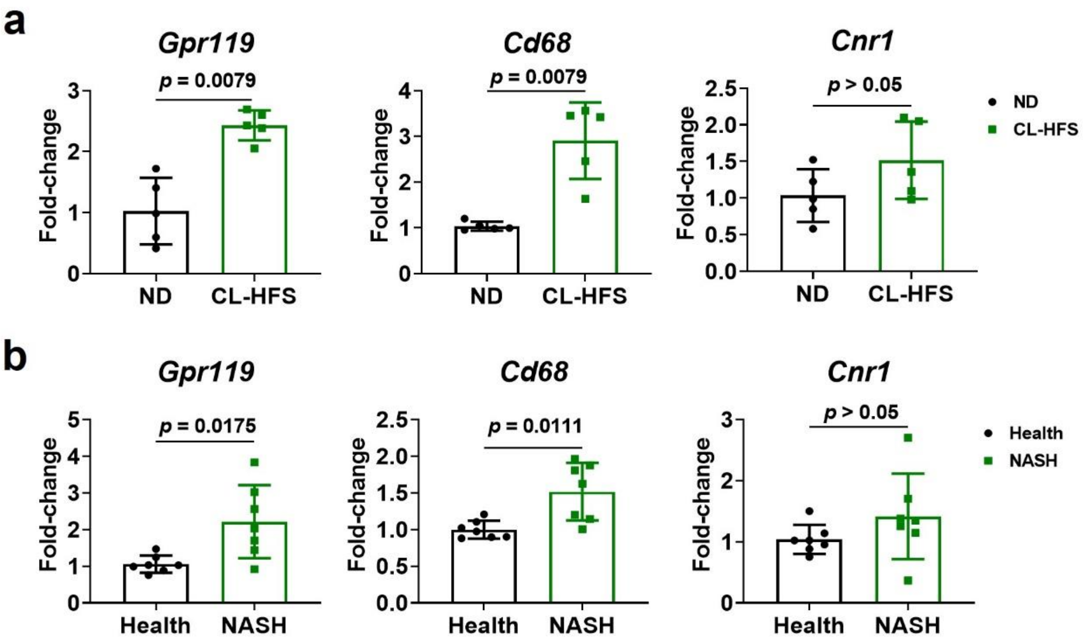

Supplementary Fig. 13. Increased hepatic expression of *Gpr119* and *Cd68* in mice and human patients with NASH. a Expression of hepatic *Grp119* and *Cd68* in mice with CL-HFS-induced NASH. qPCR detected significantly increased mRNA expression of hepatic *Gpr119* and *Cd68* (MΦ marker gene) but not *CNR1* in the mice which received 12 weeks of CL-HFS versus ND. n=5, data are presented as mean ± SD. **b** Expression of hepatic *Grp119* and *Cd68* in healthy subjects and human patients with NASH. qPCR detected significantly increased mRNA expression of hepatic *Gpr119* and *Cd68* but not *Cnr1* in patients with NASH compared to healthy individuals. n=7, data are presented as mean ± SD. Statistical analysis of data was performed by Mann–Whitney test (two-tailed). Source data are provided as a Source Data file.

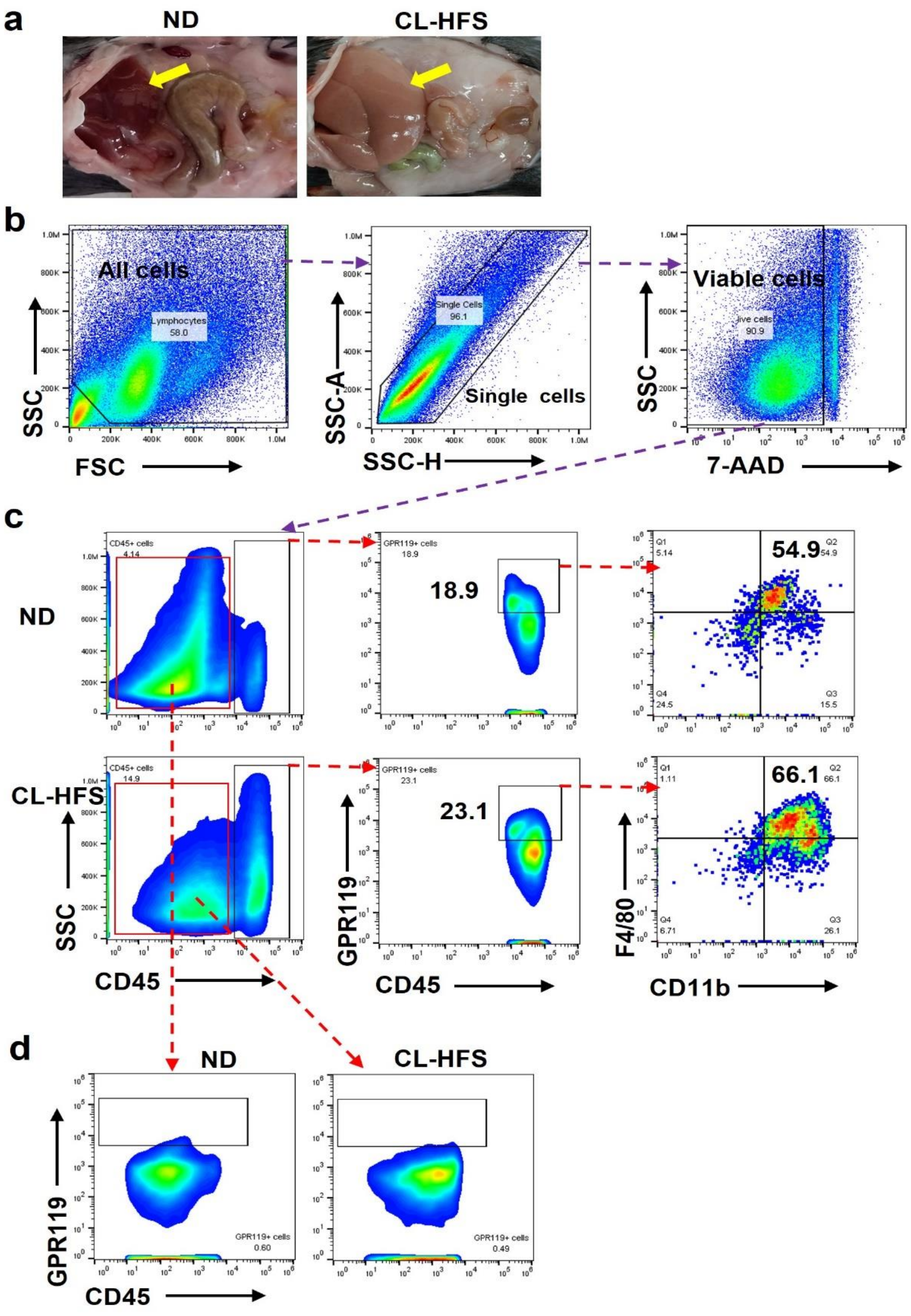

Supplementary Fig. 14. Cell gating and flow cytometric analysis to detect hepatic GPR119 expression in the different kinds of hepatic cells. a Macroscopic images of livers from ND- or CL-HFS-fed mice. Six-week-old WT mice were fed with ND or CL-HFS for 12 weeks, then

euthanized for isolation of liver NPCs. **b** Cell gating and flow cytometric analysis. Representative flow cytometry figures show cell gating for viable hepatic cells. Left: SSC vs FSC for excluding debris; Middle: SSC-A vs SSC-H for gating single cells; Right: SSC vs viable dye 7-AAD for excluding dead cells. **c** GPR119 expression in liver $CD45^+$ cells. SSC vs CD45 for separating leukocytes and hepatocytes. The data showed that the expression of GPR119 and CD45 was mainly seen in hepatic MΦs ($F4/80^+CD11b^+$) in both ND and CL-HFS-fed mice. **d** GPR119 expression in liver-resident $CD45^-$ cells. The data showed that only a small population of hepatocytes express GPR119 in both ND and CL-HFS-fed mice.

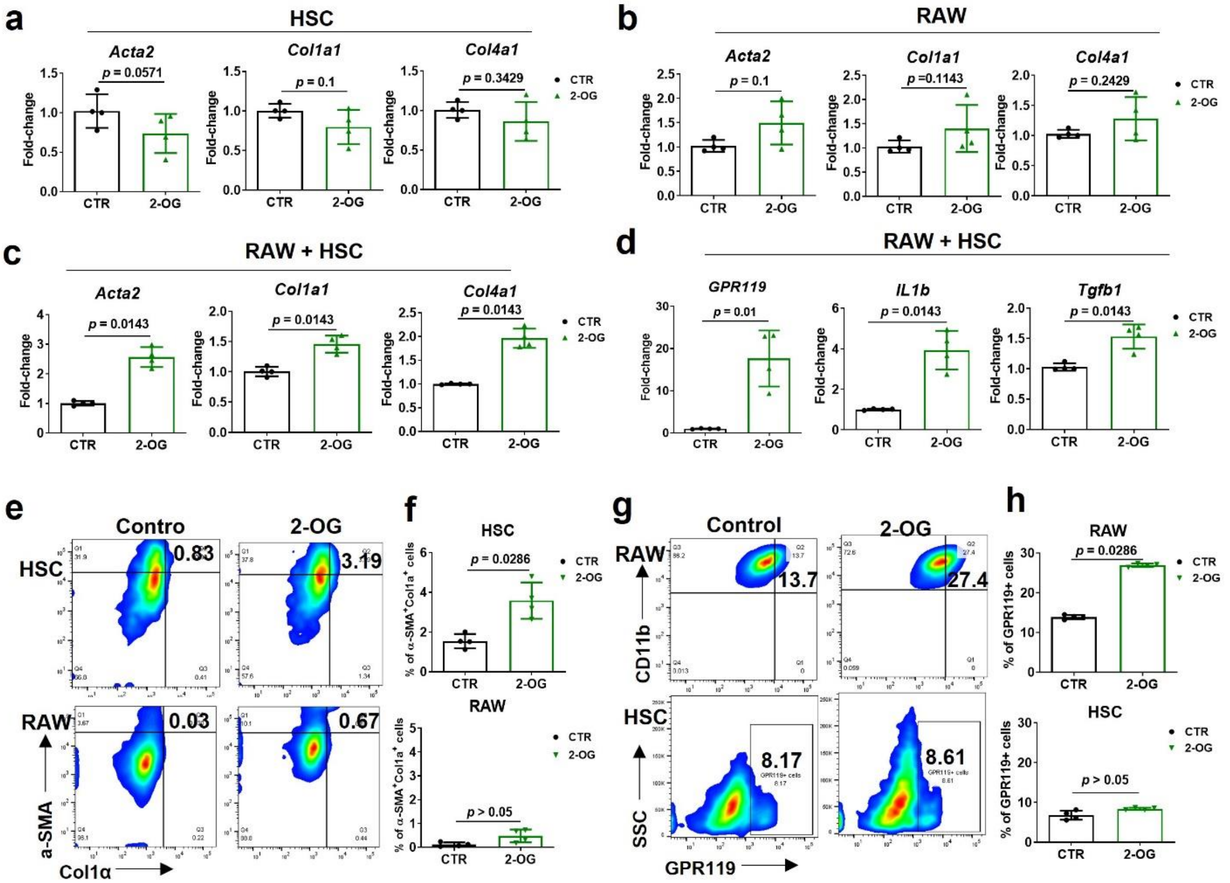

Supplementary Fig. 15. 2-OG activates HSC in the presence of MΦ by promoting GPR119 expression. Mouse HSCs, RAW264.7 cells, or co-cultured HSCs and RAW264.7 cells at a ratio of 1:1 did or did not receive stimulation with 2-OG at a dose of 50 μg/mL for 24 hours. qPCR was used to detect the mRNA expression of different genes. **a** Effect of 2-OG stimulation on ECM genes *Acta2*, *Col1a1*, and *Col4a1* in HSCs. **b** Effect of 2-OG stimulation on ECM genes in RAW264.7 cells. **c** Effect of 2-OG stimulation on ECM genes in the co-cultured cells. qPCR detected a significant increase in the mRNA expression of ECM genes *Acta2*, *Col1a1*, and *Col4a1*. **d** Expression of GPR119 and inflammatory cytokines in co-cultured cells in response to 2-OG stimulation. qPCR detected a significant increase in the mRNA expression of genes *Gpr119*, *Il1b*, and *Tgfb1* in 2-OG-treated cells. **e** HSC activation in co-cultured cells in response to 2-OG stimulation. Representative flow cytometry showed that 2-OG stimulation induced a significant increase in the frequency of HSCs positive for α-SMA and Col1α, but not RAW264.7 cells positive for CD11b and CD45. **f** Mean frequency of HSCs and RAW264.7 cells in **e**. The cumulative data showed that 2-OG stimulation induced a significant increase in the frequency of activated HSCs, but not RAW264.7 cells. **g** GPR119 expression in the co-cultured HSCs and RAW264.7 cells. Representative flow cytometry showed that 2-OG stimulation led to a significant

increase in the expression of GPR119 in $CD11b^{+}CD45^{+}$ RAW264.7 cells, but not $CD11b^{-}CD45^{-}$ HSCs. **h** Mean frequency of GPR119-expressing HSCs and RAW264.7 cells. The cumulative data showed that 2-OG stimulation induced a significant increase in the frequency of RAW264.7 cells, but not HSCs, expressing GPR119. n=4, data are presented as mean ± SD. The assay was repeated twice. Statistical analysis of data was performed by Mann–Whitney test (one-tailed for **a**-**d**; two-tailed for **f** and **h**). Source data are provided as a Source Data file.

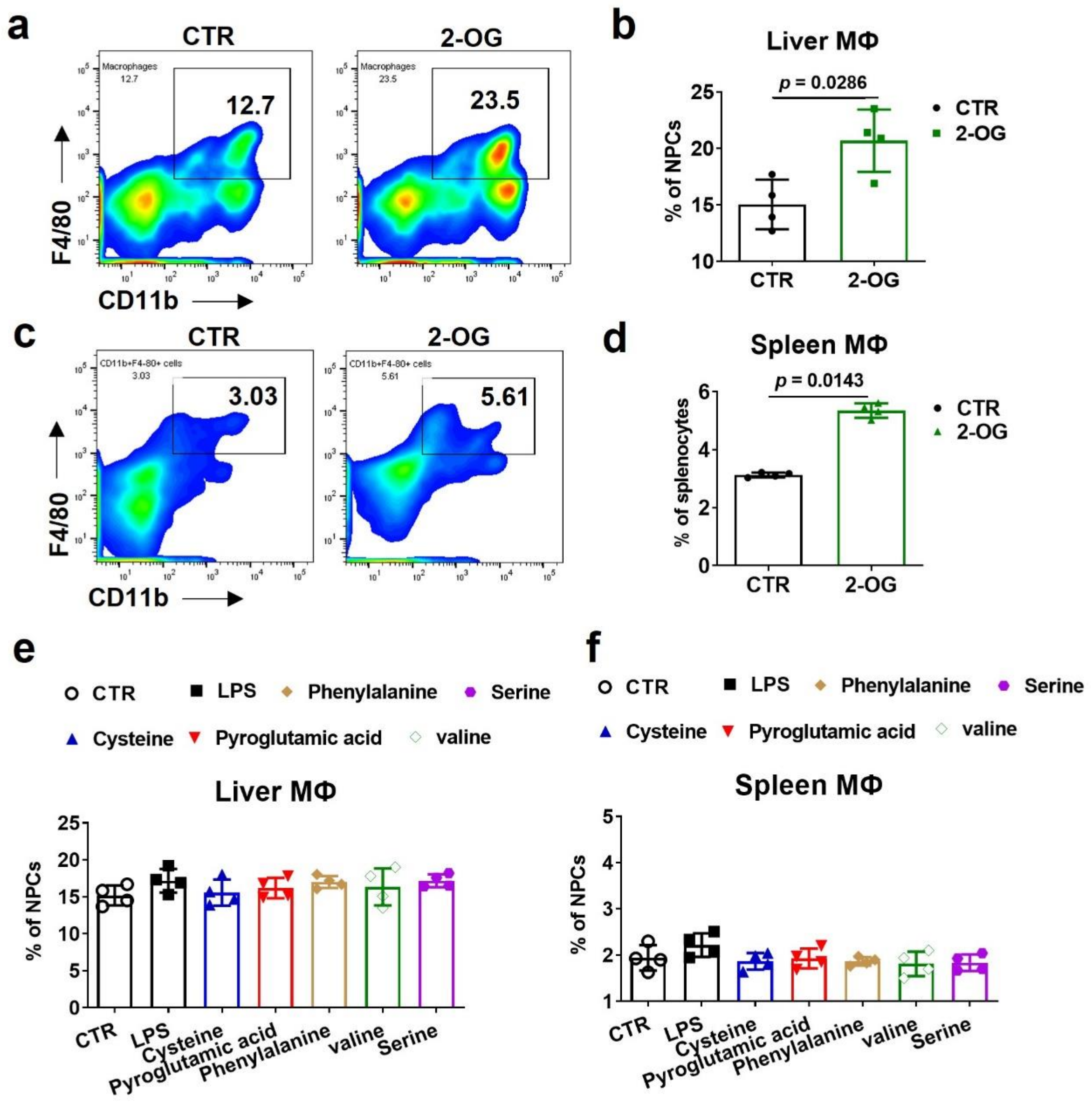

Supplementary Fig. 16. 2-OG stimulates MΦ proliferation in hepatic NPCs and splenocytes. **a** 2-OG stimulates MΦ proliferation in NPCs. Isolated hepatic NPCs from WT mice did or did not receive 2-OG stimulation for 24 hours. Representative flow cytometry detected a one-fold increase in the frequency of $CD11b^{+}F4/80^{+}$MΦs in 2-OG stimulated NPCs. **b** Mean frequency of MΦs in 2-OG-stimulated hepatic NPCs. The cumulative results of flow cytometry indicated that 2-OG stimulation led to a significant increase in the frequency of $CD11b^{+}F4/80^{+}$MΦs in NPCs. **c** 2-OG stimulates MΦ proliferation in splenocytes. RBC-depleted splenocytes did or did not receive 2-OG stimulation for 24 hours. Flow cytometry detected an increase in the frequency of $CD11b^{+}F4/80^{+}$MΦs in 2-OG stimulated splenocytes. **d** Mean frequency of $CD11b^{+}F4/80^{+}$MΦs in 2-OG stimulated splenocytes. Cumulative results of flow cytometry indicated that 2-OG stimulation significantly increased the frequency of $CD11b^{+}F4/80^{+}$MΦs in the splenocytes. **e** The

effect of other metabolites on MΦ generation in hepatic NPCs. Isolated hepatic NPCs from WT mice were treated with the indicated metabolites for 24 hours and then underwent flow cytometry. The cumulative results showed that the indicated metabolites did not induce a significant change in the mean frequency of MΦs. **f** The effect of other metabolites on MΦ generation in splenocytes. Isolated splenocytes from WT mice were treated with the indicated metabolites for 24 hours and then underwent flow cytometry. The cumulative results showed that the indicated metabolites did not induce a significant change in the mean frequency of MΦs. n=4, data are presented as mean ± SD. The assay was repeated twice. Statistical analysis of data was performed by Mann–Whitney test (one-tailed). Source data are provided as a Source Data file.

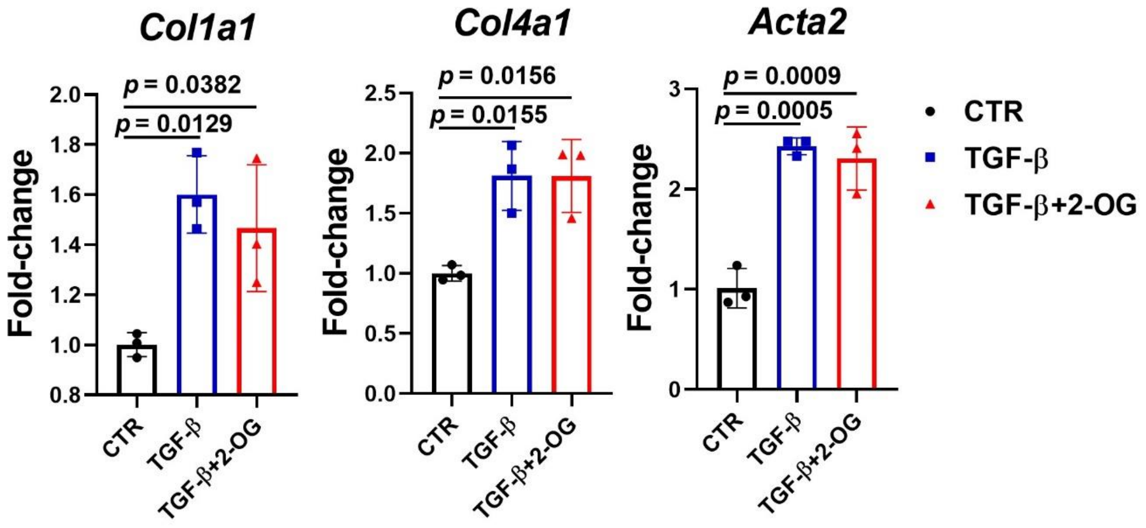

Supplementary fig. 17. 2-OG does not promote TGF-β-induced HSC activation. HSCs were stimulated with TGF-β (10 ng/mL) in the presence or absence of 2-OG co-stimulation (50 µg/mL) for 24 hours. qPCR detected a significant increase of gene expression of *Col1a1*, *Col4a1*, and *Acta2* in TGF-β-stimulated HSCs. However, the effect was not further enhanced by 2-OG treatment. n=3, data are presented as mean ± SD. The assay was repeated twice. Statistical analysis of data was performed by one-way ANOVA with Tukey's multiple comparison test. Source data are provided as a Source Data file.

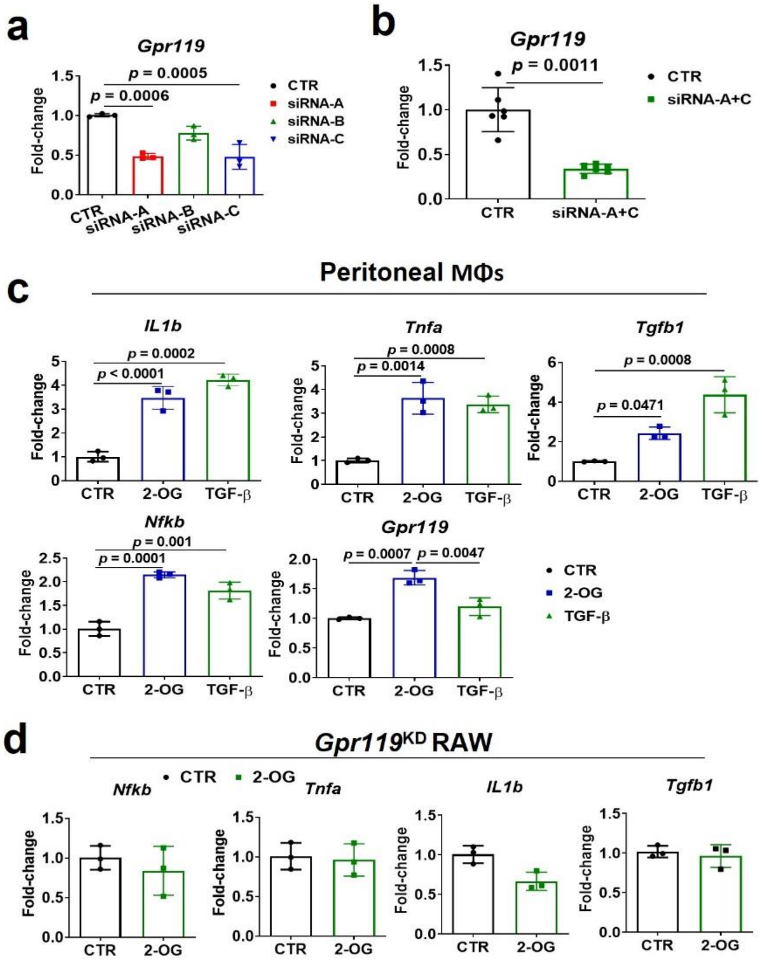

Supplementary Fig. 18. GRP119 is required for 2-OG to activate MΦs. a, **b** Validation of siRNA for *Gpr119* knockdown. Three genome-wide siRNAs for *Gpr119* were transfected into RAW264.7 cells. 48 hours later, the cells were harvested to extract total RNAs for qPCR assay. The results showed that siRNA-A, siRNA-C (**a,** n=3), or their combination (**b,** n=6), but not siRNA-B, significantly suppressed GPR119 expression in RAW264.7 cells. **c** 2-OG activates peritoneal MΦs. Isolated peritoneal MΦs from WT mice received 2-OG or TGF-β (10 ng/mL, positive control) stimulation for 24 hours. qPCR detected significantly increased mRNA expression of genes *IL1b*, *Tnfa*, *Tgfb1*, and *Nfkb* in both 2-OG and TGF-β-stimulated MΦs; however, a significant increase in *Gpr119* expression was only detected in 2-OG-stimulated MΦs. n=3. **d** 2-OG was unable to activate RAW264.7 cells with siRNA-mediated *Gpr119* knockdown. RAW264.7 cells received siRNAs transfection for 48 hours to knock down *Grp119*, followed by 2-OG stimulation for 24 hours. After that, the cells were harvested to extract total RNAs for qPCR assay. The results showed that 2-OG stimulation did not significantly change the production of *Nfkb*, *Tnfa*, *IL1b*, and *Tgfb1* in RAW264.7 cells with *Gpr119* knockdown. n=3. Data are presented as mean ± SD. The

assay was repeated twice. Statistical analysis of data was performed by Mann–Whitney test (one-tailed) or one-way ANOVA with Tukey's multiple comparison test. Source data are provided as a Source Data file.

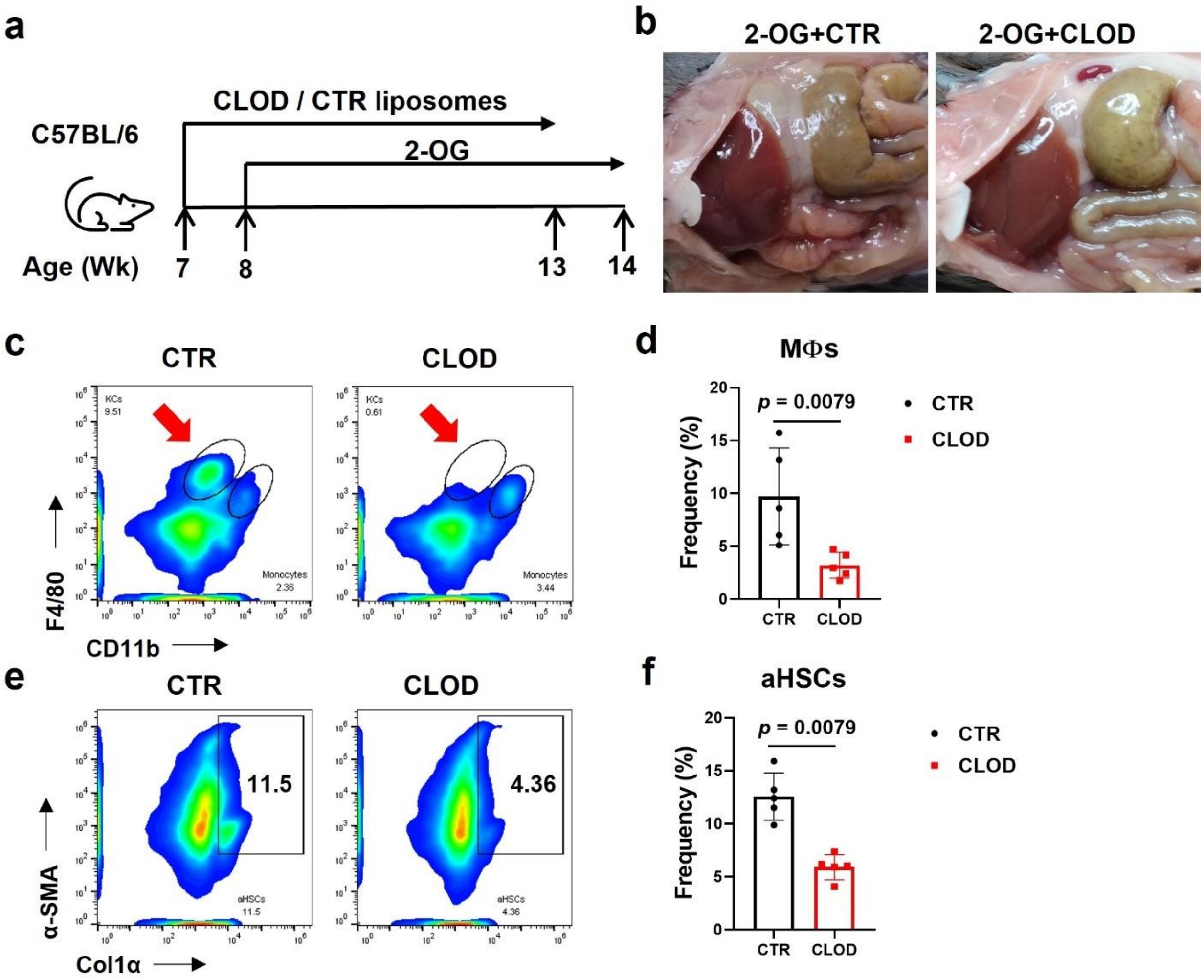

Supplementary Fig. 19. Depletion of liver resident MΦs suppresses 2-OG-mediated HSC activation. a An outline depicting the treatment of mice with clodronate liposomes (CLOD) and 2-OG. Seven-week-old WT mice received I.P. injection of CLOD once a week for 7 weeks to deplete MΦs. Control liposome (CTR) was used for control. One week after the final treatment, mice received 2-OG i.v. injection three times a week for 6 weeks at a dose of 20 μg/mouse in 0.2 mL PBS (Fig. 6). After that, all mice were euthanized to isolate hepatic NPCs for the following studies. **b** Macroscopic images of the liver in the mice treated with liposomes and 2-OG. **c** Representative frequencies of the liver-resident MΦs in NPCs. Representative flow cytometry analysis showed that CLOD injection caused depletion of one major liver resident MΦs (Kupffer cells), but not monocytes-derived MΦs. **d** Mean frequency of MΦs in NPCs in control and CLOD-treated mice. **e** Representative frequencies of the activated HSCs expressing Col1α and α-SMA in NPCs in control and CLOD-treated mice. **f** Mean frequencies of activated HSCs expressing Col1α and α-SMA in NPCs in control and CLOD-treated mice. Cumulative data showed that

CLOD injection caused a significant decrease in the frequency of activated HSCs expressing Col1α and α-SMA in NPCs. n=5, data are presented as mean ± SD. Statistical analysis of data was performed by Mann–Whitney test (two-tailed). Source data are provided as a Source Data file.

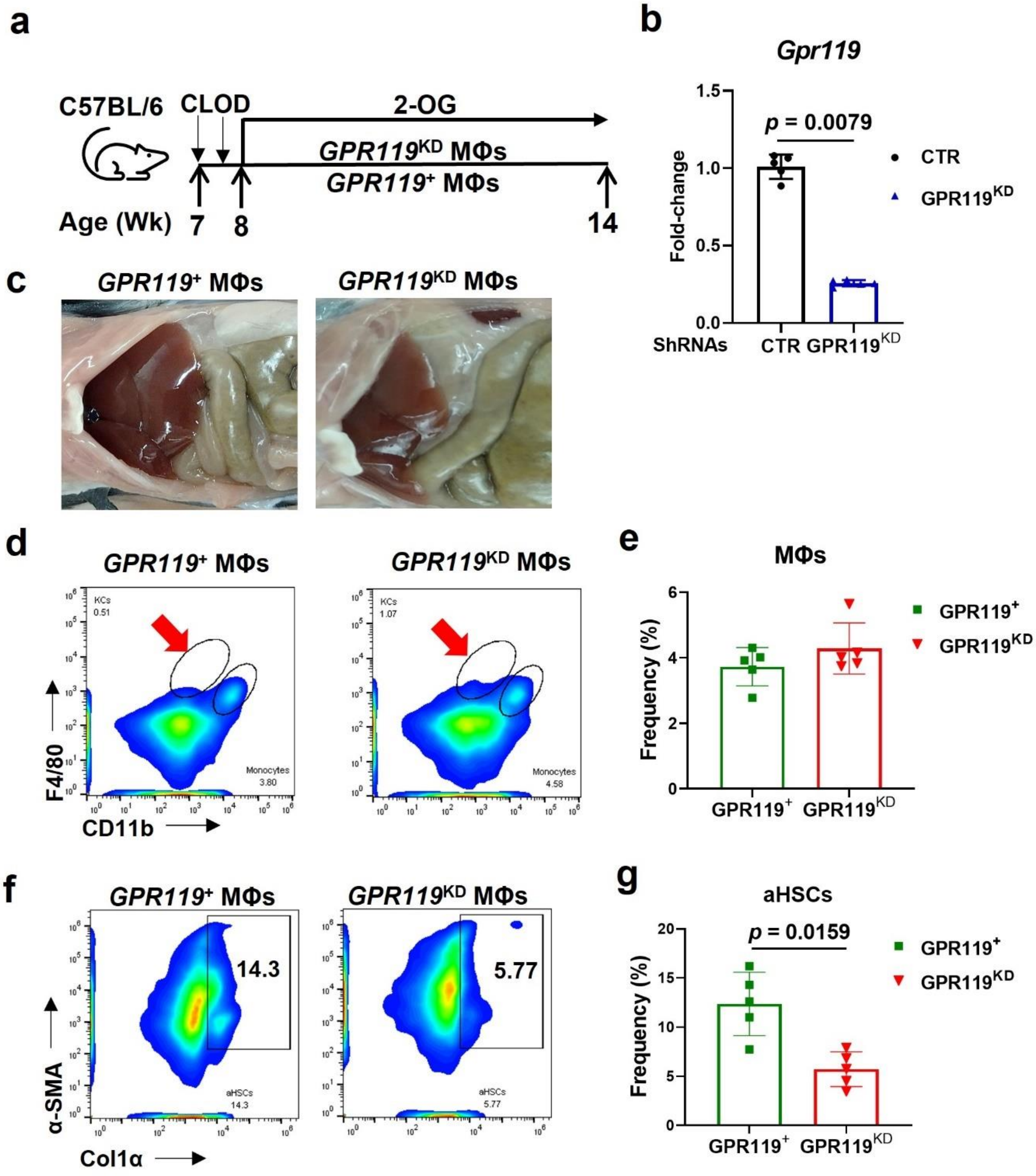

Supplementary Fig. 20. Adoptive transfer of MΦs with *Gpr119*-knockdown does not enable 2-OG-mediated HSC activation in MΦ-depleted mice. a An outline depicting the experimental design for treating mice with CLOD, 2-OG, and adoptive transfer of MΦs with or without *GPR119*-knockdown. Seven-week-old WT mice received two I.P. injections of CLOD to deplete liver resident MΦs. Control liposomes (CTR) were used for control. After that, mice received i.v. injection of 2-OG three times a week for 6 weeks at a dose of 20 μg/mouse in 0.2 mL PBS (Fig. 6). Simultaneously, mice received the adoptive transfer of MΦs with or without *Gpr119*-

knockdown once a week for six times at a dose of 2×10^6 cells/mouse. Then, all mice were euthanized to isolate NPCs for the following studies. **b** Validation of shRNA-expressing lentivirus capacity to knock down *Gpr119* in RAW264.7 cells. qPCR detected that *Gpr119* expression was significantly decreased in MΦs with infection of *Gpr119*-shRNA-expressing lentiviruses versus control lentivirus. **c** Macroscopic images of livers in mice receiving the indicated treatments with adoptive transfer of WT MΦs or *Gpr119*-knockdown MΦs. **d** Representative frequencies of liver-resident MΦs in NPCs. Representative flow cytometry analysis showed that CLOD injection caused depletion of one major liver resident MΦs (Kupffer cells), but not monocytes-derived MΦs. **e** Mean frequencies of liver-resident MΦs in NPCs in two groups of mice (p > 0.05). **f** Representative frequencies of the activated HSCs expressing Col1α and α-SMA in NPCs in mice receiving the indicated treatments with adoptive transfer of WT MΦs or *Gpr119*-knockdown MΦs. **g** Mean frequencies of activated HSCs expressing Col1α and α-SMA in NPCs in two groups of mice. Cumulative data showed that the adoptive transfer of WT MΦs, but not *Gpr119*-knockdown MΦs, caused an increase in the frequency of the activated HSCs expressing Col1α and α-SMA in NPCs. n=5, data are presented as mean ± SD. Statistical analysis of data was performed by Mann–Whitney test (two-tailed). Source data are provided as a Source Data file.

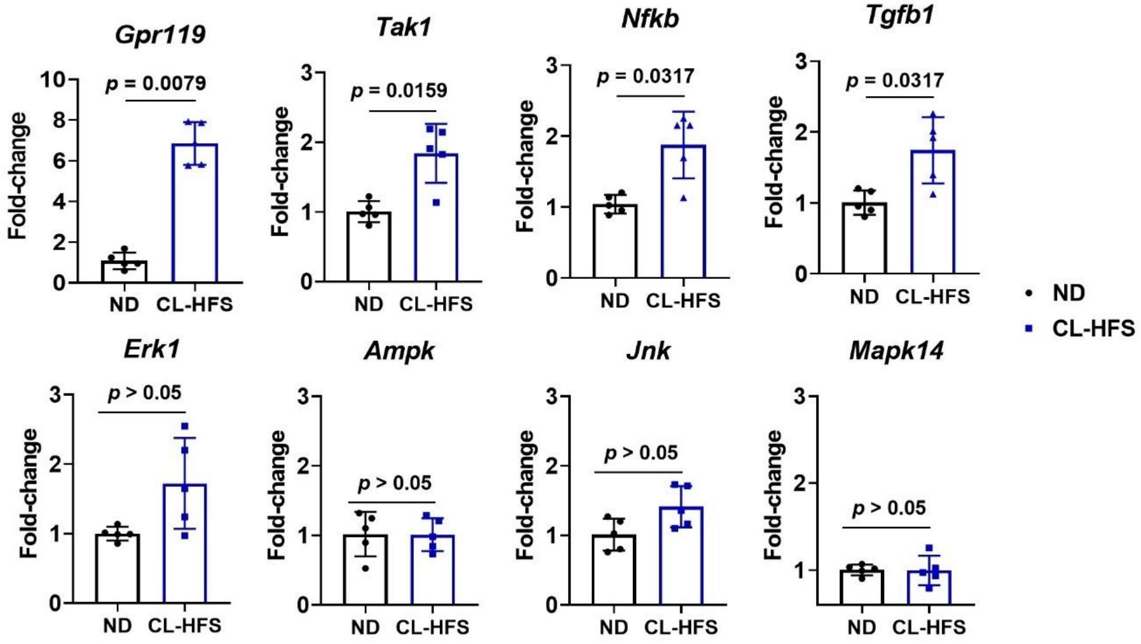

Supplementary Fig. 21. The signaling events of GPR119 in mouse hepatic MΦs. Hepatic NPCs were freshly isolated from WT mice that received ND or CL-HFS for 12 weeks. MΦs were purified from isolated NPCs with anti-F4/80 magnetic beads for extraction of total RNAs. qPCR detected significantly increased gene expression of *Gpr119*, *Tak1* (Mitogen-activated protein kinase kinase 7/MAP3K7 or TAK1), *Nfkb*, and *Tgfb1*, but not *Erk1* (Ras-dependent extracellular signal-regulated kinase 1), *AMPK* (AMP-activated protein kinase), JNK (c-Jun N-terminal kinase), and *Mapk14* (Mitogen-activated protein kinase 14/p38α), in the MΦs isolated from CL-HFS-fed mice versus ND-fed mice. n=5, data are presented as mean ± SD. Statistical analysis of data was performed by Mann–Whitney test (two-tailed). Source data are provided as a Source Data file.

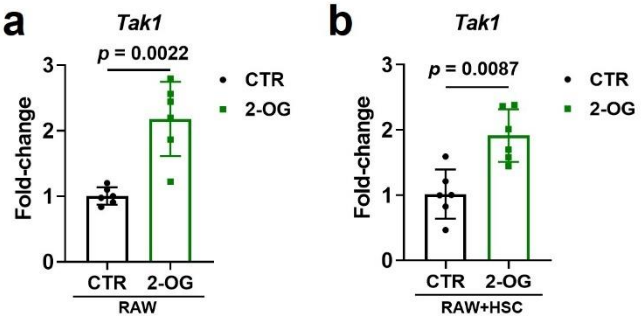

Supplementary Fig. 22. 2-OG stimulation induces increased TAK1 expression in single or co-cultured RAW264.7 cells with HSCs. RAW264.7 cells were stimulated with 2-OG for 24 hours in the presence or absence of HSCs. Total RNAs in RAW264.7 cells or co-cultured cells were extracted for qPCR assay. **a** qPCR detected significantly increased mRNA expression of *Tak1* in 2-OG-stimulated RAW264.7 cells versus control cells. **b** qPCR detected significantly increased mRNA expression of *Tak1* in co-cultured cells. n=6, data are presented as mean ± SD. The assay was repeated twice. Statistical analysis of data was performed by Mann–Whitney test (Two-tailed). Source data are provided as a Source Data file.

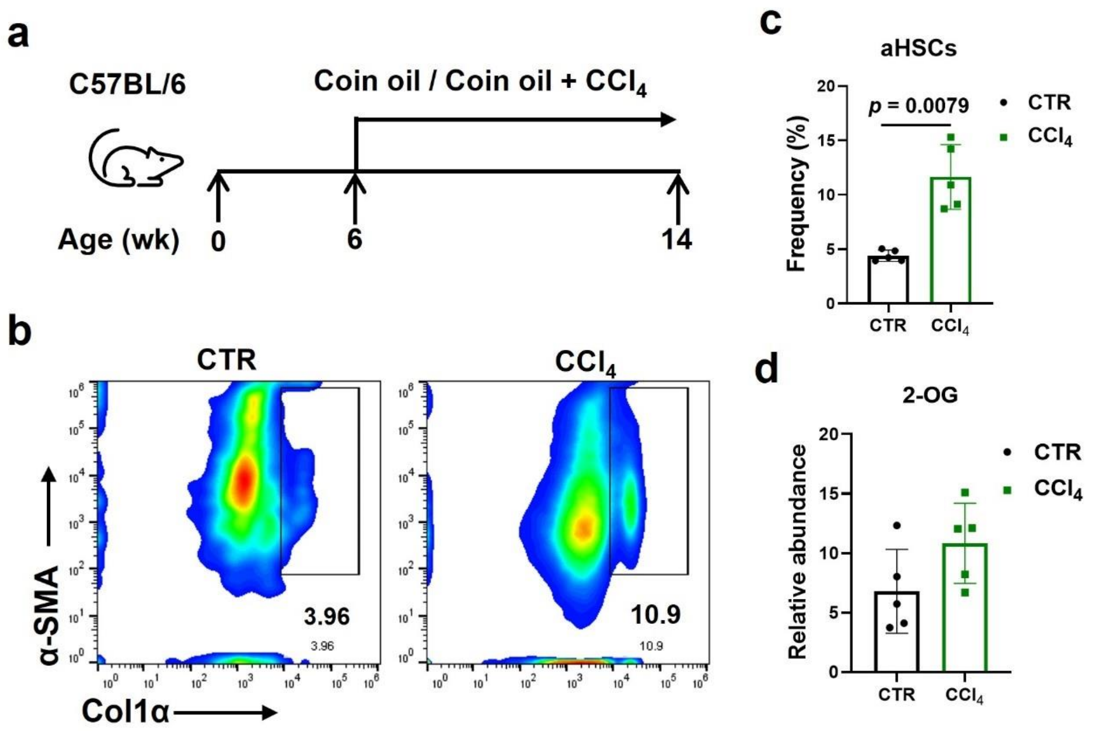

Supplementary Fig. 23. CCL$_4$ treatment does not induce 2-OG production. a An outline depicting CCL$_4$ treatment for inducing mouse liver fibrosis. Six-week-old WT mice received I.P. injection of 10% (v/v) CCL$_4$ in corn oil twice a week for 8 weeks at 8 mL/kg of body weight to induce liver fibrosis. Coin oil was used as a control. After the treatment, mice were euthanized for the following studies. **b** Representative flow cytometric analysis showed that CCL$_4$ treatment led to an increase in the frequency of activated HSCs expressing Col1α and α-SMA. **c** The mean frequency of activated HSCs expressing α-SMA and Col1α. **d** The relative abundance of 2-OG in livers of control and CCL$_4$-treated mice ($p > 0.05$). n=5, data are presented as mean ± SD. Statistical analysis of data was performed by Mann–Whitney test (two-tailed). Source data are provided as a Source Data file.

Supplementary Tables

Supplementary Table 1. List of liver metabolites identified by spectral matching

Number	Metabolite	Retention time (min)	Quantitative ion (m/z)
1	Trisiloxane, Octamethyl-*	9.1391	87.1
2	Trisiloxane, Octamethyl-*	9.1411	95.2
3	Methylamine, 2TMS derivative*	9.498	64.6
4	Methylamine, 2TMS derivative*	9.5076	160.2
5	Bis-trimethylsilyl amine	9.5125	116.1
6	2-(Dimethylamino)ethanol, TMS derivative	9.9465	58.2
7	1,2,5-Thiadiazole, 3-methyl-	10.5017	101.1
8	4-But-1-enyltrimethylsilane	10.5203	113.1
9	Silanamine, N-Methoxy-1,1,1-trimethyl-N-(trimethylsilyl)-*	10.6195	74.2
10	Ethanol, 2-(methylamino)-	10.7201	44.2
11	Ethylbis(trimethylsilyl)amine	10.9715	174.2
12	Silanamine, N,N'-methanetetraylbis[1,1,1-trimethyl-*	10.9929	171.2
13	Disilathiane, hexamethyl-*	11.1251	163.1
14	Disilathiane, hexamethyl-*	11.1396	144.2
15	L-Proline, TMS derivative*	12.7061	70.2
16	4-Hydroxypyridine,O-TMS	13.0878	152.2
17	Lactic Acid O,O-TMS*	13.4749	46.1
18	Lactic Acid, O,O-TMS*	13.4791	117.2
19	Oxalic acid, 2TMS derivative	13.486	88.9
20	2,2'-Bithiazolidine	14.6585	87.7
21	L-Alanine, N,O-TMS*	14.6709	149.1
22	L-Alanine, N,O-TMS*	14.6736	116.2
23	Glycine, N,O-TMS	15.1669	102.1
24	1,2-Bis(trimethylsiloxy)ethane	16.1926	75.1
25	Pentasiloxane Dodoecamethyl	16.3029	282.1
26	Phosphoric Acid, Monomethyl ester, O,O-TMS	16.7162	241.1
27	Disiloxane, Hexamethyl	17.2211	216.2
28	L-Valine, N,O-TMS*	17.8046	218.2
29	L-Valine, 2TMS derivative	17.8074	144.2
30	Urea, 2TMS derivative*	18.488	45.1
31	Urea, 2TMS derivative*	18.4956	189.2
32	Urea, N,N-TMS*	18.8366	189.2
33	Ethanol Amine, N,O,O-TMS*	19.2837	174.2
34	Ethanol Amine, N,O,O-TMS*	19.2864	211.1
35	Ethanol Amine, N,O,O-TMS*	19.2926	175.2
36	Silanol, trimethyl-, phosphate (3:1)*	19.3037	314.2
37	Glycerol, 3TMS derivative*	19.3119	173.1
38	Glycerol, 3TMS derivative*	19.3347	175.2
39	Glycerol, 3TMS derivative*	19.3381	101.1
40	Glycerol, 3TMS derivative*	19.3402	117.1
41	Silanol, trimethyl-, phosphate (3:1)*	19.3416	205.2

42	Silanol, trimethyl-, phosphate (3:1)*	19.3471	191.1
43	Silanol, trimethyl-, phosphate (3:1)*	19.3505	130.1
44	Norleucine, N,O-TMS	19.3588	102.1
45	L-Leucine, 2TMS derivative*	19.3643	232.2
46	L-Leucine, 2TMS derivative*	19.3677	158.2
47	L-Leucine, 2TMS derivative*	19.3774	100.1
48	L-Norleucine, 2TMS derivative	19.3912	234.2
49	L-Norleucine, 2TMS derivative	19.3939	146.1
50	Phosphoric Acid, O,O,O-TMS	19.4042	74.1
51	Silanol, trimethyl-, phosphate (3:1)*	19.4104	75.1
52	Silanol, trimethyl-, phosphate (3:1)*	19.4153	317.2
53	Silanol, trimethyl-, phosphate (3:1)*	19.4187	301.2
54	L-Isoleucine, 2TMS derivative	19.9781	45.1
55	L-Proline, 2TMS derivative*	20.2137	142.2
56	L-Proline, N,O-TMS*	20.2178	72.1
57	Glycine, N,N,O-TMS	20.4162	174.2
58	Uracil, O,O-TMS	21.2016	99.1
59	Fumaric Acid, O,O-TMS	21.4744	245.1
60	L-Serine, N,O,O-TMS*	21.6796	66.1
61	L-Serine, N,O,O-TMS*	21.6824	205.2
62	L-Serine, N,O,O-TMS*	21.6838	204.2
63	L-Alanine, N,N,O-TMS*	21.8512	188.2
64	L-Threonine, N,O,O-TMS	22.3568	204.2
65	L-Aspartic Acid, O,O-TMS	23.479	130.1
66	Beta-Alanine, N,O,O-TMS	23.5748	248.2
67	Homoserine, N,O,O-TMS	24.0143	218.2
68	Butanal, 2,3,4-tris[(trimethylsilyl)oxy]-, O-methyloxime, [R-(R*,R*)]-	24.3677	103.1
69	Malic acid, 3TMS derivative	24.9395	45.1
70	Niacin, O-TMS	25.2694	75.1
71	Asparagine [-H2O] (2TMS)	25.545	100.1
72	3-Amino-2-piperidone, 2TMS derivative	25.5567	115.1
73	L-Aspartic acid, N,O,O-TMS	25.802	232.2
74	L-Methionine, N,O-TMS	25.9087	128.2
75	Pyroglutamic acid (2TMS)	25.9225	202.2
76	Pyroglutamic Acid, N,O-TMS*	26.0631	158.2
77	Pyroglutamic Acid, N,O-TMS*	26.0644	156.2
78	Silane, tetramethyl-*	26.0665	231.2
79	4-Aminobutyric Acid, N,O,O-TMS	26.165	75.1
80	Golm_Cysteine (3TMS)	26.703	220.2
81	L-Phenylalanine, O-TMS	26.8677	91.2
82	Proline [+CO2] (2TMS)	27.4243	186.2
83	L-Glutamic acid (3TMS)	28.1497	246.2
84	L-Phenylalanine, N,O-TMS*	28.5382	267.2
85	D-(-)-Ribose, O,O,O,O-TMS, MEOX*	29.3408	231.2
86	D-(-)-Ribose, O,O,O,O-TMS, MEOX*	29.3422	100.2
87	Xylose, O,O,O,O-TMS, MEOX2	29.3456	103.1
88	Xylitol, O,O,O,O,O-TMS	29.9883	307.2
89	Beta-glycerophosphate, O,O,O,O-TMS	30.4224	227.1

90	Ribitol (5TMS)	30.4423	205.2
91	Alpha-glycerophosphate ester, 4TMS	31.2166	357.2
92	Ribonic acid, 2,3,4,5-tetrakis-O-(trimethylsilyl)-, trimethylsilyl ester*	31.2656	103.1
93	Ribonic acid, 2,3,4,5-tetrakis-O-(trimethylsilyl)-, trimethylsilyl ester*	31.2683	292.2
94	Ornithine N,N,N,O-TMS 1	31.289	174.2
95	Lyxonic acid (5TMS)	31.5129	205.2
96	Ribonic acid, 2,3,4,5-tetrakis-O-(trimethylsilyl)-, trimethylsilyl ester*	31.5163	292.2
97	Ethanolaminephosphate (4TMS)	31.818	315.2
98	L-(-)-Sorbofuranose, pentakis(trimethylsilyl) ether	32.1349	75.1
99	9H-Purin-6-ol, 2TMS derivative	32.5469	265.2
100	6-Hydroxypurine, N,O-TMS	32.5662	142.2
101	Tetradecanoic Acid, O-TMS	33.2964	285.2
102	L-Lysine, N6,N6-bis(trimethylsilyl)-, trimethylsilyl ester	33.4335	156.2
103	D-(-)-Fructose, O,O,O,O,O-TMS, MEOX1*	33.5375	174.2
104	D-Fructose, 1,3,4,5,6-pentakis-O-(trimethylsilyl)-, O-methyloxime*	33.5423	218.2
105	D-Fructose, 1,3,4,5,6-pentakis-O-(trimethylsilyl)-, O-methyloxime*	33.5437	103.1
106	D-Fructose, 1,3,4,5,6-pentakis-O-(trimethylsilyl)-, O-methyloxime*	33.5465	205.2
107	D-(-)-Fructose, O,O,O,O,O-TMS o-methyloxime 1*	33.5548	168.1
108	D-(-)-Fructose, pentakis(trimethylsilyl) ether, methyloxime (syn)*	33.7242	190.1
109	D-(-)-Fructose, O,O,O,O,O-TMS, MEOX2	33.7511	104.1
110	D-(-)-Fructose, pentakis(trimethylsilyl) ether, methyloxime (syn)*	33.7573	307.2
111	D-(-)-Fructose, pentakis(trimethylsilyl) ether, methyloxime (syn)*	33.7635	74.1
112	L-(-)-Sorbose, pentakis(trimethylsilyl) ether, methyloxime (syn)	33.7683	133.1
113	D-(+)-Talose, pentakis(trimethylsilyl) ether, methyloxime (syn)*	33.8537	163.1
114	D-Allose, pentakis(trimethylsilyl) ether, methyloxime (syn)*	33.8599	343.3
115	D-(+)-Talose, pentakis(trimethylsilyl) ether, methyloxime (syn)*	33.8758	133.1
116	D-(+)-Mannose, O,O,O,O,O-TMS, MEOX1*	33.8792	319.2
117	D-(+)-Talose, pentakis(trimethylsilyl) ether, methyloxime (syn)*	33.8875	291.2
118	D-(+)-Glucose, O,O,O,O,O-TMS o-methyloxime 1*	33.893	205.2
119	D-Glucose, 2,3,4,5,6-pentakis-O-(trimethylsilyl)-, o-methyloxyme, (1E)-*	33.8992	204.2

120	1,2-Ethenediol, 2TMS derivative	34.0528	349.2
121	D-Glucose, 2,3,4,5,6-pentakis-O-(trimethylsilyl)-, o-methyloxyme, (1Z)-*	34.1134	204.2
122	D-(+)-Talose, pentakis(trimethylsilyl) ether, methyloxime (syn)*	34.121	191.2
123	D-(+)-Mannose, O,O,O,O,O-TMS, o-methyloxime 1*	34.141	236.2
124	D-(+)-Glucose, O,O,O,O,O-TMS, MEOX1*	34.1486	189.2
125	D-(+)-Mannose O,O,O,O,O-TMS meox 1*	34.1562	276.3
126	D-(+)-Talose, pentakis(trimethylsilyl) ether, methyloxime (syn)*	34.172	161.2
127	D-Glucose, 2,3,4,5,6-pentakis-O-(trimethylsilyl)-, o-methyloxyme, (1Z)-	34.1782	229.2
128	L-Tyrosine, O-trimethylsilyl-, trimethylsilyl ester*	34.1968	179.2
129	D-Glucose, 2,3,4,5,6-pentakis-O-(trimethylsilyl)-, o-methyloxyme, (1Z)-	34.2044	320.3
130	D-(+)-Glucose O,O,O,O,O-TMS meox 1*	34.2168	366.3
131	D-Glucose, 2,3,4,5,6-pentakis-O-(trimethylsilyl)-, O-methyloxime	34.2257	161.2
132	D-(+)-Talose, pentakis(trimethylsilyl) ether, methyloxime (syn)*	34.2519	101.2
133	D-(+)-Talose, pentakis(trimethylsilyl) ether, methyloxime (syn)*	34.2533	231.2
134	D-Glucose, 2,3,4,5,6-pentakis-O-(trimethylsilyl)-, O-methyloxime	34.2581	291.3
135	D-(+)-Talose, pentakis(trimethylsilyl) ether, methyloxime (syn)*	34.2629	205.3
136	D-(+)-Galactose, O,O,O,O,O-TMS, MEOX2*	34.336	205.2
137	D-(+)-Mannose O,O,O,O,O-TMS meox 2*	34.5784	396.3
138	D-Allose, pentakis(trimethylsilyl) ether, methyloxime (anti)*	34.5915	174.2
139	D-(+)-Talose, pentakis(trimethylsilyl) ether, methyloxime (anti)*	34.5984	132.2
140	D-Allose, pentakis(trimethylsilyl) ether, methyloxime (anti)*	34.6081	319.4
141	D-(+)-Galactose O,O,O,O,O-TMS meox 2*	34.6142	174.2
142	2-Ethylhexanal ethylene glycol acetal	34.6212	41.1
143	L-Lysine, N,N,N,O-TMS	34.7575	154.2
144	D-Mannitol, O,O,O,O,O,O-TMS*	34.9174	75.1
145	D-Mannitol, O,O,O,O,O,O-TMS*	34.9194	319.2
146	Methyl hexadecanoate	34.9828	74.1
147	Hexadecanoic acid, methyl ester	34.9869	270.3
148	L-Tyrosine, N,O-bis(trimethylsilyl)-, trimethylsilyl ester*	35.1075	219.2
149	L-Tyrosine, N,O-bis(trimethylsilyl)-, trimethylsilyl ester*	35.1096	218.2
150	D-(+)-Turanose, octakis(trimethylsilyl) ether	35.299	361.3
151	D-Gluconic acid, 6TMS derivative*	35.4864	319.2

152	Galactonic Acid, O,O,O,O,O,O-TMS	35.4898	333.2
153	Gluconic Acid (6TMS)*	36.1415	205.2
154	Xanthine, N,O,O-TMS	36.5817	354.2
155	7-(Trimethylsilyl)-2,6-bis[(trimethylsilyl)oxy]-7H-purine	36.5866	353.2
156	Hexadecenoic acid, 9-(Z)-(1TMS)	36.7051	117.1
157	Palmitelaidic acid, TMS derivative*	36.7106	311.3
158	Palmitelaidic acid, TMS derivative*	36.8132	75.1
159	2-Tridecanol, TMS derivative	37.2245	119.1
160	Hexadecanoic Acid, O-TMS*	37.2293	313.4
161	Hexadecanoic Acid, O-TMS*	37.232	75.1
162	Myo-Inositol, O,O,O,O,O,O-TMS	37.9278	305.2
163	Methyl linoleate	38.211	67.1
164	Oleic acid*	38.3081	55.2
165	D-Galactose, 2,3,4,5,6-pentakis-O-(trimethylsilyl)-, o-methyloxyme, (1Z)-*	38.5919	321.2
166	D-Galactose, 2,3,4,5,6-pentakis-O-(trimethylsilyl)-, o-methyloxyme, (1Z)-*	38.5967	205.2
167	D-(+)-Galactose, pentakis(trimethylsilyl) ether, ethyloxime (isomer 2)*	38.7104	319.2
168	Heptadecanoic acid, O-TMS	38.966	43.2
169	Octadecatrienoic acid, 6,9,12-(Z,Z,Z)-, n-1TMS	39.874	41.2
170	9,12-Octadecadienoic acid (Z,Z)-, TMS derivative	40.2473	102.1
171	Linoleic Acid, O-TMS	40.2597	202.2
172	Octadecenoic acid, 9-(Z)- (1TMS)	40.3548	202.2
173	Cyclohexanol, 2-(trimethylsilyl)-, cis-	40.3651	161.2
174	Oleic Acid, (Z)-, TMS derivative*	40.3734	41.2
175	9-Octadecenoic acid, (E)-, TMS derivative	40.4299	75.1
176	Glyceryl-glycoside TMS ether	40.6386	205.2
177	Stearic Acid, O-TMS	40.7233	117.1
178	Alpha-Farnesene; 1,3,6,10-Dodecatetraene, 3,7,11-trimethyl-	41.1615	79.1
179	Galactosyl glycerol 6TMS	41.3578	129.1
180	Glyceryl-glycoside TMS ether	41.3592	204.2
181	2-O-Glycerol-alpha-d-galactopyranoside, hexa-TMS*	41.4866	204.2
182	Eicosatetraenoic acid, 5,8,11,14-(Z,Z,Z,Z)-(1TMS)*	42.9058	75.1
183	Eicosapentaenoic Acid, TMS derivative*	42.9946	78.2
184	Eicosapentaenoic acid, TMS derivative*	43.0077	75.1
185	(8Z,11Z,14Z)-Icosa-8,11,14-trienoate, O-TMS	43.2082	75.1
186	5-Methyluridine, 3TMS derivative*	43.3115	259.2
187	5-Methyluridine, 3TMS derivative*	43.317	183.1
188	11,14-Eicosadienoic acid, TMS derivative	43.5189	75.1
189	Uridine, 4TMS derivative	43.9467	243.2
190	Uridine (3TMS)	44.1485	75.1
191	Arachidonic acid, TMS derivative*	45.735	79.1

192	Doconexent, TMS derivative	45.8762	91.2
193	Arachidonic acid, TMS derivative*	45.9913	80.2
194	1-Monopalmitin, 2TMS derivative	46.096	371.4
195	Docosahexaenoic acid, 4,7,10,13,16,19-(Z,Z,Z,Z,Z,Z)- (1TMS)	46.1091	75.1
196	Eicosapentaenoic Acid, TMS derivative*	46.1139	77.1
197	D-(+)-Melibiose MEOX2 TMS	47.1617	243.2
198	D-(+)-Cellobiose, octakis(trimethylsilyl) ether, methyloxime (isomer 1)*	47.19	247.1
199	Laminaribose Meox 1	47.2182	361.2
200	Maltose, 8TMS derivative, isomer 2	47.8244	204.2
201	Isopropyl-beta-D-thiogalactopyranoside (IPTG), O,O,O,O-TMS	48.0235	67.2
202	2-Monooleoylglycerol trimethylsilyl ether*	48.0587	103.1
203	2-oleoylglycerol	48.0628	55.1
204	Silane, tetramethyl-*	48.2653	420.3
205	Maltose, octakis(trimethylsilyl) ether, methyloxime (isomer 1)	48.2853	208.2
206	Maltose (8TMS), MEOX1*	48.297	361.4
207	Maltose Monohydrate MEOX1 TMS*	48.3094	355.2
208	3-alpha-Mannobiose, octakis(trimethylsilyl) ether, methyloxime (isomer 2)*	48.3749	361.2
209	3-alpha-Mannobiose, Octakis(trimethylsilyl) ether, methyloxime (isomer 2)*	48.379	204.2
210	Lactose, 8TMS derivative	48.5133	241.2
211	a,b-Trehalose (8TMS); alpha-D-Glc-(1,1)-beta-D-Glc	48.5188	191.2
212	Nigerose, Meox 1	48.556	306.2
213	3-alpha-Mannobiose, octakis(trimethylsilyl) ether, methyloxime (isomer 1)	48.5643	267.1
214	Palatinose, TMS	48.6738	105.1
215	Maltose Monohydrate MEOX2 TMS*	48.6773	361.3
216	D-(+)-Cellobiose, octakis(trimethylsilyl) ether, methyloxime (isomer 1)*	48.6814	282.1
217	Cellobiose MEOX2 TMS	48.7737	204.1
218	Sophorose (1MEOX) (8TMS) MP	48.9845	319.2
219	3-alpha-Mannobiose, octakis(trimethylsilyl) ether, methyloxime (isomer 2)*	49.0362	271.1
220	Laminaribose Meox 2	49.0417	361.2
221	2-alpha-Mannobiose, octakis(trimethylsilyl) ether, methyloxime (isomer 2)	49.32	205.1
222	Isomaltose MEOX1 TMS	50.1054	204.2
223	Alpha,beta-TREHALOSE TMS	50.8232	361.2
224	2-O-Glycerol-alpha-d-galactopyranoside, hexa-TMS*	52.7548	204.2
225	Cholesterol, TMS derivative*	55.6296	214.3
226	Cholesterol, TMS derivative*	55.6613	129.1
227	Cholesterol, TMS	55.6668	328.4

Ribitol was spiked in each sample as an internal standard. *Some peaks match the spectrum of the same compound, and further confirmation requires authentic standards.

Supplementary Table 2. List of unidentified metabolites by spectral matching

Number	Metabolite	Retention time (min)	Quantitative ion (m/z)	FDR-adjusted *p*-value post ANOVA analysis (Group vs Group)
1	Unknown	9.1349	74.1	> 0.05
2	Unknown	10.4342	40.1	> 0.05
3	Unknown	10.4769	50.5	> 0.05
4	Unknown	10.532	189.1	> 0.05
5	Unknown	10.5837	237.1	> 0.05
6	Unknown	10.5947	158.2	> 0.05
7	Unknown	10.6071	193.2	> 0.05
8	Unknown	10.6422	69.2	> 0.05
9	Unknown	11.4358	134.1	> 0.05
10	Unknown	14.6791	87.7	> 0.05
11	Unknown	18.8407	171.1	> 0.05
12	Unknown	19.2706	265.1	> 0.05
13	Unknown	19.3856	260.2	> 0.05
**14**	**Unknown**	**19.9733**	**219.2**	**0.049362 (CL-HFS vs CL-HFS+ABX)**
15	Unknown	21.1988	100.1	> 0.05
16	Unknown	24.5516	71.2	> 0.05
17	Unknown	24.934	131.1	> 0.05
18	Unknown	24.9429	191.2	> 0.05
**19**	**Unknown**	**26.0569**	**127.1**	**0.008926 (ND vs CL-HFS and CL-HFS vs CL-HFS+ABX)**
20	Unknown	26.1685	86.1	> 0.05
21	Unknown	28.8448	267.1	> 0.05
22	Unknown	30.4106	304.2	> 0.05
23	Unknown	30.4286	299.2	0.0251 (ND vs CL-HFS+ABX)
24	Unknown	30.4472	102.1	> 0.05
25	Unknown	31.807	301.1	> 0.05
26	Unknown	31.8139	299.1	> 0.05
**27**	**Unknown**	**32.5683**	**214.2**	**0.037728 (CL-HFS vs CL-HFS+ABX)**
28	Unknown	32.7556	428.3	> 0.05
29	Unknown	33.4287	74.1	> 0.05
30	Unknown	33.716	114.1	> 0.05
31	Unknown	33.7304	293.1	0.036287 (ND vs CL-HFS and ND vs CL-HFS+ABX)
32	Unknown	33.7428	309.2	> 0.05
33	Unknown	33.8682	45.1	> 0.05
34	Unknown	33.9061	300.2	> 0.05
35	Unknown	34.0942	357.2	> 0.05

36	Unknown	34.1348	280.3	> 0.05
37	Unknown	34.1851	290.3	> 0.05
38	Unknown	34.1906	192.2	> 0.05
39	Unknown	34.2099	274.2	> 0.05
40	Unknown	34.2326	269.2	> 0.05
41	Unknown	34.2374	218.2	> 0.05
42	Unknown	34.2395	305.3	> 0.05
43	Unknown	34.2457	233.2	> 0.05
44	Unknown	34.2705	479.5	> 0.05
45	Unknown	34.606	321.3	> 0.05
46	Unknown	34.75	149.1	> 0.05
47	Unknown	34.7589	220.2	> 0.05
48	Unknown	34.9146	104.1	> 0.05
49	Unknown	35.1109	179.2	> 0.05
**50**	**Unknown**	**35.1185**	**248.1**	**0.021101 (CL-HFS vs CL-HFS+ABX)**
51	Unknown	35.5505	333.3	> 0.05
52	Unknown	35.5553	204.2	> 0.05
53	Unknown	35.558	134.1	> 0.05
54	Unknown	37.1935	265.1	> 0.05
**55**	**Unknown**	**37.9347**	**157.1**	**0.0251 (ND vs CL-HFS, CL-HFS vs CL-HFS+ABX)**
56	Unknown	38.2082	68.2	> 0.05
57	Unknown	39.8781	79.1	0.0251 (ND vs CL-HFS+ABX)
58	Unknown	40.0544	211.1	> 0.05
59	Unknown	40.3341	293.2	> 0.05
60	Unknown	40.4278	129.1	> 0.05
61	Unknown	42.3677	315.1	> 0.05
62	Unknown	42.8989	377.3	> 0.05
63	Unknown	42.9085	238.2	> 0.05
64	Unknown	43.5857	367.3	> 0.05
65	Unknown	44.1582	169.1	> 0.05
66	Unknown	45.8687	122.1	> 0.05
67	Unknown	45.879	204.2	> 0.05
68	Unknown	45.9872	75.1	> 0.05
69	Unknown	47.1369	160.1	> 0.05
**70**	**Unknown**	**48.0704**	**83.2**	**0.0073985 (ND vs CL-HFS and CL-HFS vs CL-HFS+ABX)**
71	Unknown	48.1703	262.2	> 0.05
72	Unknown	48.182	361.3	> 0.05
73	Unknown	48.3025	207.2	> 0.05
74	Unknown	48.5491	377.2	0.0251 (ND vs CL-HFS and ND vs CL-HFS+ABX)
75	Unknown	48.5739	397.4	> 0.05
76	Unknown	48.8839	400.4	> 0.05
77	Unknown	48.8984	400.4	> 0.05

78	Unknown	48.9742	371.2	> 0.05
79	Unknown	48.9825	191.2	> 0.05
80	Unknown	55.6234	248.3	0.039744 (ND vs CL-HFS and ND vs CL-HFS+ABX)
81	Unknown	55.6509	156.2	0.035439 (ND vs CL-HFS and ND vs CL-HFS+ABX)
82	Unknown	55.6592	368.4	0.043583 (ND vs CL-HFS and ND vs CL-HFS+ABX)

These metabolites were not identified by spectral matching. FDR-adjusted *p*-values of the ANOVA analysis with Tukey's multiple comparison test as well as the two-group comparison significance are listed, and the ones that are significantly different ($p < 0.05$) between CL-HFS vs CL-HFS+ABX group are highlighted in this table.

Supplementary Table 3. Clinical information for NASH patients.

Patients with obesity	Age (Year)	Weight (kg)	BMI* (kg/m^2)	AST (U/L)	ALT (U/L)	Glucose (mg/dL)	NAS
Control	42 ± 14	154.6 ± 11.1	55.3 ± 0.5	26 ± 11.2	23.8 ± 7.8	89.3 ± 9.25	1.5 ± 0.58
NASH	40 ± 15	132.3 ± 36.1	49.5 ± 10.3	28 ± 4	38 ± 5.1	116.5 ± 39.9	5.3 ± 0.5

*Abbreviations: AST: aspartate aminotransferase; ALT: alanine aminotransferase BMI: Body mass index; NAS: NAFLD activity score.

Supplementary Table 4. List of primers used in real-time PCR.

Primers	Forward (5’-3’)	Reverse (5’-3’)
Mouse genes		
Acta2	GGCTCTGGGCTCTGTAAGG	CTCTTGCTCTGGGCTTCATC
Adipoq	TTCAGTTACTGCTCGCTGGA	CACCCCTGATGCTCTCCTAG
Cnr1	TCTGCTTGCGATCATGGTGT	GCATGTCTCAGGTCCTTGCT
Ccl2	CCCCAAGAAGGAATGGGTCC	GTGCTGAAGACCTTAGGGCA
Cd68	GACACTTCGGGCCATGTT	GAGGAGGACCAGGCCAAT
Col1a1	CCAAGGGTAACAGCGGTGAA	CCTCGTTTTCCTTCTTCTCCG
Col4a1	TTAAAGGACTCCAGGGACCAC	CCCACTGAGCCTGTCACAC
Gpr119	CTGGCCAATCTGAAGACTACTG	GGTGATTCCAGACTGCTCTGT
IL1b	TCTGAAGCAGCTATGGCAAC	ATGAGTTGGGGACTCTCTGG
Lep	CTGTCTCCCACCCATTCTGT	CCAAGCCCCTTTGTTCATCC
Myd88	CAGTGTCTGGGGGAGGAATG	CAGGTGGAGGAGGTTTACACT
Nfkb	TCCACAAGGCAGCAAATAGA	GGGGCATTTTGTTGAGAGTT
Retn	ACCTCCCCTCACTCCAAAAG	CCTATGCACACACAAGCTCC
Tgfb1	GGTTCATGTCATGGATGGTGC	TGACGTCACTGGAGTTGTACGG
Tnfa	ACGGCATGGATCTCAAAGAC	GTGGGTGAGGAGCACGTAGT
18S	AAGTCCCTGCCCTTTGTACACA	GCCTCACTAAACCATCCAATCG
Human genes		
Acta2	CCGACCGAATGCAGAAGGA	ACAGAGTATTTGCGCTCCGAA
Ccl2	CAGCCAGATGCAATCAATGCC	TGGAATCCTGAACCCACTTCT
Cd68	GAACCCCAACAAAACCAAG	GATGAGAGGCAGCAAGATG
Col1a1	GAGGGCCAAGACGAAGACATC	CAGATCACGTCATCGCACAAC
Col4a1	CAAGAGGATTTCCAGGTCCA	TCATTGCCTTGCACGTAGAG
Gpr119	CTCCCTCATCATTGCTACTAA	ACAGCCAGATTCAAGGTG
IL1b	AGCTACGAATCTCCGACCAC	CGTTATCCCATGTGTCGAAGAA
IL6	ACTCACCTCTTCAGAACGAATTG	CCATCTTTGGAAGGTTCAGGTTG
Myd88	GAAGAAAGAGTTCCCCAGCA	GTGCAGGGGTTGGTGTAGTC
Nfkb	TATGTGGGACCAGCAAAGGT	GCAGATCCCATCCTCACAGT
Tgfb1	CCCAGCATCTGCAAAGCTC	GTCAATGTACAGCTGCCGCA
Tnfa	CCTCTCTCTAATCAGCCCTCTG	GAGGACCTGGGAGTAGATGAG
18S	CTACCACATCCAAGGAAGCA	TTTTTCGTCACTACCTCCCCG
Bacteria		
B. producta	CTTGACATCCCTCTGACCGT	CCTAGAGTGCCCACCATCAT

Supplementary Table 5. List of antibodies for flow cytometry.

Reagent	Company	Dilution	Catalog #
APC anti-mouse/human CD45R/B220 antibody (FACS)	BioLegend	1:100	CAT# 103212
FITC anti-mouse CD3 antibody (FACS)	BioLegend	1:100	CAT# 100204
PE anti-mouse CD4 antibody (FACS)	BioLegend	1:100	CAT# 116006
BV605 anti-mouse CD8a antibody (FACS)	BioLegend	1:100	CAT# 100744
BV605 anti-mouse/human CD11b antibody (FACS)	BioLegend	1:100	CAT# 101257
FITC anti-mouse CD11c antibody (FACS)	BioLegend	1:100	CAT# 117306
BV421 anti-mouse CD45 antibody (FACS)	BioLegend	1:100	CAT# 103133
FITC anti-mouse CD49b (pan-NK cells) antibody (FACS)	BioLegend	1:100	CAT# 108906
APC anti-mouse F4/80 antibody	BioLegend	1:100	CAT# 123116
FITC anti-mouse NK-1.1 antibody	BioLegend	1:100	CAT# 108706
7-AAD Viability staining solution	BioLegend	1:100	CAT# 420404
Fixable Viability Dye eFluor™ 780	Invitrogen	1:1000	CAT# 65-0865-14
FITC anti-mouse collagen I alpha 1 antibody	Novus Biologicals	1:100	CAT# NBP1-77458F
PE anti-mouse alpha-smooth muscle actin antibody (1A4/asm-1)	Novus Biologicals	1:100	CAT# NBP2-34522PE
Alexa Fluor 488 (FITC) anti-mouse GPR119 antibody	Novus Biologicals	1:100	CAT# NLS548AF488
Anti-alpha smooth muscle actin antibody [E184]	Abcam	1:300	CAT# ab32575
