# Supplementary material for: Western diet contributes to the pathogenesis of non-alcoholic steatohepatitis in male mice via remodeling gut microbiota and increasing production of 2-oleoylglycerol
Source: Nat Commun. 2023 Jan 16;14:228. doi: 10.1038/s41467-023-35861-1 (PMC9842745; doi:10.1038/s41467-023-35861-1)
Supplement: Supplementary file 2 — Reporting Summary [file 41467_2023_35861_MOESM2_ESM.pdf]

nature portfolio

Corresponding author(s): Kevin F. Staveley-O'Carroll, Guangfu Li

Last updated by author(s): Dec 18, 2022

Reporting Summary

Nature Portfolio wishes to improve the reproducibility of the work that we publish. This form provides structure for consistency and transparency in reporting. For further information on Nature Portfolio policies, see our Editorial Policies and the Editorial Policy Checklist.

Statistics

For all statistical analyses, confirm that the following items are present in the figure legend, table legend, main text, or Methods section.

n/a	Confirmed	
☐	☒	The exact sample size (*n*) for each experimental group/condition, given as a discrete number and unit of measurement
☐	☒	A statement on whether measurements were taken from distinct samples or whether the same sample was measured repeatedly
☐	☒	The statistical test(s) used AND whether they are one- or two-sided<br>*Only common tests should be described solely by name; describe more complex techniques in the Methods section.*
☒	☐	A description of all covariates tested
☐	☒	A description of any assumptions or corrections, such as tests of normality and adjustment for multiple comparisons
☐	☒	A full description of the statistical parameters including central tendency (e.g. means) or other basic estimates (e.g. regression coefficient) AND variation (e.g. standard deviation) or associated estimates of uncertainty (e.g. confidence intervals)
☐	☒	For null hypothesis testing, the test statistic (e.g. *F*, *t*, *r*) with confidence intervals, effect sizes, degrees of freedom and *P* value noted<br>*Give P values as exact values whenever suitable.*
☒	☐	For Bayesian analysis, information on the choice of priors and Markov chain Monte Carlo settings
☒	☐	For hierarchical and complex designs, identification of the appropriate level for tests and full reporting of outcomes
☒	☐	Estimates of effect sizes (e.g. Cohen's *d*, Pearson's *r*), indicating how they were calculated

Our web collection on statistics for biologists contains articles on many of the points above.

Software and code

Policy information about availability of computer code

Data collection	Real-time PCR analysis: QuantStudio 3 Detection System (Thermo Fisher); Flow cytometry: FACScan Flow Cytometer (BD Biosciences) and Attune NxT Flow Cytometer (Thermo Fisher). 16s RNA gene sequencing (MiSeq, Illunima) and data analysis QIIME2 DADA2 plugin (version 1.10.0); H&E, Sirius red staining, Oil O red staining, IHC staining images: Keyence BZ-X810 microscope; TEM images: JEOL JEM-1400 transmission electron microscope (Japan); AST and ALT analysis in mouse serum: Olympus 400AUe Chemistry Analyzer (Olympus Corporation); metabolite GC-MS analysis: GC System (Agilent 6890) with column (DB-5MS, 60 m, 0.25 mm ID, 0.25 µm, J&W Scientific) and MS system (Agilent 5973 MSD).
Data analysis	Plots and statistics: Graphpadprism v8 (GraphPad Software); Flow cytometry data analysis and display: Flowjo v10.7.1 (BD); IHC image quatification: Image J: semi-quantification (NIH, https://imagej.nih.gov); No custom code was generated in this study.

For manuscripts utilizing custom algorithms or software that are central to the research but not yet described in published literature, software must be made available to editors and reviewers. We strongly encourage code deposition in a community repository (e.g. GitHub). See the Nature Portfolio guidelines for submitting code & software for further information.

Data

Policy information about availability of data

All manuscripts must include a data availability statement. This statement should provide the following information, where applicable:

- Accession codes, unique identifiers, or web links for publicly available datasets
- A description of any restrictions on data availability
- For clinical datasets or third party data, please ensure that the statement adheres to our policy

The source data of plots in figures are provided in Source of data file. The raw 16S rRNA gene sequencing data of mice fecal samples generated in this study have been deposited into NCBI Sequence Read Archive (SRA) with NCBI BioProject ID: PRJNA719798 and free access link: https://www.ncbi.nlm.nih.gov/bioproject/?term=PRJNA719798. The raw data for mouse liver metabolites have been deposited in the NIH Common Fund's National Metabolomics Data Repository (NMDR) with study ID: ST002156. The data can be accessed directly via the project digital object identifier (DOI): http://dx.doi.org/10.21228/M8RQ67 and can be downloaded directly without an access code

Human research participants

Policy information about studies involving human research participants and Sex and Gender in Research.

Reporting on sex and gender	Total 22 patients, including 14 women and 8 men, participated in two studies: 1) hepatic metabolomics assay and 2) human NASH characterization.
Population characteristics	For hepatic metabolomics assay, liver specimens with deidentified information were collected from eight human patients with obesity at the University of Missouri Hospital and frozen at -80 °C. Four out of the patients had histologically confirmed NASH with an average age of 42 and four without NASH with average age of 40.<br>For characterizing human NASH, fourteen slides of formalin-fixed paraffin-embedded (FFPE) human liver biopsies with relevant RNA samples were provided by the University of Kansas Liver Center Biorepository. Seven out of the specimens are from healthy donors with an average age of 45; and another seven specimens are from patients who had histologically confirmed NASH and had no alcohol abuse with an average age of 47.
Recruitment	For hepatic metabolomics assay, eight patients with obesity who did or did not have histologically confirmed NASH were recruited at the University of Missouri Hospital.<br>For characterizing human NASH, seven healthy donors and seven patients with histologically confirmed NASH were recruited from the University of Kansas Liver Center.
Ethics oversight	For hepatic metabolomics assay, the study has been approved by the Institutional Review Board (IRB) of the University of Missouri (IRB#2008258). Written informed consent was obtained from all the study participants.<br>For characterizing human NASH, the study has been approved by the University of Kansas Medical Center Institutional Review Board (IRB# 11378). Written informed consent was obtained from patients.

Note that full information on the approval of the study protocol must also be provided in the manuscript.

Field-specific reporting

Please select the one below that is the best fit for your research. If you are not sure, read the appropriate sections before making your selection.

☒ Life sciences ☐ Behavioural & social sciences ☐ Ecological, evolutionary & environmental sciences

For a reference copy of the document with all sections, see nature.com/documents/nr-reporting-summary-flat.pdf

Life sciences study design

All studies must disclose on these points even when the disclosure is negative.

Sample size	Sample sizes were determined based on pilot studies, or based on prior experience with similar experiments. For experiments involving in vivo efficacy experiments, n=5 mice per group was determined as the minimal sample size. For H&E staining, Oil red O staining, IHC staining, FACS analysis, and qPCR measurements the expression of genes in ex vivo samples, n=5 mice per group was chosen as the minimal sample size. For experiments involving in vitro cell culture, n=3 or 4 per group was chosen as the sample size.
Data exclusions	Gut microbial profiles that were detected from database in human subjects without non-alcoholic fatty liver disease were excluded. The AST and ALT data from a single patient with extremely higher than all the data from the rest subjects were excluded.
Replication	All experiments except the in vivo studies were repeated at least twice. All replication were successful. For in vivo NASH model were successfully established in all tested mice. For the effect of bacteria and gut microbiota profiles, the same group in 5 mice showed similar, which ensure reproducibility same treatment. For H&E staining, Oil red O staining, IHC staining, FACS analysis, and qPCR measurements the expression of genes in ex vivo samples, n=5 mice per group was chosen as the minimal sample size to ensure that reproducibility.

Randomization	Animals were randomly separated to experimental and control groups. Fiver random fields from each slide and random five sections from five mice were used to calculate the average mean of animals of each group were chosen for semi-quantification of Oil red O staining, IHC staining, and Sirius red staining results. For other in vitro experiments , samples were randomly grouped into experimental groups and controls.
Blinding	For the inflammatory cell counting, pathologist unaware of treatment conditions performed cell counting. Quantifications were performed using formulas or software processes applied equally to all conditions. The investigators were not blinded during data collection and outcome assessment, as no subjective assessments were included.

Reporting for specific materials, systems and methods

We require information from authors about some types of materials, experimental systems and methods used in many studies. Here, indicate whether each material, system or method listed is relevant to your study. If you are not sure if a list item applies to your research, read the appropriate section before selecting a response.

Materials & experimental systems

n/a	Involved in the study	
☐	☒	Antibodies
☐	☒	Eukaryotic cell lines
☒	☐	Palaeontology and archaeology
☐	☒	Animals and other organisms
☒	☐	Clinical data
☒	☐	Dual use research of concern

Methods

n/a	Involved in the study	
☒	☐	ChIP-seq
☐	☒	Flow cytometry
☒	☐	MRI-based neuroimaging

Antibodies

Antibodies used	The antibodies used for flow cytometry are listed according to the marker recognized (fluorophore clone name, provider, CatLog number, dilution): APC anti-mouse/human CD45R/B220 Antibody (CAT# 103212, BioLegend, 1:100), FITC anti-mouse CD3 Antibody (CAT# 100204, BioLegend, 1:100), PE anti-mouse CD4 Antibody (CAT# 116006, BioLegend, 1:100), BV605 anti-mouse CD8a Antibody (CAT# 100744, BioLegend, 1:100), BV605 anti-mouse/human CD11b Antibody (CAT# 101257, BioLegend, 1:100), FITC anti-mouse CD11c Antibody (CAT# 117306, BioLegend, 1:100), BV421 anti-mouse CD45 Antibody (CAT# 103133, BioLegend, 1:100), FITC anti-mouse CD49b (pan-NK cells) Antibody (CAT# 108906, BioLegend, 1:100), APC anti-mouse F4/80 Antibody (CAT# 123116, BioLegend, 1:100), FITC anti-mouse NK-1.1 Antibody (CAT# 108706, BioLegend, 1:100), 7-AAD Viability Staining Solution (CAT# 420404, BioLegend, 1:100), Fixable Viability Dye eFluor™ 780 (CAT# 65-0865-14, Invitrogen, 1:1000), FITC anti-mouse collagen I alpha 1 Antibody (CAT# NBP1-77458F, Novus Biologicals, 1:100), PE anti-mouse alpha-smooth muscle actin antibody (1A4/asm-1) (CAT# NBP2-34522PE, Novus Biologicals, 1:100), Alexa Fluor 488 (FITC) anti-mouse GPR119 antibody (NLS548AF488, Novus Biologicals, 1:100), Anti-alpha smooth muscle actin antibody [E184] (CAT# ab32575, Abcam, 1:300). All dilutions were applied according to the manufacturer's suggestion.
Validation	All antibodies in this study were commercially purchased and have been validated by the vendors for species and application. Validation data are available from the respective vendor's respective websites.

Eukaryotic cell lines

Policy information about cell lines and Sex and Gender in Research

Cell line source(s)	RAW264.7 (ATCC TIB-71) was purchased from ATCC (Manassas, VA). Mouse hepatic stellate cell line mHSC is a generous gift from Dr. Scott L. Friedman of the Mount Sinai School of Medicine (NY, USA), available at (Catlog#T0688, Applied Biological Materials Inc, Richmond, Canada). Bacterial strains including Blautia producta (ATCC 27340) and Alistipes putredinis (ATCC 29800) were purchased from ATCC (Manassas, VA).
Authentication	None of the mammalian cell lines have been authenticated in this study. Blautia producta has been verified by 16S rRNA gene sequence, it is 100% match with online database.
Mycoplasma contamination	All cell lines in this study were tested and negative for mycoplasma contamination.
Commonly misidentified lines (See ICLAC register)	No commonly misidentified cell lines were used in this study.

Animals and other research organisms

Policy information about studies involving animals; ARRIVE guidelines recommended for reporting animal research, and Sex and Gender in Research

Laboratory animals	Male C57BL/6J mice, 6-24 weeks old

Laboratory animals	Mice were housed in the animal center with specific pathogen-free(SPF) housing environment, light/dark cycle: 12h/12h, temperature: 65-75°F or 18-23°C, humidity: 40-60%.
Wild animals	No wild animals were used in this study.
Reporting on sex	Male mice were used in this study. The selection of male mice have been discussed in the Discussion section in this study.
Field-collected samples	No field collected samples were used in this study.
Ethics oversight	Animal protocol (Number 24640) was approved by the University of Missouri-Columbia Animal Care and Use Committee. Animal studies complied with all relevant ethical regulations for vertebrate animal research.

Note that full information on the approval of the study protocol must also be provided in the manuscript.

Flow Cytometry

Plots

Confirm that:

☒ The axis labels state the marker and fluorochrome used (e.g. CD4-FITC).

☒ The axis scales are clearly visible. Include numbers along axes only for bottom left plot of group (a 'group' is an analysis of identical markers).

☒ All plots are contour plots with outliers or pseudocolor plots.

☒ A numerical value for number of cells or percentage (with statistics) is provided.

Methodology

Sample preparation	For FACS staining, cells were transferred to a 96-well U-bottom plate, a million cell per well. Each well was washed by 200 µL of FACS buffer (PBS with 2% FBS and 2 mM EDTA), then stained with fluorochrome-conjugated antibodies by diluting the antibody in 50 µL of FACS buffer for 30 min at 4°C. After that, cells were washed and stained with 7-AAD to exclude dead cells when data analysis. For intracellular staining, cells were first stained with fixable viability dye for 30 min at 4°C. Then, cells were washed and stained cell surface markers as the above-mentioned step for fluorochrome-conjugated antibiody staining. After that, cells were wash again and fixed by IC fix butter (Thermo Fisher) at room temperature for 25 min or overnight. Post-fixation, cells were permeablized permeabilization buffer (Thermo Fisher) and stained with a secondary antibody in permeabilization buffer was added for 30 min at 4°C. Cells were washed before analysis.
Instrument	FACScan Flow Cytometer (BD Biosciences) and Attune NxT Flow Cytometer (Thermo Fisher).
Software	Data were analyzed using FlowJo software version 10.7.1 (Tree Star).
Cell population abundance	Each cell frequency in liver non-parenchymal cells was determined by Flow cytometry assay. The total number of cell number were counted using hemocytometer for cells from each group. The abundance of each population was determined by multiplying cell frequency of total cell number for each sample.
Gating strategy	Gating strategies for flow cytometry analysis. Representative flow cytometry figures represent the gating for live hepatic cells. Left: SSC vs FSC gating to exclude debris; Middle: SSC-A vs SSC-H to select single cells; Right: SSC vs viable dye 7-AAD to exclude dead cells. After gating live cells, SSC vs CD45-APC to separate lymphocytes and hepatocytes, or using GPR119-FITC and CD45-APC to gate cells expressing both markers.

☒ Tick this box to confirm that a figure exemplifying the gating strategy is provided in the Supplementary Information.
